# Religious centrality across 22 countries

**DOI:** 10.1038/s41598-025-99183-6

**Published:** 2025-04-30

**Authors:** Robert D. Woodberry, Kathryn A. Johnson, Brendan Case, Matt Bradshaw, Tyler J. VanderWeele, Byron R. Johnson

**Affiliations:** 1 Institute for Studies of Religion, Baylor University, One Bear Place #97326, Waco, TX 76798-7326 USA; 2 Psychology Department, Arizona State University, Tempe, AZ USA; 3 The Human Flourishing Program, Institute for Quantitative Social Science, Harvard University, Cambridge, MA USA; 4 Department of Epidemiology, Harvard T.H. Chan School of Public Health, Boston, MA USA

**Keywords:** Religious centrality, Comparative religion, Secularization theory, Human flourishing, Risk factors, Cultural evolution

## Abstract

Religious Centrality has been widely studied in Europe and North America and is generally associated with better psychological and social outcomes. Religious centrality is often assessed as a measure of intrinsic religiosity (IR)—religion as one’s guiding approach to life – and has been widely validated in societies around the world. However, most studies of religious centrality/IR are cross-sectional and use samples from ‘Western’ societies or samples from single non-European societies. Moreover, most samples are not nationally representative. Systematic comparisons are difficult because the samples, measures, and procedures vary between studies. This article examines religious centrality across 22 nationally representative samples from religiously diverse countries from the 1^st^ wave of the *Global Flourishing Study* (GFS) using a single-item IR measure and identical demographic measures and methods in each country. The study will serve as the foundation for longitudinal studies designed to assess the causal impact of religious centrality on human flourishing. In the current study, we find societies range widely, with religious centrality lowest in Europe and East Asia, moderate in the Americas and Israel, and highest in Africa and the rest of Asia. We discuss the complexities of assessing religious centrality across religious traditions, how the demographic factors associated with religious centrality vary between countries, and provide implications for secularization theory as well as theories of human flourishing.

## Introduction

Religion is an important aspect in many people’s lives and, in most studies, people who say religion is central to their lives have better psychological well-being,^1,2^ lower anxiety and depression,^3,4^ lower prevalence of eating disorders,^5^ and more self-reported charitable giving.^6^ Among college students, those with higher religious centrality scores are also more willing to postpone short-term pleasure for long-term rewards.^7^ One of the most common ways to measure religious centrality is with intrinsic religiosity. Allport’s theory of intrinsic and extrinsic religiosity^8^ is perhaps the single most influential measure of religious centrality in the psychology of religion to date.^9^ Their theory is that for those who have intrinsic religious (IR) motivation, religion is a primary life motive and internalized core value system, whereas for those who have extrinsic religious (ER) motivation, religion is a means to reach other ends, such as status, self-justification, security, social support, and comfort.^10^ IR and ER can be measured as a single continuum, or as distinct concepts that may or may not overlap. In the present research, we adapted a single item from the IR scale to assess religious centrality and its demographic correlates across 22 countries: Specifically, *the extent to which religious beliefs and practices are what really lie behind one’s whole approach to life*.

As with any construct that has been studied so extensively, there are diverse findings on many topics, partially depending on the sample, the type of IR/ER scale, and chance. However, most IR research uses non-random, cross-sectional subsamples of populations in Europe and North America, e.g., undergraduate psychology students. Results from non-probability samples and from sample frames that do not cover the entire population may not generalize to the broader society or to societies in Africa, Asia, and Latin America. IR has been studied outside the West, but it is typically not measured using probability samples that cover the entire country or using consistent methods that allow easy comparison between societies. The paucity of cross-cultural research on religious centrality is hampered somewhat by the Christian-centric items in the many versions of the IR/ER scale. In the present study, we use a modified version of a validated and recommended single item from the longer scale (Gorsuch and McPherson, 1989) with cross-cultural relevance.

The primary contribution of this study is to facilitate accurate cross-national comparisons in the prevalence and demographic correlates of religious centrality—operationalized as one’s religious beliefs and practices guiding one’s life. We use nationally representative data from 22 diverse societies around the globe from the 1^st^ wave of the *Global Flourishing Study* (GFS). The GFS is a longitudinal panel study and will follow the same people through at least three more waves of date collection. The GFS also assesses character strengths, social and political attitudes, and retrospective data about each respondents’ childhoods, but these are analyzed elsewhere. In each country, the GFS employs the same single-item measure of religious centrality (“My religious beliefs and practices are what really lie behind my whole approach to life”), demographic measures, statistical procedures, and models. To our knowledge, this is the first study to compare religious centrality using nationally representative samples from such a diverse set of countries. Previous cross-national comparisons of religiosity using nationally representative samples, such as the *World Values Survey* (WVS), do not measured religiosity centrality in this way.

Of course, comparing any construct across religious traditions and contexts can be difficult,^11^ but our measure of religious centrality (adapted from the IR scale) is likely to have fewer problems than other measures of religious centrality used in other cross-national comparisons. First, IR has been repeatedly validated across different religious traditions and in different countries.^12–20^ This demonstrates it is not merely a Western or Christian concept that does not apply elsewhere. Second, IR, and especially the GFS’s single-item IR question is recommended by previous researchers^21,22^ and seems more content-neutral than many other measures of religiousness, i.e., it does not ask about specific beliefs or behaviors that vary systematically between religious traditions—and yet it includes both beliefs and practices.

Other religion measures are harder to compare between religious contexts because religions have different beliefs and different expectations about, for example, the regularity of public religious participation.^11^ Both the Bible and tradition enjoins Jews and Christians to participate in weekly religious services, and the Quran mandates that Muslim men pray five times a day, communally whenever possible. Conversely, ‘religions’ like Buddhism, Hinduism, Taoism, Shintoism, and Confucianism traditionally had no expectation that devotees participate in weekly public religious services. One can be a devout Shinto, Taoist, or Buddhist – yet attend communal rituals only several times a year.^23–26^ East and South Asian religions sometimes *developed* weekly meetings and articulated beliefs among lay people in response to Christian missions (e.g., in Korea),^27,28^ but these are not universal. Questions about belief in God, heaven, and hell are even more problematic, because some non-Abrahamic religious groups do not have these beliefs. Yet, even for Abrahamic faiths like Islam and Judaism, Christian-based measures may not work well.^20^

There are similar problems with asking respondents about their religious identity because the word ‘religion’ has different meanings in different languages and because the boundaries between ‘religious’ traditions are often unclear. In Japanese, Mandarin, Cantonese, and Korean, the word ‘religion’ is a neologism coined in the nineteenth century, i.e., 宗教 *shūkyō* (Japanese), *zōngjiào* (Mandarin Chinese), zung1gaau3 (Cantonese), and 종교 jong-gyo (Korean). 宗教 is associated with Christianity and was developed in response to Christian missions. It implies an organized religion with clear beliefs and a sacred book, and often carries a negative connotation as foreign and proselytizing.^24–26,29,30^ Thus, many East Asians identify as ‘non-religious’ yet believe in spirits/souls, an afterlife/reincarnation, have ‘god shelves’ in their households, participate in rituals at home and during festivals, etc.^23–26^ The boundaries between religions are also ambiguous, and many participate in multiple ‘traditions’ without ‘belonging’ to any.^29–33^

A second contribution of the present research is the ability to address some discrepancies in the literature. For instance, some research suggests that religious centrality (e.g., IR) has different impact in different religious traditions. For example, in a sample of elderly South Koreans, Protestants’ IR was associated with significantly lower depression and higher quality of life; for Catholics, IR was associated only with higher quality of life; and for Buddhists, IR was not associated with either.^14^ This is not an isolated case. Although not always focused on IR, studies of the impact of religion on mental and physical health find that the associations differ by religious tradition.^34–38^

Still, these divergent findings may be idiosyncratic, e.g., caused by sample variation, and they need to be replicated in more representative samples such as the GFS. Alternatively, diverse findings may be because of reverse causation, e.g., religiosity, religious salience, or religious centrality may increase in response to mental and physical health challenges, especially in traditions that do not have a weekly religious service. Because previous studies are cross-sectional, we cannot determine causal order. However, once later waves of the GFS are collected and processed, we will be able to evaluate multiple hypotheses regarding the influence of religiosity on health, well-being, and character strengths.

Thus, a third and important goal of this study is to create a foundation for future longitudinal analyses about the ‘causal impact’ of religious centrality on *human flourishing* in societies around the world. The present research looks at the demographic correlates of religious centrality cross-nationally. Therefore, the results presented here will serve as a baseline and a foundation for hypothesis generation and later causal analyses using the longitudinal data. Other studies examine the correlates and childhood predictors of 12 indicators of human flourishing. These studies will also help us identify potential common causes of our treatment variables (in this case, religious centrality) and human flourishing in all 22 countries in our sample. We can then measure common causes at wave 1, the treatment at wave 2, and the outcome at wave 3 using marginal structural models (or similar statistical procedures designed to isolate causation). Because of this careful causal ordering, we can avoid adding mediator and collider variables in the analysis.^39–41^ To the best of our knowledge, this type of cross-national, plausibly-causal analysis, with religious variables, has never been attempted before.

Of courses, demonstrating causality is always a challenge, even with longitudinal data. Indeed, there is no assumption-free way to evaluate any social psychological phenomenon. However, longitudinal data (especially with three or more future waves) allows methods that require many fewer assumptions and make causal arguments much more plausible.^42–45^ These models have already been successfully applied to studying the impact of religion in the US.^42,46^ Moreover, we will include measures of robustness, such as the e-score, in all our longitudinal analyses. E-scores shows how much confounding bias would be necessary to remove the association between a treatment variable and an outcome variable.^43^

Finally, many previous cross-national comparisons of religiosity have been used to evaluate secularization theory.^44,45^ There are at least three different ways of thinking about secularity.^47,48^ However, in this article we focus on individual-level change in religious beliefs, practices, and identities, i.e., what Charles Taylor refers to as ‘Secularity II.’^47,48^ In most European societies, there was been a rapid decline in institutional religiosity since at least the mid-twentieth century and various theories have been developed to try to explain it.^49,50^ As the *World Values Survey*, and other surveys, have increasingly collected data from non-European societies, scholars have applied arguments about individual-level secularization to non-European societies as well.^44,45^ However, most of the questions previously used were designed to measure religiosity among Christians and do not work as well with other religious traditions, especially East Asian religious traditions (see the discussion above). Our study was not intended to evaluate secularization theory. However, the cross-national comparisons of the prevalence of religious centrality and the demographic correlates provide evidence about the plausibility of different theories for secularization.

In this paper, we use data from over 200,000 individuals, with nationally representative samples, in 22 culturally and geographically diverse countries, covering all six of the world’s populated continents, to examine demographic variation in religious centrality. These are exploratory and descriptive analyses that create the foundation for future ‘causal analyses.’ Still, the cross-national comparisons are revealing and interesting in themselves. Moreover, we are not aware of any similar cross-national analyses of whether one’s religious beliefs and practices guide one’s life.

### Methods

The description of the methods below was adapted from Vander Weele et al. (2025).^51^ Further methodological details are available elsewhere.^52–57^ All analyses were pre-registered with the Center for Open Science (OSF) prior to data access (10.17605/OSF.IO/CK6UY10.17605/OSF.IO/KX369 and 10.17605/OSF.IO/35XFV). All code to reproduce the analyses are openly available in the OSF repository (10.17605/osf.io/vbype) and all data used in this analysis (Wave 1 of the Global Flourishing Study) are publicly available (10.17605/OSF.IO/3JTZ8).^58^

### Study population

The GFS is a study of 202,898 participants from 22 geographically and culturally diverse countries, with nationally representative sampling within each country. The GFS focuses on the distribution of determinants of well-being. Wave 1 of the data included the following countries and territories: Argentina, Australia, Brazil, Egypt, Germany, Hong Kong (Special Administrative Region of China, with mainland China included from 2024 onwards), India, Indonesia, Israel, Japan, Kenya, Mexico, Nigeria, the Philippines, Poland, South Africa, Spain, Sweden, Tanzania, Turkey, United Kingdom, and the United States. The countries were selected to (a) maximize coverage of the world’s population, (b) ensure geographic, cultural, and religious diversity, and (c) prioritize feasibility and leverage existing data collection infrastructure. Gallup Inc. collected the data. Most Wave 1 data were collected in 2023, although in some countries, data collection began in 2022.^51,53^ Exact dates vary by country.^57^ At least three additional waves of panel data will be collected from 2024–2027.

The sample was designed to ensure nationally representative samples for each of the 21 countries and 1 territory (Hong Kong). Details about the GFS study methodology and survey development are reported elsewhere,^57^ as is information about the sampling and weights.^59^ Survey items were all obtained via self-report and included aspects of well-being such as happiness, health, meaning, character, relationships, and financial stability,^60^ along with other demographic, social, economic, political, religious, personality, childhood, community, health, and well-being variables. During the translation process, Gallup adhered to the TRAPD model (translation, review, adjudication, pretesting, and documentation) for cross-cultural survey research (ccsg.isr.umich.edu/chapters/translation/overview).^44^

### Measures

Demographics Variables. We reclassified continuous ‘age’ as 18–24, 25–29, 30–39, 40–49, 50–59, 60–69, 70–79, and 80 or older. We assessed ‘gender’ as male, female, or other; ‘marital status’ as single/never married, married, separated, divorced, widowed, and domestic partner; and ‘employment’ as employed by others, self-employed, retired, student, homemaker, unemployed and searching, and other. We assessed ‘education’ as up to 8 years, 9–15 years, and 16 + years, and ‘religious service attendance’ as more than once/week, once/week, one to three times/month, a few times/year, or never. We assessed ‘immigration status’ with the questions: “Were you born in this country, or not?” ‘Religious tradition/affiliation’ was assessed using the categories Christianity, Islam, Hinduism, Buddhism, Judaism, Sikhism, Baha’i, Jainism, Shinto, Taoism, Confucianism, Primal/Animist/Folk religion, Spiritism, African-Derived, some other religion, or no religion/atheist/agnostic; although response categories varied by country.^54^ We assessed ‘racial/ethnic identity’ in most countries, but response categories varied by country. The demographic variables are consistent in all GFS Wave 1 papers to allow direct comparison between papers. For additional details on the assessments, see the COS GFS codebook or Crabtree et al. (2021).^61^

Outcome Variable. The survey asked respondents if they agreed or disagreed with the following statement: “My religious beliefs and practices are what really lie behind my whole approach to life.” As discussed below, our religious centrality item was modified from Gorsuch and McPherson’s^51^ intrinsic religiosity item, “My whole approach to life is based on my religion.” We coded responses as *agree* = 1 and *disagree, not relevant,* or *unsure* = 0. Allport and Ross highlight ‘religious centrality’ as the *exemplar* measure of IR (p. 436),^21^ Richard Gorsuch and Susan McPherson argue that it is the *best single measure* of IR when space limitations prevent using a full scale,^22^ and others have validated the utility of single-item measures among Muslims.^62^ There is also some conceptual overlap between our ‘religious centrality’ measure and the *Centrality of Religiosity Scale* (CRS).^63^

IR scales that include ‘religious centrality’ have been widely validated in societies around the world.^22,64^ ‘Religious centrality’ is also part of the widely used 5-item *Duke Religion Index* (DUREL)^65^ that has also been validated in multiple languages, among Muslims, Christians, Jews, Buddhists, and Chinese traditional religionists, and in samples from North America, Latin America, the Muslim World, and East Asia.^12–18^ A different version of the IR scale that includes a similar ‘religious centrality’ measure has also been validated among Muslims in Iran.^19^ Few other measures have undergone such widespread testing with different religious communities in different contexts. Others have validated the utility of this and other single-item measures among Muslims.^62^ Due to space constraints of a large, multi-national, multi-wave study, the GFS opted for the single item IR measure of religious centrality.

Focusing on ‘religious centrality’ avoids the problems associated with treating IR and ER as part of a continuum. Keeping IR and ER distinct is especially important when comparing religious traditions. In North America, there is a significant *negative* correlation between IR and ER among Protestants, a significant *positive* correlation among Jews, and a *non-significant positive* correlation among Catholics.^66^ Among Muslims in Iran, there is also a significant *positive* correlation between IR and ER.^19^ Thus, Allport, Ross, and others now advise *against* treating IR and ER as a single continuum (p. 436).^21^

The GFS also makes some slight modifications to Allport and Ross’s ‘religious centrality’ question, which is “My religious beliefs are what really lie behind my whole approach to life.” Some religious traditions focus more on practices than articulated beliefs^67,68^ and in the cognitive interviews, some groups had difficulty understanding what was meant by ‘religious beliefs.’^54^ Thus, the GFS asks about ‘religious beliefs and practices’ rather than just ‘religious beliefs.’ Moreover, in societies where surveys are less common, many were confused by Likert scales.^54^ Thus, after the cognitive interviews, Gallup dichotomized the question. We note, however, that dichotomizing the response may reduce reported religious centrality in East Asian contexts like Japan.^23^

### Statistical analysis

For each demographic variable, we estimated descriptive statistics for the full sample, with the data weighted to be nationally representative within each country.^59^ We estimated nationally representative proportions for ‘religious centrality’ separately for each country and list them from highest to lowest with their 95% confidence intervals and standard deviations. We also estimated the proportion whose ‘religious beliefs and practices lie behind their whole approach to life’ in each demographic category in each country/territory (see Supplementary Material Tables S1b-S22b). We then conducted a random effects meta-analyses of the country-specific proportions of people who report ‘religious centrality’ for each demographic category,^69,70^ along with 95% confidence intervals, standard errors, and upper and lower limits of the 95% prediction interval. For each response category, we measured variation across countries using the heterogeneity (τ), and I^2^.^71^ Forest plots in the online supplement show the proportions and 95% confidence intervals for each country on each coefficient (Figures S1-S34). We use random effect meta-analysis and random forests because they do not presume cross-cultural measurement equivalence and do not assume the countries are sampled from an identical superpopulation.^34^

We conducted all meta-analyses in **R**^72^ using the metafor package.^73^ Within each country, we conducted a global test of variation across levels of each demographic variable and computed a pooled p-value.^74^ The p-value indicates whether the proportion who report ‘religious centrality’ varies significantly between the response options for that variable in any of the countries in our sample. For example, in Table 3 the global p-value for age is < 0.001, which suggests that age is associated with the proportion of people who report ‘religion is central to their life’ in at least one country. We use Bonferroni corrected p-value thresholds based on the number of demographic variables considered in our analysis.^71,75^ Because different countries have different religious affiliation/traditions and race/ethnicity categories, we do not include them in our meta-analysis. However, we measure them in all the country specific analyses in the online appendix (Tables S1a-S22a and S1b-S22b). In the meta-analysis in the text (Table 3), each country/territory is weighted equally. In the supplementary material, we redo the meta-analysis with countries weighted by their population size (Table S23). We pre-registered all our analyses with COS prior to data access (https://doi.org/10.17605/OSF.IO/CK6UY); all code to reproduce analyses is openly available in an online repository.^57^

### Missing data

We imputed missing data for all variables using multivariate imputation by chained equations and five imputed datasets.^76–79^ The number of missing cases for each variable in each country is reported in Supplementary Material Tables S1a-S22a. Because the categories for religious affiliation/tradition and race/ethnicity varied between countries, we imputed data separately for each country. This within-country imputation approach ensured that the imputation models accurately reflected country-specific contexts and assessment methods. We included sampling weights in the imputation models to account for specific-variable missingness that may have been related to probability of inclusion in the study.

### Accounting for complex sampling design

The GFS used different sampling schemes across countries based on the availability of existing panels and recruitment needs.^57^ All analyses accounted for the complex survey design components by including weights, primary sampling units, and strata. Additional methodological detail, including accounting for the complex sampling design, is provided elsewhere.^53,59^

## Results

Table 1 shows nationally representative descriptive statistics for the demographic characteristics in our sample (all 22 countries combined): 51% are female, 53% are married, 57% are employed or self-employed, 57% have 9–15 years of education, 63% attend religious services, and 94% are native to their country.

**Table 1:** Nationally-Representative Descriptive Statistics of the Observed Sample*

Variable	Proportion	Frequency
Age		
18–24	0.13	27,007
25–29	0.10	20,700
30–39	0.20	40,256
40–49	0.17	34,464
50–59	0.16	31,793
60–69	0.14	27,763
70–79	0.08	16,776
80 or Older	0.02	4119
Missing	0.00	20
Gender		
Male	0.49	98,411
Female	0.51	103,488
Other	0.00	602
Missing	0.00	397
Marital Status		
Single/Never Been Married	0.26	52,115
Married	0.53	107,354
Separated	0.03	5195
Divorced	0.06	11,654
Widowed	0.05	9823
Domestic Partner	0.07	14,931
Missing	0.01	1826
Employment		
Employed for an Employer	0.39	78,815
Self-Employed	0.18	36,362
Retired	0.14	29,303
Student	0.05	10,726
Homemaker	0.11	21,677
Unemployed and Looking for a Job	0.08	16,790
None of These/Other	0.04	8431
Missing	0.00	793
Education		
Up to 8 Years	0.22	45,078
9–15 Years	0.57	115,096
16 + Years	0.21	42,578
Missing	0.00	146
Service Attendance		
> 1/Week	0.13	26,537
1/Week	0.19	39,157
1–3/Month	0.10	19,749
A Few Times a Year	0.20	41,436
Never	0.37	75,297
Missing	0.00	722
Immigration Status		
Born in This Country	0.94	190,998
Born in Another Country	0.05	9791
Missing	0.01	2110
Country		
Argentina	0.03	6724
Australia	0.02	3844
Brazil	0.07	13,204
Egypt	0.02	4729
Germany	0.05	9506
Hong Kong	0.01	3012
India	0.06	12,765
Indonesia	0.03	6992
Israel	0.02	3669
Japan	0.10	20,543
Kenya	0.06	11,389
Mexico	0.03	5776
Nigeria	0.03	6827
Philippines	0.03	5292
Poland	0.05	10,389
South Africa	0.01	2651
Spain	0.03	6290
Sweden	0.07	15,068
Tanzania	0.04	9075
Turkey	0.01	1473
United Kingdom	0.03	5368
United States	0.19	38,312

*Country-specific descriptive statistics are available in the Online Supplement.

Supplemental Tables S1a-S22a show nationally representative descriptive statistics for each country. The most striking difference between countries is the percent who ‘never attend religious service’: from 1% in Nigeria to 77% in Japan; and the percent ‘Atheist, Agnostic, or have no religion’ (*henceforth ‘the non-religious’*), which varies from 0% in Nigeria and Indonesia, to 61% in Japan. However, results in Japan and Hong Kong need to be interpreted with caution. In both societies, respondents often do not categorize Shinto, Shamanism, Taoism, folk religion, or Confucianism as ‘religions.’^23–25^ Still, in many European ancestry societies, the percentage of non-religious is also high. For example, in Australia, 67% ‘never attend’ and 53% are ‘non-religious’.

Table 2 shows the ordered proportions of people in each country who say, “My religious beliefs and practices are what really lie behind my whole approach to life” (i.e., ‘religious centrality’). We should not put too much weight on specific 'rankings;' some confidence intervals overlap, and question wording has different connotations in different languages. However, the general pattern is revealing.

**Table 2:** Ordered Means/Proportions in Religious Centrality for Each Country.

Country	Mean/Proportion	LCI	UCI	SD
Indonesia	0.94	0.93	0.95	0.24
Tanzania	0.91	0.89	0.92	0.29
Egypt	0.90	0.89	0.91	0.30
Nigeria	0.89	0.88	0.90	0.31
India	0.86	0.85	0.87	0.35
Kenya	0.81	0.80	0.83	0.39
South Africa	0.78	0.76	0.80	0.41
Philippines	0.74	0.72	0.76	0.44
Turkey	0.71	0.69	0.74	0.45
Brazil	0.65	0.64	0.66	0.48
Mexico	0.55	0.53	0.56	0.50
Israel	0.46	0.43	0.50	0.50
Argentina	0.46	0.44	0.48	0.50
United States	0.44	0.43	0.45	0.50
Poland	0.44	0.41	0.46	0.50
Hong Kong	0.32	0.30	0.34	0.47
United Kingdom	0.28	0.26	0.30	0.45
Spain	0.27	0.25	0.28	0.44
Australia	0.26	0.24	0.27	0.44
Germany	0.18	0.17	0.19	0.38
Sweden	0.13	0.12	0.13	0.33
Japan	0.07	0.07	0.08	0.26

At the top of the ranking is Indonesia, where 94% say religion is central to their lives (95% confidence interval [CI]: 0.93, 0.95). In Tanzania, religion is central for 91%; in Egypt, 90%; in Nigeria, 89%; and in India, 86%. The confidence intervals of the middle three overlap. Conversely, at the bottom of the ranking is Japan, where only 7% of people say religion is central (95% CI: 0.07, 0.08). Similarly, in Sweden, 13% say religion is central; in Germany, 18%; in Australia, 26%; in Spain, 27%; and in the UK, 28%. The confidence intervals of the last three overlap. In general, wealthy European and East-Asian societies are at the bottom (although Poland and the US are higher up); societies in Africa and the rest of Asia are at the top; and Latin America and Israel are in the middle. The gap in religious centrality between Indonesia and Japan is 87 percentage points.

Table 3 shows random meta-analytic effects for ‘religious centrality’ by demographic category (giving each country an equal weight). The global p-values for every variable are statistically significant past the Bonferroni corrected threshold of p ≤ 0.007, indicating that each variable is associated with ‘religious centrality’ in at least one country. However, a significant global p-value does not necessarily mean the categories in Table 3 are significantly different from each other.

**Table 3:** Random effects meta-analysis of ‘Religious Centrality’ proportions by demographic category.

					Prediction Interval				
Variable	Category	Proportion	95% CI of Proportion	SE Analogue (CI Width/4)	LL	UL	Heterogeneity (τ)	I^2^	Global p-value
Age group									< 0.001**
	18–24	0.49	(0.34,0.65)	0.08	0.05	0.93	0.38	98.8	
	25–29	0.52	(0.36,0.68)	0.08	0.05	0.94	0.39	98.9	
	30–39	0.54	(0.37,0.69)	0.08	0.05	0.93	0.39	98.8	
	40–49	0.57	(0.41,0.71)	0.08	0.06	0.93	0.37	98.8	
	50–59	0.61	(0.44,0.76)	0.08	0.07	0.95	0.38	98.9	
	60–69	0.63	(0.46,0.77)	0.08	0.10	0.95	0.37	98.9	
	70–79	0.65	(0.48,0.78)	0.08	0.11	0.98	0.37	98.9	
	80 or older	0.77	(0.54,0.90)	0.09	0.17	1.00	0.44	99.5	
Gender									< 0.001**
	Male	0.55	(0.39,0.69)	0.07	0.07	0.94	0.36	98.7	
	Female	0.59	(0.43,0.73)	0.07	0.08	0.93	0.36	98.7	
	Other	0.20	(0.02,0.74)	0.18	0.00	1.00	0.92	99.9	
Marital status									< 0.001**
	Married	0.62	(0.46,0.75)	0.07	0.08	0.94	0.34	98.7	
	Separated	0.52	(0.36,0.68)	0.08	0.06	0.98	0.40	98.9	
	Divorced	0.53	(0.38,0.68)	0.08	0.08	0.95	0.37	98.7	
	Widowed	0.65	(0.47,0.79)	0.08	0.04	0.96	0.39	99.0	
	Domestic partner	0.28	(0.09,0.60)	0.13	0.00	1.00	0.65	99.7	
	Single, never married	0.50	(0.35,0.65)	0.08	0.06	0.94	0.37	98.7	
Employment status									< 0.001**
	Employed for an employer	0.54	(0.39,0.69)	0.08	0.06	0.94	0.37	98.8	
	Self-employed	0.58	(0.43,0.72)	0.07	0.11	0.94	0.34	98.6	
	Retired	0.63	(0.47,0.76)	0.07	0.09	0.96	0.35	98.8	
	Student	0.48	(0.32,0.66)	0.09	0.03	0.96	0.42	99.0	
	Homemaker	0.63	(0.48,0.76)	0.07	0.10	0.93	0.33	98.7	
	Unemployed and looking for a job	0.52	(0.37,0.67)	0.08	0.06	0.93	0.37	98.7	
	None of these/other	0.56	(0.39,0.72)	0.08	0.10	0.97	0.40	98.9	
Education									< 0.001**
	Up to 8 years	0.61	(0.45,0.74)	0.07	0.07	0.93	0.36	98.8	
	9–15 years	0.55	(0.40,0.69)	0.07	0.07	0.94	0.36	98.7	
	16 + years	0.55	(0.40,0.69)	0.07	0.09	0.95	0.35	98.7	
Religious service attendance									< 0.001**
	> 1/week	0.89	(0.85,0.92)	0.02	0.55	0.96	0.08	94.4	
	1/week	0.80	(0.75,0.84)	0.02	0.42	0.93	0.11	94.5	
	1–3/month	0.68	(0.59,0.75)	0.04	0.22	0.92	0.20	97.0	
	A few times a year	0.51	(0.38,0.64)	0.06	0.11	0.93	0.31	98.3	
	Never	0.30	(0.18,0.46)	0.07	0.04	0.88	0.34	98.8	
Immigration status									< 0.001**
	Born in this country	0.57	(0.41,0.71)	0.07	0.08	0.94	0.36	98.7	
	Born in another country	0.56	(0.42,0.69)	0.07	0.10	0.96	0.33	98.5	

*Note.* *p < 0.05; **p < 0.007 (Bonferroni corrected threshold).

In Table 3, there are strong positive associations between age and religious centrality and between religious service attendance and religious centrality. Other associations are weaker in the pooled random effects analysis. The point estimate for ‘female’ is higher than for ‘male’ and ‘other’. Regarding marital status, people with a ‘domestic partner’ have lower religious centrality, and ‘widowed’ and ‘married’ people have the highest centrality. Retired people have the highest point estimate among ‘employment statuses.’ People with less than 8 years of education have the highest point estimate, and native-born people do as well.

The strongest predictor of ‘religious centrality’ is ‘religious service attendance.’ Among those who ‘never attend religious services,’ 30% say religion is central to their life, whereas among ‘weekly + attenders’, 89% do. Thus, mean ‘religious centrality’ in 6 countries (the UK, Spain, Australia, Germany, Sweden, and Japan) is lower than mean ‘religious centrality’ for ‘never attenders’ in the pooled sample. Both these results seem important. Even if people never attend ‘religious’ services, they may view religious beliefs and practices as central to all their life, but this is primarily true outside Europe and Japan.

For each demographic category, the τ shows how much the proportion reporting ‘religious centrality’ varies between countries. Most *taus* are moderate (in the 0.30s), but domestic partners have an unusually high τ (0.65), indicating greater variability across countries. Those who never attend religious services have a higher τ (0.34) than those who attend weekly + (0.08). This suggests that at higher levels of attendance, ‘religious centrality’ is more consistent between countries than at lower levels of attendance.

Most studies of religion give Europe-ancestry societies disproportionate weight. This is partially because of data availability, but also because there are many small European societies and each society is given equal weight. If we recalculate the meta-analyses in Table 3 with each country weighted by its population size (which means India has a much greater influence and Europe has much less influence), many groups become more distinct (see Table S23). Now, married people clearly have the highest religious centrality, and domestic partners the lowest. Among employment statuses, retired people have the highest, and students have the lowest. Religious centrality is higher for people with less education and for immigrants.

Supplementary Tables S1b-S22b mirror Table 3 for each country in the sample. We discuss variables with a global p-value of p ≤ 0.05, but unless they clear a p ≤ 0.005 cutoff, treat them as ‘marginally significant’ because of the high number of tests. We also only discuss differences between groups that have *at least 30* respondents, make up *at least 1%* of the national sample, and whose confidence intervals do not overlap with the mean of other groups. These restrictions are very conservative but focus our discussion on differences that are less likely to be random noise.

The association between religious centrality and most demographic factors varies between countries, although some are relatively consistent. In fifteen countries, religion is more central to older people. In the US, the difference between those ‘18–24’ and those ‘80 + ’ is 30 percentage points (29% vs. 59%). In Egypt and Nigeria, there is no association with age. In Israel, younger people think religion is more central, and in others, that pattern is more complex. However, with Wave 1 GFS data we cannot determine if ‘religious centrality’ increases as people age, if recent cohorts are permanently less religious than older cohorts, or both.

Moreover, the association between age and religious centrality is only monotonic in eight countries and the timing of significant jumps in religious centrality seems to correspond with historical events. In Hong Kong the people with the highest religious centrality were born after 1949 (when the Communist party took over mainland China, huge numbers of refugees fled to Hong Kong, and religious groups managed most education and resettlement programs)^80–82^ and before 1989 (when Hong Kong was integrated into the People’s Republic of China), although only this last jump is statistically significant. In Spain, there is no trend in religious centrality for those born after the Franco regime, but religious centrality is significantly higher for those born during the Franco regime (a dictatorship closely allied with the Catholic Church that ended in 1975). In Tanzania there is no trend in religious centrality for those born after the imposition of state socialism and forced collectivization of agriculture in 1973, but religious centrality is significantly higher for those born prior to 1973. In the UK, those who came of age in the 1960s and 1970s have the lowest religious centrality. However, we did not predict these step changes in religious centrality prior to analyzing the data. Moreover, the age groupings are consistent for all countries in every wave-one study in the GFS special collection and the age cut-points were not chosen to correspond with the years we highlighted. Thus, the hypotheses discussed in this paragraph remain speculative and require further research.

In ten countries, gender is non-significant. In the UK, religion is more central to men, and in eleven countries, it is more central to women (Argentina, Australia, Brazil, India, Kenya, Poland, South Africa, Sweden, Tanzania, the US, and marginally in Egypt). In Poland, the US, and South Africa, the male/female differences are quite large (13 percentage points, 8 pp, and 6 pp, respectively).

In most countries, widowed and/or married people have the highest religious centrality, and single and/or cohabiting people have the lowest. In Egypt, Indonesia, Nigeria, Tanzania, and the Philippines, employment status is not significant, but in most other societies, homemakers and retired people have the highest centrality, whereas students have the lowest.

In six countries, education is not significant; in at least seven, religious centrality is negatively associated with education (Argentina, India, Israel, Kenya, Mexico, South Africa, Turkey, and marginally in Brazil, Hong Kong, and Spain), although in Brazil and India it is not monotonic. In three to four it is positively associated with education (the Philippines, the UK, the US, and marginally in Japan) although in the US and UK it is not monotonic. There is a monotonic positive association between education and religious centrality in Australia, although it just misses statistical significance.

In Poland and Sweden the relationship is curved. In Poland, religious centrality is higher for the moderately educated (9–15 years), and in Sweden, it is marginally lower for the moderately educated. Generally, in societies with more education, education is positively associated with religious centrality, and in societies with less education, education is negatively associated with religious centrality. This does not match what we would expect if education consistently undermined religion, as some secularization theories posit.^44,83^ Immigration status is typically non-significant; but in Australia, Spain, Sweden, and the UK, religion is more central for immigrants. In Israel and Mexico, religion is central to the native-born.

In twenty-one societies, religious service attendance is positively associated with religious centrality. However, in Egypt, moderate attenders report the lowest centrality. The gap between ‘non-attenders’ and ‘more than weekly attenders’ is typically large, i.e., the gap is about 50.5 percentage points; and in Australia, Hong Kong, Israel, Poland, Sweden, and the UK, the gaps are over 80 percentage points. In countries where the gap is smaller, it is because even those who ‘never attend’ say religion is central to their life. For example, 71% of ‘never attenders’ in Nigeria say religion is central to their life.

Similarly, ‘religious traditions’ are associated with ‘religious centrality’ in twenty one countries, although we only compare groups with 30 + respondents. The main distinction is between those with a religious identity and those without one. The association in Indonesia is not significant because there are no ‘non-religious’ in the Indonesian sample and because ‘religious centrality’ is so high for all religious groups. Interestingly, in the GFS’s African countries, even the ‘non-religious’ say religion is central to their life (i.e., in Tanzania, 86% do; in Nigeria, 72%; in South Africa, 71%; and in Kenya, 61%). Thus, religion is more central to these ‘non-religious’ than both mean ‘religious centrality’ in twelve countries, and the ‘religious centrality’ of any religious group in Argentina, Australia, Germany, Japan, Mexico, Poland, Spain, or Sweden.

Typically, religion is most central to Muslims and Christians, especially when they are religious minorities. Thus, in Australia, Germany, Israel, Nigeria, Spain, Sweden, and the UK, religion is most central to the Muslim minority. In Japan, religion is most central to the Christian minority; in Egypt, it is marginally more central to Christians than Muslims (p ≤ 0.03, 6 percentage point difference). However, in Argentina, Brazil, Mexico, Poland, Tanzania, and the US, religion is more central to the Christian majority (although in Argentina and Brazil, the confidence intervals overlap with some religious minorities). In Turkey, the Christian minority is too small for comparison.

Conversely, religion is often less central to Buddhists in our sample. Buddhists are lowest in India, and only ahead of the non-religious in Japan (13% central). However, in Hong Kong, Christians and Buddhists have the highest religious centrality (59% and 61%). This might be due to more competition with Christians in Hong Kong (where 25% are Christian) relative to Japan and India (where 2% are).

The forest plots (Supplementary Figures S1-S34) reveal which countries have proportions above or below the meta-analytic mean. In most plots, both the range and the order of countries is roughly consistent with Table 2. More concretely, in Table 2, 94% of Indonesians say religion is central to their life, whereas in Japan, 7% do, a difference of 87 percentage points. In most forest plots, the range is similar (in the mid-80 s to low-90 s). Moreover, in most plots, Indonesia, Tanzania, Egypt, Nigeria, India, and Kenya are near the top, and Germany, Sweden, and Japan are near the bottom.

However, for ‘religious service attendance,’ both the range in religious centrality and the rank order of countries shift radically at different levels of attendance (see Figures S28 and S32). Among those who attend religious services ‘more than once a week,’ the mean ‘religious centrality’ is 89%, and the confidence intervals of most countries overlap with the mean (Figure S28). If we exclude Spain, Japan, and Germany, the range is only 14 percentage points (96% to 82% centrality). If we include them, the range is 42 percentage points, i.e., less than half the range in Table 2. Moreover, ‘weekly + attenders’ from the US and Hong Kong are now ranked 2^nd^ and 4^th^ in religious centrality, both 12 ranks higher than in Table 2. Similarly, Israel and Sweden move up 7 ranks compared to Table 2. Conversely the Philippines moves down 11 ranks and South Africa down 10.

As we move towards lower levels of attendance, the range increases, and country rankings increasingly mirror Table 2 (see Figures S28-S32). For ‘weekly attenders,’ the range is 52 percentage points; for ‘1–3 × per month attenders’ it is 72; for ‘a few times a year attenders’ 82; and for ‘never attenders’ 84 (now the US is ranked 13^th^ and Hong Kong 20^th^). Thus, for countries near the top of Table 2, ‘religious centrality’ shifts only slightly between ‘weekly + attenders’ and ‘never attenders’ (for Indonesia, it shifts 10 percentage points); but for countries in the middle and bottom of Table 2, mean ‘religious centrality’ shifts radically between high attenders and never attenders.

## Discussion

We wish to highlight several findings. First, the data have a huge range and there are several striking patterns in the country rankings. Wealthier countries are near the bottom and poorer ones near the top. European and East Asian societies are near the bottom, Israel and the Americas in the middle, and Africa and the rest of Asia near the top. Buddhist/ Confucian societies are near the bottom, Muslim-majority societies are near the top, and Christian-majority societies are across the whole spectrum.

On the surface, this cross-national pattern seems to fit current secularization theories. Pippa Norris and Ron Inglehart argue that as societies ‘modernize,’ ‘existential security’ increases; therefore, ‘the need for religion’ declines, and each new generation becomes less religious.^44^ A second version of secularization theory argues that as education increases, religion diminishes,^83^ and a third version claims that as social welfare provision increases, religion diminishes.^84^ There is clear evidence of a decline in institutional religiosity in Europe and her settler colonies. However, it is less clear both if this trend applies elsewhere and what has caused this trend. Unfortunately, most evidence used to support specific secularization theories use cross-sectional data from samples dominated by Europe and her settler colonies, e.g., 70% to 100% of the cases in Norris and Englehart’s analyses are European-ancestry societies^44^ and 92.6% of the cases in Pollack and Rosta.^45^ In longitudinal analyses, most samples are even more biased towards European-ancestry societies. Because sample selection is associated with both the dependent variable (secularization) and the independent variables of interest (e.g., GDP, literacy, life expectancy, welfare provision) this creates endogenous selection bias in the analyses.^85^

Even in cross-sectional analyses of ‘Europe-heavy’ samples, when scholars test whether existential security, education, etc., explain *individual-level* differences in religiosity, results are typically negative, weak, or mixed.^86–90^ This is also true for longitudinal analyses of individuals. For ‘existential insecurity’ theory, the only robust longitudinal predictor of religious change is losing a spouse.^90^ Similarly, the longitudinal ‘impact’ of education depends on the measure of religion. For example, in the US, increased education is associated with increased religious service attendance but less literal interpretations of the Bible and shifting to less conservative denominations. Although religiosity often declines when children leave home for the first time, the decline is greater for those who do *not* go to college than for those who do.^91,92^ The relationship between education and religiosity also varies by country.^49,50^

Recent longitudinal analyses evaluating national-level religious changes also undermine previously dominant theories of secularization. The association between of national-level economic development and secularization is unstable and disappears if former communist countries are dropped.^93^ Similarly, either there is no association between changes in national welfare provision and secularization^94–96^ or the effect disappears when the model controls for time^97^—which is consistent with the interpretation that both welfare provision and secularization are increasing in Europe, but there is no causal impact of one on the other. There are also historical reasons to think the association between welfare provision and secularization may be spurious. The historic relationship between church and state in European societies profoundly shaped the extent and method of welfare provision (with state-church Lutheran societies having the greatest state provision of welfare).^98,99^ State subsidies and restrictions of religious competition in Scandinavian Lutheran societies may also influence downstream religious trends.

Thus, the main empirical evidence for global secularization is the longitudinal decline in religiosity between cohorts in most European-ancestry societies and cross-sectional differences in religiosity between countries in more global samples, which suggest a correlation between religiosity and GDP, etc. It is unclear if these two patterns are connected because of consistent causal mechanism. Moreover, explaining these patterns is tricky without careful attention to both history and the measurement of religion and without better longitudinal data. In this article, and many others, a lot of weight rests on Japan. Is religious centrality low in Japan because it is economically developed, etc., or because Japanese understand “religion” differently, or because of some other reason? These questions are beyond the scope if this paper, but we add them to caution against easy, surface-level interpretations of cross-national differences.

In the individual-level GFS data, some patterns could fit a secularization narrative. For example, in most societies, religious centrality is greater for older people than for younger people. Because we only have the first wave of data, we cannot determine if religion becomes more central as people age, if each successive generation has less religious centrality, or both. There is also at least one clear exception; in Israel, younger people have greater religious centrality, and in other societies there is no association.

The GFS evidence about education and religious centrality is mixed. On average, in poorer, less-educated societies, education is negatively correlated with religious centrality. However, in the UK, the US, the Philippines, and Japan, mostly wealthier and more educated societies, education is positively correlated with religious centrality. This suggests there is no inherent relationship between religious centrality and education.

Our demographic data does not support ‘existential security’ as a mechanism either. ‘Married’ seems like the most existentially secure marital status, and widowed, divorced, separated, and ‘domestic partner’ the most ‘insecure’ status. But the married and widowed consistently have the highest religious centrality and those with a domestic partner have the lowest. Similarly, ‘employed by a company or government’ seems like the most secure employment status and ‘unemployed’ the most insecure status. But in most countries, homemakers and the retired have the most religious centrality, and students have the lowest. However, this is *not* a strong critique of the ‘existential security’ theory.

Conversely, the data do not provide strong support for a major competitor to secularization theory, the religious economies theory.^100,101^ This theory posits that religious competition and religious freedom promote religious flourishing. Religious freedom/competition could be one factor, i.e., ‘Religious centrality’ is higher in the US than in Europe (where there is greater regulation of religion). However, societies like India and Egypt have both high religious regulation and high religious centrality. Thus, other factors must also be at play.

One of the most striking patterns is how much national and religious contexts shape religious centrality. In countries where religious centrality is high, it is high for almost every group. Even those who say they have no religious identity and never attend religious services report high religious centrality. However, in societies where religious centrality is low, identifying with a religious group and attending religious services is crucial, e.g., in Hong Kong and the US, the differences between ‘never attenders’ and ‘weekly + ” attenders are very large. Thus, in most societies, religious centrality is comparable among ‘weekly’ and ‘weekly + ’ religious service attenders but radically different among infrequent or non-attenders. This strong contrast *may* influence the association between religious centrality and mental and physical health. We hope to test this when longitudinal GFS data become available.

Of course, there are limitations with this study. First, in future studies, it would be valuable to test the cross-national measurement invariance of the variables in this study.^102^ In this article we use a random effect meta-analysis and random forests because they do not presume cross-cultural measurement equivalence and do not assume the countries are sampled from an identical superpopulation.^53^ Second, a reviewer raised concern about breaking age into ordinal categories rather than treating it as continuous. However, as part of the GFS special collection, we were required to follow a specific model and variable coding. These were pre-registered with the Center for Open Science (https://osf.io/ck6uy) and peer-reviewed separately.^53^ This decision allows direct comparability between all the outcomes in the GFS special collection. Breaking age into ordinal categories may reduce statistical power and make interpreting results more complicated, but also facilitates discovery of non-linear patterns and distinct steps in religious centrality at particular dates. However, because we did not predict these patterns in advance and the age groupings were not designed to measure specific historical breaks, all our speculations about triggers for changes in religious centrality need further testing.

This article is a cross-sectional analysis of the first wave of the GFS. Thus, we make no causal claims, but more waves are coming. Of course, some caution is needed in interpreting cross-national differences as these may be influenced by translation and by differing interpretation of items and response scales. Most analyses of religious centrality’s association with mental and physical health are cross-sectional and based in European-ancestry societies. Virtually all longitudinal studies of changes in religiosity are based in European ancestry societies. However, as more waves of GFS data become available, we can conduct causal analyses of whether religious centrality influences mental and physical health across religious traditions and contexts. We will also be able to investigate potential influences on individual-level changes in religious centrality. These will help us understand both theories of secularization and theories about the relationship between religion and human flourishing.

## Supplementary Information

Supplementary Information.

## Data Availability

The data are publicly available through the Center for Open Science (OSF) (https://doi.org/10.17605/OSF.IO/3JTZ8)0.1 All analyses were pre-registered with OSF prior to data access (https://doi.org/10.17605/OSF.IO/CK6UYhttps://doi.org/10.17605/OSF.IO/KX369 and https://doi.org/10.17605/OSF.IO/35XFV); all code to reproduce analyses are openly available in an online repository (https://doi.org/10.17605/osf.io/vbype).
