# Supplementary material for: Religious centrality across 22 countries
Source: Sci Rep. 2025 Apr 30;15:15081. doi: 10.1038/s41598-025-99183-6 (PMC12043990; doi:10.1038/s41598-025-99183-6)
Supplement: Supplementary file 1 — Supplementary Information. [file 41598_2025_99183_MOESM1_ESM.pdf]

Wave 1, Life Approach, Demographic Characteristics, Online Supplement

Table 1a shows weighted descriptive statistics for all independent variables examined in this study for each country separately using nonimputed Wave 1 data. These include age, gender, marital status, employment, education, religious service attendance, immigration status, and religion. Table 1b shows weighted means/proportions, standard errors, and 95% confidence intervals across all of the demographic variables examined in this study using Wave 1 data for each country. It also includes global F (Wald) test probabilities for the overall joint significance of each set of indicators for the demographic variables. These results were estimated using five imputed datasets. The multiple imputation process included all study variables and sampling weights.

Table 1a: Nationally-Representative Descriptive Statistics of the Observed Sample (Argentina)

Variable	Proportion	Frequency
Age		
18-24	0.16	1108
25-29	0.11	719
30-39	0.21	1432
40-49	0.19	1254
50-59	0.15	1014
60-69	0.11	730
70-79	0.05	356
80 or Older	0.02	112
Missing	.	.
Gender		
Male	0.47	3143
Female	0.53	3542
Other	0.00	21
Missing	0.00	18
Marital Status		
Single/Never Been Married	0.35	2381
Married	0.23	1565
Separated	0.07	455
Divorced	0.05	321
Widowed	0.06	401
Domestic Partner	0.23	1514
Missing	0.01	88
Employment		
Employed for an Employer	0.36	2440
Self-Employed	0.26	1748
Retired	0.11	773
Student	0.05	354
Homemaker	0.10	639
Unemployed and Looking for a Job	0.08	569
None of These/Other	0.03	179
Missing	0.00	22
Education		
Up to 8 Years	0.34	2263
9-15 Years	0.57	3823
16+ Years	0.09	635

Missing	0.00	3
Service Attendance		
>1/Week	0.08	532
1/Week	0.12	773
1-3/Month	0.07	461
A Few Times a Year	0.29	1949
Never	0.44	2982
Missing	0.00	27
Immigration Status		
Born in This Country	0.94	6346
Born in Another Country	0.05	348
Missing	0.00	29
Religion		
Christianity	0.74	4992
Islam	0.00	9
Hinduism	0.00	6
Buddhism	0.01	35
Judaism	0.01	40
Sikhism	0.00	0
Baha'i	.	.
Jainism	.	.
Shinto	.	.
Taoism	0.00	2
Confucianism	0.00	0
Primal, Animist, or Folk Religion	0.00	19
Spiritism	.	.
African-Derived	.	.
Chinese	.	.
Some Other Religion	0.02	156
No Religion/Atheist/Agnostic	0.20	1352
Missing	0.02	111
Race/Ethnicity		
Asian	0.01	43
Black	0.01	95
Indigenous	0.02	129
Mestizo(a)	0.27	1801
Mullato(a)	0.01	75
White	0.51	3406
Other	0.02	104
Missing	0.16	1070

Table 1b: Variations Across Demographic Characteristics (Argentina)

Variable	Mean/Proportion	SE	LCI	UCI	Global p-value
Age					
18-24	0.33	0.02	0.29	0.37	0.00
25-29	0.42	0.03	0.37	0.47	.
30-39	0.43	0.02	0.39	0.46	.
40-49	0.47	0.02	0.43	0.50	.
50-59	0.56	0.02	0.51	0.60	.
60-69	0.54	0.03	0.48	0.59	.
70-79	0.59	0.04	0.51	0.66	.
80 or Older	0.60	0.08	0.45	0.75	.
Gender					
Male	0.42	0.01	0.39	0.44	0.00
Female	0.50	0.01	0.48	0.52	.
Other	0.16	0.08	-0.01	0.34	.
Marital Status					
Single/Never Been Married	0.39	0.01	0.37	0.42	0.00
Married	0.56	0.02	0.53	0.60	.
Separated	0.47	0.03	0.41	0.54	.
Divorced	0.49	0.04	0.42	0.56	.
Widowed	0.60	0.04	0.53	0.68	.
Domestic Partner	0.42	0.02	0.38	0.45	.
Employment					
Employed for an Employer	0.42	0.01	0.40	0.45	0.00
Self-Employed	0.48	0.02	0.44	0.51	.
Retired	0.58	0.03	0.53	0.63	.
Student	0.33	0.03	0.27	0.39	.
Homemaker	0.52	0.03	0.46	0.57	.
Unemployed and Looking for a Job	0.45	0.03	0.39	0.50	.
None of These/Other	0.43	0.05	0.33	0.52	.
Education					
Up to 8 Years	0.55	0.02	0.52	0.59	0.00
9-15 Years	0.42	0.01	0.40	0.44	.
16+ Years	0.39	0.02	0.35	0.43	.
Service Attendance					
>1/Week	0.88	0.02	0.84	0.92	0.00
1/Week	0.75	0.02	0.71	0.80	.
1-3/Month	0.64	0.03	0.58	0.71	.
A Few Times a Year	0.51	0.02	0.48	0.54	.
Never	0.25	0.01	0.23	0.27	.
Immigration Status					
Born in This Country	0.46	0.01	0.44	0.47	0.14
Born in Another Country	0.52	0.04	0.44	0.59	.
Religion					
Christianity	0.54	0.01	0.52	0.56	0.00
Islam	0.69	0.26	-0.15	1.53	.
Hinduism	0.61	0.32	-0.78	2.00	.
Buddhism	0.36	0.09	0.19	0.54	.

Judaism	0.40	0.12	0.16	0.64	.
Sikhism	1.00	.	.	.	.
Baha'i	.	.	.	.	.
Jainism	.	.	.	.	.
Shinto	.	.	.	.	.
Taoism	0.20	0.14	-4.65e+05	465001.22	.
Confucianism	0.00	.	.	.	.
Primal, Animist, or Folk Religion	0.91	0.06	0.77	1.05	.
Spiritism	.	.	.	.	.
African-Derived	.	.	.	.	.
Chinese	.	.	.	.	.
Some Other Religion	0.54	0.06	0.43	0.66	.
No Religion/Atheist/Agnostic	0.17	0.01	0.14	0.20	.
Race/Ethnicity					
Asian	0.55	0.12	0.30	0.79	0.86
Black	0.50	0.08	0.35	0.66	.
Indigenous	0.53	0.06	0.40	0.65	.
Mestizo(a)	0.47	0.02	0.44	0.50	.
Mullato(a)	0.43	0.09	0.24	0.62	.
White	0.45	0.01	0.43	0.47	.
Other	0.44	0.07	0.30	0.58	.

Table 2a: Nationally-Representative Descriptive Statistics of the Observed Sample (Australia)

Variable	Proportion	Frequency
Age		
18-24	0.09	345
25-29	0.07	282
30-39	0.17	641
40-49	0.16	618
50-59	0.18	691
60-69	0.15	589
70-79	0.13	498
80 or Older	0.05	178
Missing	0.00	2
Gender		
Male	0.48	1861
Female	0.50	1941
Other	0.01	36
Missing	0.00	6
Marital Status		
Single/Never Been Married	0.22	855
Married	0.47	1797
Separated	0.04	158
Divorced	0.09	332
Widowed	0.06	215
Domestic Partner	0.12	450
Missing	0.01	38
Employment		
Employed for an Employer	0.49	1881
Self-Employed	0.10	380
Retired	0.24	912
Student	0.05	190
Homemaker	0.04	137
Unemployed and Looking for a Job	0.03	134
None of These/Other	0.05	206
Missing	0.00	4
Education		
Up to 8 Years	0.02	70
9-15 Years	0.63	2434
16+ Years	0.35	1330
Missing	0.00	10
Service Attendance		
>1/Week	0.04	162
1/Week	0.08	299
1-3/Month	0.04	135
A Few Times a Year	0.17	656
Never	0.67	2584
Missing	0.00	7
Immigration Status		
Born in This Country	0.77	2953
Born in Another Country	0.23	885
Missing	0.00	6

Religion		
Christianity	0.41	1592
Islam	0.01	45
Hinduism	0.01	31
Buddhism	0.01	36
Judaism	0.01	26
Sikhism	0.00	8
Baha'i	0.00	7
Jainism	.	.
Shinto	.	.
Taoism	0.00	5
Confucianism	.	.
Primal, Animist, or Folk Religion	0.01	23
Spiritism	.	.
African-Derived	.	.
Chinese	.	.
Some Other Religion	0.01	39
No Religion/Atheist/Agnostic	0.53	2020
Missing	0.00	15
Race/Ethnicity		
Aboriginal	0.01	53
Australian	0.51	1946
Australian /British/European	0.27	1047
Chinese	0.02	75
Indian	0.02	58
Japanese	0.00	1
Malay	0.00	11
Sinhalese	0.00	1
Spanish	0.00	2
Sri Lankan Moor	0.00	1
Sri Lankan Tamil	0.00	7
Vietnamese	0.00	7
Taiwanese/Holo	.	.
Russian	0.00	7
Samoan	0.00	4
New Zealander	0.02	91
Other European	0.09	357
Other	0.04	163
Missing	0.00	14

Table 2b: Variations Across Demographic Characteristics (Australia)

Variable	Mean/Proportion	SE	LCI	UCI	Global p-value
Age					
18-24	0.15	0.03	0.10	0.21	0.00
25-29	0.18	0.03	0.11	0.25	.
30-39	0.17	0.02	0.13	0.21	.
40-49	0.29	0.02	0.24	0.34	.
50-59	0.27	0.02	0.23	0.31	.
60-69	0.28	0.02	0.24	0.32	.
70-79	0.32	0.02	0.28	0.37	.
80 or Older	0.46	0.04	0.38	0.54	.
Gender					
Male	0.22	0.01	0.20	0.25	0.00
Female	0.29	0.01	0.27	0.32	.
Other	0.10	0.05	-0.01	0.20	.
Marital Status					
Single/Never Been Married	0.19	0.02	0.16	0.23	0.00
Married	0.31	0.01	0.29	0.34	.
Separated	0.18	0.04	0.10	0.25	.
Divorced	0.27	0.03	0.21	0.32	.
Widowed	0.39	0.04	0.32	0.47	.
Domestic Partner	0.11	0.02	0.07	0.15	.
Employment					
Employed for an Employer	0.22	0.01	0.20	0.24	0.00
Self-Employed	0.26	0.03	0.21	0.32	.
Retired	0.33	0.02	0.29	0.36	.
Student	0.15	0.04	0.07	0.23	.
Homemaker	0.34	0.05	0.24	0.45	.
Unemployed and Looking for a Job	0.21	0.05	0.12	0.30	.
None of These/Other	0.32	0.04	0.23	0.40	.
Education					
Up to 8 Years	0.21	0.07	0.06	0.36	0.14
9-15 Years	0.25	0.01	0.22	0.27	.
16+ Years	0.28	0.01	0.25	0.30	.
Service Attendance					
>1/Week	0.93	0.03	0.88	0.98	0.00
1/Week	0.87	0.02	0.83	0.92	.
1-3/Month	0.74	0.05	0.64	0.83	.
A Few Times a Year	0.33	0.02	0.29	0.38	.
Never	0.10	0.01	0.08	0.11	.
Immigration Status					
Born in This Country	0.24	0.01	0.22	0.26	0.00
Born in Another Country	0.30	0.02	0.27	0.34	.
Religion					
Christianity	0.48	0.02	0.45	0.51	0.00
Islam	0.58	0.09	0.40	0.76	.
Hinduism	0.30	0.11	0.08	0.52	.
Buddhism	0.47	0.09	0.29	0.66	.
Judaism	0.26	0.08	0.09	0.43	.

Sikhism	0.00	.	.	.	.
Baha'i	0.86	0.12	-0.21	1.93	.
Jainism	.	.	.	.	.
Shinto	.	.	.	.	.
Taoism	0.73	.	.	.	.
Confucianism	.	.	.	.	.
Primal, Animist, or Folk Religion	0.46	0.15	0.12	0.79	.
Spiritism	.	.	.	.	.
African-Derived	.	.	.	.	.
Chinese	.	.	.	.	.
Some Other Religion	0.40	0.12	0.15	0.66	.
No Religion/Atheist/Agnostic	0.06	0.01	0.05	0.07	.
Race/Ethnicity					
Aboriginal	0.27	0.09	0.09	0.44	0.00
Australian	0.25	0.01	0.23	0.28	.
Australian /British/European	0.23	0.02	0.20	0.26	.
Chinese	0.21	0.06	0.09	0.33	.
Indian	0.25	0.07	0.11	0.39	.
Japanese	0.00	.	.	.	.
Malay	0.00	.	.	.	.
Sinhalese	0.00	.	.	.	.
Spanish	0.34	.	.	.	.
Sri Lankan Moor	0.33	0.04	-5.86	6.53	.
Sri Lankan Tamil	0.27	0.22	-0.46	1.00	.
Vietnamese	0.28	0.12	-0.78	1.35	.
Taiwanese/Holo	.	.	.	.	.
Russian	0.28	0.22	-0.45	1.01	.
Samoan	1.00	.	.	.	.
New Zealander	0.12	0.04	0.04	0.21	.
Other European	0.28	0.03	0.23	0.33	.
Other	0.48	0.05	0.38	0.58	.

Table 3a: Nationally-Representative Descriptive Statistics of the Observed Sample (Brazil)

Variable	Proportion	Frequency
Age		
18-24	0.15	1986
25-29	0.11	1468
30-39	0.22	2908
40-49	0.20	2638
50-59	0.16	2131
60-69	0.11	1435
70-79	0.04	510
80 or Older	0.01	126
Missing	.	.
Gender		
Male	0.48	6320
Female	0.52	6820
Other	0.00	35
Missing	0.00	30
Marital Status		
Single/Never Been Married	0.33	4347
Married	0.35	4646
Separated	0.04	594
Divorced	0.07	865
Widowed	0.03	408
Domestic Partner	0.16	2081
Missing	0.02	263
Employment		
Employed for an Employer	0.28	3756
Self-Employed	0.22	2918
Retired	0.12	1536
Student	0.05	624
Homemaker	0.10	1305
Unemployed and Looking for a Job	0.18	2419
None of These/Other	0.03	448
Missing	0.02	199
Education		
Up to 8 Years	0.24	3139
9-15 Years	0.58	7665
16+ Years	0.18	2390
Missing	0.00	10
Service Attendance		
>1/Week	0.18	2386
1/Week	0.17	2272
1-3/Month	0.11	1398
A Few Times a Year	0.30	3978
Never	0.24	3110
Missing	0.00	61
Immigration Status		
Born in This Country	0.96	12688
Born in Another Country	0.01	153
Missing	0.03	363

Religion		
Christianity	0.75	9911
Islam	0.00	6
Hinduism	0.00	1
Buddhism	0.00	37
Judaism	0.00	31
Sikhism	.	.
Baha'i	0.00	2
Jainism	0.00	2
Shinto	0.00	1
Taoism	0.00	2
Confucianism	0.00	6
Primal, Animist, or Folk Religion	0.00	15
Spiritism	0.05	696
African-Derived	0.04	525
Chinese	.	.
Some Other Religion	0.01	144
No Religion/Atheist/Agnostic	0.13	1712
Missing	0.01	113
Race/Ethnicity		
Branca	0.39	5169
Preta	0.12	1615
Parda	0.39	5125
Amarela	0.02	238
Indigena	0.01	131
Other	0.00	61
Missing	0.07	865

Table 3b: Variations Across Demographic Characteristics (Brazil)

Variable	Mean/Proportion	SE	LCI	UCI	Global p-value
Age					
18-24	0.52	0.01	0.50	0.55	0.00
25-29	0.59	0.02	0.56	0.62	.
30-39	0.65	0.01	0.63	0.67	.
40-49	0.68	0.01	0.66	0.70	.
50-59	0.71	0.01	0.69	0.74	.
60-69	0.72	0.02	0.68	0.76	.
70-79	0.76	0.03	0.69	0.83	.
80 or Older	0.66	0.07	0.52	0.80	.
Gender					
Male	0.62	0.01	0.60	0.63	0.00
Female	0.69	0.01	0.67	0.70	.
Other	0.43	0.10	0.23	0.63	.
Marital Status					
Single/Never Been Married	0.58	0.01	0.56	0.60	0.00
Married	0.74	0.01	0.72	0.76	.
Separated	0.64	0.03	0.58	0.69	.
Divorced	0.70	0.02	0.65	0.74	.
Widowed	0.72	0.03	0.65	0.78	.
Domestic Partner	0.58	0.01	0.55	0.61	.
Employment					
Employed for an Employer	0.66	0.01	0.64	0.68	0.00
Self-Employed	0.66	0.01	0.63	0.68	.
Retired	0.73	0.02	0.69	0.77	.
Student	0.52	0.03	0.47	0.57	.
Homemaker	0.70	0.02	0.67	0.73	.
Unemployed and Looking for a Job	0.62	0.01	0.59	0.64	.
None of These/Other	0.57	0.03	0.51	0.63	.
Education					
Up to 8 Years	0.69	0.01	0.66	0.71	0.01
9-15 Years	0.64	0.01	0.63	0.65	.
16+ Years	0.65	0.01	0.62	0.67	.
Service Attendance					
>1/Week	0.88	0.01	0.86	0.89	0.00
1/Week	0.77	0.01	0.75	0.80	.
1-3/Month	0.72	0.01	0.69	0.75	.
A Few Times a Year	0.62	0.01	0.60	0.64	.
Never	0.40	0.01	0.38	0.43	.
Immigration Status					
Born in This Country	0.65	0.01	0.64	0.66	0.66
Born in Another Country	0.63	0.05	0.53	0.73	.
Religion					
Christianity	0.71	0.01	0.70	0.72	0.00
Islam	0.38	0.17	0.00	0.75	.
Hinduism	0.77	0.28	-1.61	3.15	.
Buddhism	0.64	0.12	0.40	0.89	.
Judaism	0.43	0.14	0.14	0.72	.

Sikhism	.	.	.	.	.
Baha'i	0.83	0.22	-1.06	2.71	.
Jainism	1.00	.	.	.	.
Shinto	.	.	.	.	.
Taoism	0.32	0.29	-0.93	1.57	.
Confucianism	0.42	0.35	-1.08	1.91	.
Primal, Animist, or Folk Religion	0.60	0.19	0.17	1.02	.
Spiritism	0.71	0.02	0.66	0.75	.
African-Derived	0.68	0.02	0.64	0.73	.
Chinese	.	.	.	.	.
Some Other Religion	0.57	0.06	0.46	0.69	.
No Religion/Atheist/Agnostic	0.31	0.01	0.28	0.34	.
Race/Ethnicity					
Branca	0.63	0.01	0.61	0.65	0.02
Preta	0.67	0.02	0.64	0.70	.
Parda	0.67	0.01	0.66	0.69	.
Amarela	0.62	0.04	0.55	0.69	.
Indigena	0.63	0.05	0.52	0.74	.
Other	0.58	0.08	0.42	0.75	.

Table 4a: Nationally-Representative Descriptive Statistics of the Observed Sample (Egypt)

Variable	Proportion	Frequency
Age		
18-24	0.20	960
25-29	0.13	607
30-39	0.25	1204
40-49	0.19	897
50-59	0.13	613
60-69	0.08	387
70-79	0.01	54
80 or Older	0.00	7
Missing	.	.
Gender		
Male	0.51	2394
Female	0.49	2334
Other	.	.
Missing	0.00	0
Marital Status		
Single/Never Been Married	0.20	947
Married	0.72	3387
Separated	0.01	39
Divorced	0.02	101
Widowed	0.05	238
Domestic Partner	.	.
Missing	0.00	17
Employment		
Employed for an Employer	0.27	1267
Self-Employed	0.19	892
Retired	0.05	253
Student	0.06	297
Homemaker	0.37	1772
Unemployed and Looking for a Job	0.05	224
None of These/Other	0.00	21
Missing	0.00	3
Education		
Up to 8 Years	0.53	2486
9-15 Years	0.34	1599
16+ Years	0.14	643
Missing	0.00	1
Service Attendance		
>1/Week	0.18	839
1/Week	0.20	960
1-3/Month	0.08	368
A Few Times a Year	0.10	458
Never	0.44	2091
Missing	0.00	12
Immigration Status		
Born in This Country	1.00	4713
Born in Another Country	0.00	16
Missing	0.00	1

Religion		
Christianity	0.03	120
Islam	0.97	4607
Hinduism	.	.
Buddhism	.	.
Judaism	.	.
Sikhism	.	.
Baha'i	.	.
Jainism	.	.
Shinto	.	.
Taoism	0.00	0
Confucianism	.	.
Primal, Animist, or Folk Religion	.	.
Spiritism	.	.
African-Derived	.	.
Chinese	.	.
Some Other Religion	.	.
No Religion/Atheist/Agnostic	.	.
Missing	0.00	1
Race/Ethnicity		
Arab	0.97	4585
Turkish	0.00	9
Greek	0.00	1
Abazas	.	.
Bedouin Arab	0.00	4
Swiss	.	.
Nubian	0.01	27
Other	.	.
Missing	0.02	102

Table 4b: Variations Across Demographic Characteristics (Egypt)

Variable	Mean/Proportion	SE	LCI	UCI	Global p-value
Age					
18-24	0.89	0.01	0.87	0.92	0.03
25-29	0.88	0.02	0.85	0.91	.
30-39	0.89	0.01	0.87	0.91	.
40-49	0.89	0.01	0.86	0.91	.
50-59	0.92	0.01	0.90	0.95	.
60-69	0.95	0.02	0.91	0.98	.
70-79	0.89	0.05	0.80	0.99	.
80 or Older	0.66	0.08	-12.51	13.82	.
Gender					
Male	0.89	0.01	0.87	0.90	0.03
Female	0.91	0.01	0.90	0.92	.
Other	.	.	.	.	.
Marital Status					
Single/Never Been Married	0.88	0.01	0.85	0.90	0.00
Married	0.90	0.01	0.89	0.91	.
Separated	0.98	0.01	0.96	1.00	.
Divorced	0.95	0.02	0.90	0.99	.
Widowed	0.95	0.02	0.91	0.99	.
Domestic Partner	.	.	.	.	.
Employment					
Employed for an Employer	0.89	0.01	0.86	0.91	0.04
Self-Employed	0.90	0.01	0.88	0.93	.
Retired	0.92	0.03	0.87	0.98	.
Student	0.91	0.02	0.87	0.95	.
Homemaker	0.90	0.01	0.89	0.92	.
Unemployed and Looking for a Job	0.86	0.03	0.80	0.92	.
None of These/Other	0.97	0.00	0.96	0.98	.
Education					
Up to 8 Years	0.91	0.01	0.89	0.92	0.18
9-15 Years	0.89	0.01	0.87	0.90	.
16+ Years	0.89	0.02	0.85	0.92	.
Service Attendance					
>1/Week	0.93	0.01	0.91	0.95	0.00
1/Week	0.92	0.01	0.90	0.94	.
1-3/Month	0.86	0.02	0.81	0.91	.
A Few Times a Year	0.89	0.02	0.86	0.93	.
Never	0.88	0.01	0.87	0.90	.
Immigration Status					
Born in This Country	0.90	0.00	0.89	0.91	0.66
Born in Another Country	0.85	0.10	0.56	1.15	.
Religion					
Christianity	0.96	0.02	0.91	1.01	0.03
Islam	0.90	0.00	0.89	0.91	.
Hinduism	.	.	.	.	.
Buddhism	.	.	.	.	.

Judaism	.	.	.	.	.
Sikhism	.	.	.	.	.
Baha'i	.	.	.	.	.
Jainism	.	.	.	.	.
Shinto	.	.	.	.	.
Taoism	0.00	.	.	.	.
Confucianism	.	.	.	.	.
Primal, Animist, or Folk Religion	.	.	.	.	.
Spiritism	.	.	.	.	.
African-Derived	.	.	.	.	.
Chinese	.	.	.	.	.
Some Other Religion	.	.	.	.	.
No Religion/Atheist/Agnostic	.	.	.	.	.
Race/Ethnicity					
Arab	0.90	0.00	0.89	0.91	0.00
Turkish	0.85	0.03	0.76	0.94	.
Greek	1.00	.	.	.	.
Abazas	.	.	.	.	.
Bedouin Arab	0.76	.	.	.	.
Swiss	.	.	.	.	.
Nubian	0.76	.	.	.	.
Other	.	.	.	.	.

Table 5a: Nationally-Representative Descriptive Statistics of the Observed Sample (Germany)

Variable	Proportion	Frequency
Age		
18-24	0.09	829
25-29	0.08	774
30-39	0.15	1438
40-49	0.16	1494
50-59	0.18	1729
60-69	0.20	1915
70-79	0.12	1137
80 or Older	0.02	190
Missing	.	.
Gender		
Male	0.49	4641
Female	0.51	4843
Other	0.00	11
Missing	0.00	11
Marital Status		
Single/Never Been Married	0.28	2627
Married	0.50	4784
Separated	0.02	219
Divorced	0.08	767
Widowed	0.04	409
Domestic Partner	0.07	619
Missing	0.01	81
Employment		
Employed for an Employer	0.52	4950
Self-Employed	0.07	712
Retired	0.26	2480
Student	0.06	605
Homemaker	0.03	251
Unemployed and Looking for a Job	0.03	288
None of These/Other	0.02	204
Missing	0.00	14
Education		
Up to 8 Years	0.02	235
9-15 Years	0.64	6094
16+ Years	0.33	3164
Missing	0.00	13
Service Attendance		
>1/Week	0.03	285
1/Week	0.04	424
1-3/Month	0.06	550
A Few Times a Year	0.25	2362
Never	0.62	5876
Missing	0.00	9
Immigration Status		
Born in This Country	0.92	8722
Born in Another Country	0.08	744
Missing	0.00	40

Religion		
Christianity	0.53	5052
Islam	0.04	351
Hinduism	0.00	12
Buddhism	0.01	51
Judaism	0.00	19
Sikhism	0.00	5
Baha'i	0.00	3
Jainism	.	.
Shinto	0.00	2
Taoism	0.00	0
Confucianism	0.00	4
Primal, Animist, or Folk Religion	0.00	34
Spiritism	.	.
African-Derived	.	.
Chinese	.	.
Some Other Religion	0.01	60
No Religion/Atheist/Agnostic	0.40	3815
Missing	0.01	99
Race/Ethnicity		
No Data	.	.

Table 5b: Variations Across Demographic Characteristics (Germany)

Variable	Mean/Proportion	SE	LCI	UCI	Global p-value
Age					
18-24	0.14	0.02	0.11	0.18	0.00
25-29	0.12	0.01	0.09	0.15	.
30-39	0.17	0.01	0.14	0.19	.
40-49	0.17	0.01	0.15	0.20	.
50-59	0.18	0.01	0.16	0.21	.
60-69	0.19	0.01	0.16	0.21	.
70-79	0.22	0.02	0.19	0.26	.
80 or Older	0.35	0.04	0.26	0.44	.
Gender					
Male	0.18	0.01	0.17	0.19	0.84
Female	0.18	0.01	0.16	0.19	.
Other	0.12	0.11	-0.12	0.37	.
Marital Status					
Single/Never Been Married	0.15	0.01	0.13	0.17	0.00
Married	0.20	0.01	0.19	0.22	.
Separated	0.19	0.03	0.12	0.25	.
Divorced	0.17	0.02	0.13	0.20	.
Widowed	0.22	0.03	0.17	0.27	.
Domestic Partner	0.11	0.01	0.08	0.14	.
Employment					
Employed for an Employer	0.17	0.01	0.15	0.18	0.00
Self-Employed	0.22	0.02	0.18	0.26	.
Retired	0.20	0.01	0.18	0.22	.
Student	0.13	0.02	0.10	0.17	.
Homemaker	0.22	0.03	0.16	0.28	.
Unemployed and Looking for a Job	0.15	0.03	0.10	0.21	.
None of These/Other	0.16	0.03	0.10	0.22	.
Education					
Up to 8 Years	0.18	0.03	0.11	0.24	0.91
9-15 Years	0.18	0.01	0.17	0.19	.
16+ Years	0.18	0.01	0.16	0.19	.
Service Attendance					
>1/Week	0.54	0.04	0.47	0.61	0.00
1/Week	0.57	0.03	0.51	0.63	.
1-3/Month	0.40	0.03	0.34	0.45	.
A Few Times a Year	0.21	0.01	0.19	0.24	.
Never	0.10	0.01	0.09	0.11	.
Immigration Status					
Born in This Country	0.18	0.01	0.17	0.19	0.37
Born in Another Country	0.20	0.02	0.16	0.23	.
Religion					
Christianity	0.23	0.01	0.22	0.25	0.00
Islam	0.45	0.04	0.38	0.52	.
Hinduism	0.26	0.12	-0.26	0.78	.
Buddhism	0.26	0.06	0.13	0.39	.

Judaism	0.23	0.09	0.01	0.46	.
Sikhism	0.43	0.32	-51.48	52.33	.
Baha'i	.	.	.	.	.
Jainism	.	.	.	.	.
Shinto	0.00	.	.	.	.
Taoism	0.00	.	.	.	.
Confucianism	0.00	.	.	.	.
Primal, Animist, or Folk Religion	0.43	0.11	0.19	0.66	.
Spiritism	.	.	.	.	.
African-Derived	.	.	.	.	.
Chinese	.	.	.	.	.
Some Other Religion	0.44	0.10	0.24	0.65	.
No Religion/Atheist/Agnostic	0.08	0.01	0.06	0.09	.
Race/Ethnicity					
No Data	.	.	.	.	.

Table 6a: Nationally-Representative Descriptive Statistics of the Observed Sample (Hong Kong)

Variable	Proportion	Frequency
Age		
18-24	0.07	217
25-29	0.07	198
30-39	0.17	507
40-49	0.19	580
50-59	0.24	711
60-69	0.21	620
70-79	0.05	164
80 or Older	0.00	15
Missing	.	.
Gender		
Male	0.46	1390
Female	0.54	1620
Other	0.00	2
Missing	.	.
Marital Status		
Single/Never Been Married	0.24	723
Married	0.69	2080
Separated	0.01	21
Divorced	0.03	105
Widowed	0.01	45
Domestic Partner	0.01	37
Missing	0.00	1
Employment		
Employed for an Employer	0.68	2056
Self-Employed	0.08	245
Retired	0.14	423
Student	0.02	55
Homemaker	0.04	114
Unemployed and Looking for a Job	0.02	62
None of These/Other	0.01	39
Missing	0.01	18
Education		
Up to 8 Years	0.14	433
9-15 Years	0.67	2031
16+ Years	0.18	547
Missing	.	.
Service Attendance		
>1/Week	0.08	237
1/Week	0.19	567
1-3/Month	0.11	332
A Few Times a Year	0.18	543
Never	0.44	1332
Missing	0.00	1
Immigration Status		
Born in This Country	0.88	2637
Born in Another Country	0.11	321
Missing	0.02	53

Religion		
Christianity	0.25	757
Islam	0.03	86
Hinduism	0.01	20
Buddhism	0.12	349
Judaism	0.00	10
Sikhism	0.00	2
Baha'i	0.00	3
Jainism	0.00	0
Shinto	0.01	19
Taoism	0.03	97
Confucianism	0.00	11
Primal, Animist, or Folk Religion	0.01	27
Spiritism	.	.
African-Derived	.	.
Chinese	0.04	106
Some Other Religion	0.00	4
No Religion/Atheist/Agnostic	0.50	1518
Missing	0.00	5
Race/Ethnicity		
Chinese (Cantonese)	0.64	1930
Chinese (Chaoshan)	0.07	201
Chinese (Fujianese)	0.04	117
Chinese (Hakka)	0.04	121
Chinese (Shanghainese)	0.03	89
Chinese (Other Ethnicity)	0.09	264
East Asian (Korean, Japanese)	0.00	10
Southeast Asian (Filipino, Indonesian, Thailand)	0.02	46
South Asian (Indian, Nepalese, Pakistani)	0.01	17
Taiwanese	0.00	14
White	0.00	15
Other	0.00	4
Missing	0.06	184

Table 6b: Variations Across Demographic Characteristics (Hong Kong)

Variable	Mean/Proportion	SE	LCI	UCI	Global p-value
Age					
18-24	0.26	0.03	0.20	0.32	0.00
25-29	0.20	0.03	0.14	0.27	.
30-39	0.28	0.02	0.23	0.32	.
40-49	0.33	0.02	0.29	0.38	.
50-59	0.36	0.02	0.32	0.40	.
60-69	0.35	0.03	0.29	0.42	.
70-79	0.31	0.08	0.15	0.47	.
80 or Older	1.00	.	.	.	.
Gender					
Male	0.31	0.02	0.28	0.35	0.00
Female	0.33	0.02	0.30	0.36	.
Other	0.00	.	.	.	.
Marital Status					
Single/Never Been Married	0.25	0.02	0.21	0.29	0.00
Married	0.36	0.01	0.33	0.39	.
Separated	0.08	0.07	-0.10	0.27	.
Divorced	0.33	0.08	0.17	0.49	.
Widowed	0.03	0.02	-0.01	0.08	.
Domestic Partner	0.07	0.04	-0.01	0.15	.
Employment					
Employed for an Employer	0.32	0.01	0.29	0.34	0.00
Self-Employed	0.48	0.04	0.39	0.56	.
Retired	0.32	0.05	0.23	0.41	.
Student	0.19	0.06	0.08	0.31	.
Homemaker	0.22	0.06	0.10	0.34	.
Unemployed and Looking for a Job	0.30	0.09	0.11	0.49	.
None of These/Other	0.22	0.09	0.04	0.39	.
Education					
Up to 8 Years	0.43	0.05	0.33	0.52	0.04
9-15 Years	0.30	0.01	0.28	0.33	.
16+ Years	0.30	0.03	0.25	0.35	.
Service Attendance					
>1/Week	0.94	0.02	0.90	0.97	0.00
1/Week	0.66	0.03	0.60	0.72	.
1-3/Month	0.38	0.04	0.31	0.45	.
A Few Times a Year	0.31	0.03	0.25	0.36	.
Never	0.06	0.01	0.04	0.08	.
Immigration Status					
Born in This Country	0.32	0.01	0.30	0.34	0.82
Born in Another Country	0.33	0.05	0.24	0.42	.
Religion					
Christianity	0.59	0.03	0.54	0.64	0.00
Islam	0.73	0.07	0.59	0.87	.
Hinduism	0.30	0.13	0.02	0.59	.
Buddhism	0.61	0.03	0.54	0.68	.

Judaism	0.17	0.10	-0.05	0.39	.
Sikhism	0.82	0.30	-48.60	50.23	.
Baha'i	0.43	0.26	-0.41	1.26	.
Jainism	0.00	.	.	.	.
Shinto	0.95	0.04	0.86	1.05	.
Taoism	0.36	0.08	0.20	0.53	.
Confucianism	0.23	0.13	-0.06	0.51	.
Primal, Animist, or Folk Religion	0.24	0.08	0.07	0.41	.
Spiritism	.	.	.	.	.
African-Derived	.	.	.	.	.
Chinese	0.36	0.07	0.23	0.49	.
Some Other Religion	0.00	.	.	.	.
No Religion/Atheist/Agnostic	0.09	0.01	0.07	0.11	.
Race/Ethnicity					
Chinese (Cantonese)	0.29	0.01	0.26	0.32	0.00
Chinese (Chaoshan)	0.32	0.04	0.25	0.39	.
Chinese (Fujianese)	0.30	0.05	0.20	0.41	.
Chinese (Hakka)	0.29	0.06	0.18	0.41	.
Chinese (Shanghainese)	0.44	0.10	0.24	0.63	.
Chinese (Other Ethnicity)	0.47	0.04	0.38	0.55	.
East Asian (Korean, Japanese)	0.23	0.19	-0.32	0.78	.
Southeast Asian (Filipino, Indonesian, Thailand)	0.67	0.12	0.41	0.93	.
South Asian (Indian, Nepalese, Pakistani)	0.84	0.11	0.56	1.12	.
Taiwanese	0.28	0.15	-0.06	0.61	.
White	0.21	0.12	-0.07	0.48	.
Other	0.32	0.24	-0.47	1.11	.

Table 7a: Nationally-Representative Descriptive Statistics of the Observed Sample (India)

Variable	Proportion	Frequency
Age		
18-24	0.20	2543
25-29	0.13	1640
30-39	0.24	3109
40-49	0.18	2275
50-59	0.12	1574
60-69	0.09	1188
70-79	0.03	370
80 or Older	0.01	67
Missing	.	.
Gender		
Male	0.51	6473
Female	0.49	6292
Other	.	.
Missing	.	.
Marital Status		
Single/Never Been Married	0.16	2065
Married	0.77	9848
Separated	0.00	45
Divorced	0.00	25
Widowed	0.03	445
Domestic Partner	0.02	269
Missing	0.01	69
Employment		
Employed for an Employer	0.21	2660
Self-Employed	0.27	3401
Retired	0.02	286
Student	0.04	532
Homemaker	0.33	4221
Unemployed and Looking for a Job	0.07	902
None of These/Other	0.06	715
Missing	0.00	48
Education		
Up to 8 Years	0.89	11422
9-15 Years	0.09	1194
16+ Years	0.01	145
Missing	0.00	4
Service Attendance		
>1/Week	0.23	2875
1/Week	0.25	3166
1-3/Month	0.21	2740
A Few Times a Year	0.16	2090
Never	0.14	1823
Missing	0.01	71
Immigration Status		
Born in This Country	0.99	12629
Born in Another Country	0.01	110
Missing	0.00	26

Religion		
Christianity	0.02	306
Islam	0.12	1555
Hinduism	0.81	10362
Buddhism	0.02	230
Judaism	.	.
Sikhism	0.01	127
Baha'i	.	.
Jainism	0.00	10
Shinto	0.00	1
Taoism	.	.
Confucianism	.	.
Primal, Animist, or Folk Religion	0.00	30
Spiritism	.	.
African-Derived	.	.
Chinese	.	.
Some Other Religion	0.01	67
No Religion/Atheist/Agnostic	0.00	13
Missing	0.00	62
Race/Ethnicity		
General	0.28	3538
Other Backward Caste	0.33	4177
Schedule Caste	0.28	3599
Schedule Tribe	0.09	1185
Other	.	.
Missing	0.02	267

Table 7b: Variations Across Demographic Characteristics (India)

Variable	Mean/Proportion	SE	LCI	UCI	Global p-value
Age					
18-24	0.82	0.01	0.79	0.84	0.01
25-29	0.86	0.01	0.84	0.87	.
30-39	0.87	0.01	0.86	0.88	.
40-49	0.87	0.01	0.85	0.89	.
50-59	0.87	0.01	0.85	0.89	.
60-69	0.87	0.01	0.84	0.89	.
70-79	0.88	0.03	0.82	0.93	.
80 or Older	0.82	0.03	0.75	0.89	.
Gender					
Male	0.85	0.01	0.83	0.86	0.00
Female	0.87	0.01	0.86	0.88	.
Other	.	.	.	.	.
Marital Status					
Single/Never Been Married	0.79	0.01	0.76	0.81	0.00
Married	0.87	0.00	0.86	0.88	.
Separated	0.75	0.07	0.59	0.90	.
Divorced	0.76	0.02	0.72	0.81	.
Widowed	0.88	0.02	0.84	0.92	.
Domestic Partner	0.76	0.03	0.70	0.83	.
Employment					
Employed for an Employer	0.83	0.01	0.81	0.85	0.00
Self-Employed	0.86	0.01	0.84	0.88	.
Retired	0.84	0.03	0.79	0.90	.
Student	0.77	0.02	0.72	0.81	.
Homemaker	0.87	0.01	0.86	0.89	.
Unemployed and Looking for a Job	0.86	0.01	0.84	0.89	.
None of These/Other	0.88	0.01	0.86	0.91	.
Education					
Up to 8 Years	0.86	0.00	0.86	0.87	0.00
9-15 Years	0.79	0.01	0.76	0.81	.
16+ Years	0.81	0.03	0.75	0.87	.
Service Attendance					
>1/Week	0.91	0.01	0.89	0.92	0.00
1/Week	0.89	0.01	0.87	0.90	.
1-3/Month	0.89	0.01	0.87	0.90	.
A Few Times a Year	0.83	0.01	0.81	0.85	.
Never	0.72	0.01	0.69	0.75	.
Immigration Status					
Born in This Country	0.86	0.00	0.85	0.87	0.16
Born in Another Country	0.91	0.03	0.84	0.97	.
Religion					
Christianity	0.88	0.02	0.83	0.92	0.00
Islam	0.89	0.01	0.87	0.92	.
Hinduism	0.85	0.00	0.84	0.86	.
Buddhism	0.69	0.04	0.60	0.77	.
Judaism	.	.	.	.	.

Sikhism	0.94	0.02	0.91	0.98	.
Baha'i	.	.	.	.	.
Jainism	0.86	0.09	0.08	1.64	.
Shinto	1.00	.	.	.	.
Taoism	.	.	.	.	.
Confucianism	.	.	.	.	.
Primal, Animist, or Folk Religion	0.74	0.11	0.48	1.01	.
Spiritism	.	.	.	.	.
African-Derived	.	.	.	.	.
Chinese	.	.	.	.	.
Some Other Religion	0.89	0.04	0.79	0.99	.
No Religion/Atheist/Agnostic	0.23	.	.	.	.
Race/Ethnicity					
General	0.85	0.01	0.83	0.87	0.06
Other Backward Caste	0.87	0.01	0.86	0.89	.
Schedule Caste	0.85	0.01	0.83	0.86	.
Schedule Tribe	0.85	0.01	0.83	0.87	.
Other	.	.	.	.	.

Table 8a: Nationally-Representative Descriptive Statistics of the Observed Sample (Indonesia)

Variable	Proportion	Frequency
Age		
18-24	0.17	1216
25-29	0.12	849
30-39	0.23	1591
40-49	0.23	1576
50-59	0.17	1169
60-69	0.07	490
70-79	0.01	83
80 or Older	0.00	17
Missing	.	.
Gender		
Male	0.50	3461
Female	0.50	3513
Other	0.00	7
Missing	0.00	11
Marital Status		
Single/Never Been Married	0.20	1381
Married	0.69	4846
Separated	0.01	82
Divorced	0.03	196
Widowed	0.06	425
Domestic Partner	0.00	18
Missing	0.01	45
Employment		
Employed for an Employer	0.19	1323
Self-Employed	0.31	2187
Retired	0.01	78
Student	0.04	272
Homemaker	0.31	2138
Unemployed and Looking for a Job	0.08	529
None of These/Other	0.06	448
Missing	0.00	18
Education		
Up to 8 Years	0.44	3079
9-15 Years	0.50	3491
16+ Years	0.06	419
Missing	0.00	2
Service Attendance		
>1/Week	0.38	2667
1/Week	0.36	2529
1-3/Month	0.11	786
A Few Times a Year	0.09	659
Never	0.05	332
Missing	0.00	18
Immigration Status		
Born in This Country	1.00	6958
Born in Another Country	0.00	34
Missing	.	.

Religion		
Christianity	0.07	504
Islam	0.92	6406
Hinduism	0.01	73
Buddhism	0.00	3
Judaism	.	.
Sikhism	.	.
Baha'i	.	.
Jainism	.	.
Shinto	.	.
Taoism	0.00	1
Confucianism	.	.
Primal, Animist, or Folk Religion	.	.
Spiritism	.	.
African-Derived	.	.
Chinese	.	.
Some Other Religion	0.00	1
No Religion/Atheist/Agnostic	.	.
Missing	0.00	4
Race/Ethnicity		
Banjar/Melayu Banjar	0.05	320
Betawi	0.04	251
Bugis	0.03	243
Jawa	0.41	2846
Madura	0.04	262
Minangkabau	0.04	273
Sunda/Parahyangan	0.17	1172
Bali	0.01	69
Batak	0.02	165
Makasar	0.01	91
Other	0.18	1262
Missing	0.01	38

Table 8b: Variations Across Demographic Characteristics (Indonesia)

Variable	Mean/Proportion	SE	LCI	UCI	Global p-value
Age					
18-24	0.93	0.01	0.92	0.95	0.00
25-29	0.95	0.01	0.93	0.96	.
30-39	0.93	0.01	0.91	0.94	.
40-49	0.94	0.01	0.92	0.95	.
50-59	0.95	0.01	0.93	0.96	.
60-69	0.95	0.02	0.91	0.98	.
70-79	0.98	0.02	0.95	1.01	.
80 or Older	1.00	.	.	.	.
Gender					
Male	0.94	0.01	0.93	0.95	0.00
Female	0.93	0.01	0.92	0.94	.
Other	1.00	.	.	.	.
Marital Status					
Single/Never Been Married	0.94	0.01	0.92	0.95	0.00
Married	0.94	0.00	0.93	0.95	.
Separated	0.90	0.05	0.80	0.99	.
Divorced	0.95	0.02	0.91	0.99	.
Widowed	0.93	0.02	0.90	0.97	.
Domestic Partner	1.00	.	.	.	.
Employment					
Employed for an Employer	0.95	0.01	0.93	0.96	0.33
Self-Employed	0.94	0.01	0.93	0.95	.
Retired	0.96	0.02	0.92	1.00	.
Student	0.96	0.01	0.93	0.99	.
Homemaker	0.93	0.01	0.91	0.94	.
Unemployed and Looking for a Job	0.93	0.02	0.90	0.96	.
None of These/Other	0.94	0.01	0.92	0.97	.
Education					
Up to 8 Years	0.94	0.01	0.92	0.95	0.25
9-15 Years	0.94	0.00	0.93	0.95	.
16+ Years	0.95	0.01	0.94	0.97	.
Service Attendance					
>1/Week	0.96	0.00	0.95	0.97	0.00
1/Week	0.93	0.01	0.92	0.94	.
1-3/Month	0.93	0.01	0.90	0.95	.
A Few Times a Year	0.93	0.01	0.91	0.95	.
Never	0.86	0.02	0.81	0.91	.
Immigration Status					
Born in This Country	0.94	0.00	0.93	0.95	0.59
Born in Another Country	0.96	0.04	0.87	1.05	.
Religion					
Christianity	0.96	0.01	0.94	0.97	0.00
Islam	0.94	0.00	0.93	0.94	.
Hinduism	0.94	0.01	0.92	0.97	.
Buddhism	1.00	.	.	.	.
Judaism	.	.	.	.	.

Sikhism	.	.	.	.	.
Baha'i	.	.	.	.	.
Jainism	.	.	.	.	.
Shinto	.	.	.	.	.
Taoism	1.00	.	.	.	.
Confucianism	.	.	.	.	.
Primal, Animist, or Folk Religion	.	.	.	.	.
Spiritism	.	.	.	.	.
African-Derived	.	.	.	.	.
Chinese	.	.	.	.	.
Some Other Religion	1.00	.	.	.	.
No Religion/Atheist/Agnostic	.	.	.	.	.
Race/Ethnicity					
Banjar/Melayu Banjar	0.92	0.02	0.89	0.96	0.14
Betawi	0.93	0.02	0.89	0.98	.
Bugis	0.94	0.02	0.90	0.97	.
Jawa	0.94	0.00	0.93	0.95	.
Madura	0.93	0.02	0.88	0.98	.
Minangkabau	0.93	0.02	0.88	0.98	.
Sunda/Parahyangan	0.93	0.01	0.90	0.95	.
Bali	0.95	0.01	0.93	0.98	.
Batak	0.95	0.02	0.92	0.98	.
Makasar	0.97	0.01	0.95	0.99	.
Other	0.94	0.01	0.92	0.96	.

Table 9a: Nationally-Representative Descriptive Statistics of the Observed Sample (Israel)

Variable	Proportion	Frequency
Age		
18-24	0.15	553
25-29	0.11	407
30-39	0.18	666
40-49	0.17	616
50-59	0.15	542
60-69	0.13	469
70-79	0.09	336
80 or Older	0.02	79
Missing	.	.
Gender		
Male	0.49	1791
Female	0.51	1872
Other	0.00	0
Missing	0.00	6
Marital Status		
Single/Never Been Married	0.23	834
Married	0.56	2056
Separated	0.01	48
Divorced	0.07	258
Widowed	0.06	212
Domestic Partner	0.05	193
Missing	0.02	69
Employment		
Employed for an Employer	0.49	1793
Self-Employed	0.12	424
Retired	0.16	576
Student	0.11	388
Homemaker	0.06	211
Unemployed and Looking for a Job	0.04	148
None of These/Other	0.03	118
Missing	0.00	10
Education		
Up to 8 Years	0.06	224
9-15 Years	0.41	1517
16+ Years	0.52	1926
Missing	0.00	2
Service Attendance		
>1/Week	0.18	649
1/Week	0.14	495
1-3/Month	0.10	374
A Few Times a Year	0.28	1014
Never	0.31	1122
Missing	0.00	14
Immigration Status		
Born in This Country	0.76	2796
Born in Another Country	0.24	868
Missing	0.00	5

Religion		
Christianity	0.01	39
Islam	0.18	656
Hinduism	.	.
Buddhism	.	.
Judaism	0.79	2897
Sikhism	.	.
Baha'i	0.00	2
Jainism	.	.
Shinto	.	.
Taoism	0.00	1
Confucianism	.	.
Primal, Animist, or Folk Religion	0.00	1
Spiritism	.	.
African-Derived	.	.
Chinese	.	.
Some Other Religion	0.00	5
No Religion/Atheist/Agnostic	0.02	64
Missing	0.00	4
Race/Ethnicity		
Jewish	0.80	2926
Arab	0.18	674
Other	0.01	39
Missing	0.01	30

Table 9b: Variations Across Demographic Characteristics (Israel)

Variable	Mean/Proportion	SE	LCI	UCI	Global p-value
Age					
18-24	0.57	0.03	0.51	0.62	0.00
25-29	0.47	0.03	0.41	0.53	.
30-39	0.46	0.03	0.40	0.51	.
40-49	0.46	0.03	0.41	0.52	.
50-59	0.44	0.03	0.38	0.49	.
60-69	0.45	0.03	0.39	0.51	.
70-79	0.37	0.03	0.31	0.43	.
80 or Older	0.35	0.06	0.22	0.48	.
Gender					
Male	0.46	0.02	0.42	0.50	0.00
Female	0.46	0.02	0.42	0.50	.
Other	0.00	.	.	.	.
Marital Status					
Single/Never Been Married	0.49	0.03	0.44	0.54	0.00
Married	0.50	0.02	0.46	0.54	.
Separated	0.43	0.03	0.36	0.51	.
Divorced	0.31	0.04	0.24	0.38	.
Widowed	0.46	0.04	0.38	0.55	.
Domestic Partner	0.17	0.03	0.11	0.24	.
Employment					
Employed for an Employer	0.43	0.02	0.39	0.46	0.00
Self-Employed	0.42	0.03	0.37	0.48	.
Retired	0.42	0.03	0.36	0.48	.
Student	0.61	0.04	0.54	0.69	.
Homemaker	0.67	0.05	0.56	0.77	.
Unemployed and Looking for a Job	0.48	0.06	0.36	0.59	.
None of These/Other	0.46	0.04	0.37	0.55	.
Education					
Up to 8 Years	0.60	0.05	0.49	0.70	0.00
9-15 Years	0.52	0.02	0.48	0.57	.
16+ Years	0.40	0.02	0.36	0.43	.
Service Attendance					
>1/Week	0.94	0.01	0.91	0.96	0.00
1/Week	0.81	0.03	0.75	0.86	.
1-3/Month	0.64	0.03	0.58	0.70	.
A Few Times a Year	0.32	0.02	0.27	0.37	.
Never	0.10	0.01	0.07	0.13	.
Immigration Status					
Born in This Country	0.51	0.02	0.47	0.55	0.00
Born in Another Country	0.31	0.02	0.26	0.35	.
Religion					
Christianity	0.42	0.03	0.32	0.51	0.00
Islam	0.66	0.03	0.59	0.73	.
Hinduism	.	.	.	.	.
Buddhism	.	.	.	.	.
Judaism	0.42	0.02	0.39	0.46	.

Sikhism	.	.	.	.	.
Baha'i	1.00	.	.	.	.
Jainism	.	.	.	.	.
Shinto	.	.	.	.	.
Taoism	1.00	.	.	.	.
Confucianism	.	.	.	.	.
Primal, Animist, or Folk Religion	0.00	.	.	.	.
Spiritism	.	.	.	.	.
African-Derived	.	.	.	.	.
Chinese	.	.	.	.	.
Some Other Religion	0.50	.	.	.	.
No Religion/Atheist/Agnostic	0.13	0.04	0.03	0.23	.
Race/Ethnicity					
Jewish	0.42	0.02	0.38	0.46	0.00
Arab	0.65	0.03	0.59	0.72	.
Other	0.22	0.06	0.09	0.36	.

Table 10a: Nationally-Representative Descriptive Statistics of the Observed Sample (Japan)

Variable	Proportion	Frequency
Age		
18-24	0.08	1589
25-29	0.04	806
30-39	0.14	2851
40-49	0.16	3363
50-59	0.18	3770
60-69	0.20	4118
70-79	0.17	3554
80 or Older	0.02	493
Missing	.	.
Gender		
Male	0.48	9847
Female	0.52	10602
Other	0.00	28
Missing	0.00	66
Marital Status		
Single/Never Been Married	0.24	5004
Married	0.58	11837
Separated	0.01	190
Divorced	0.10	2126
Widowed	0.06	1179
Domestic Partner	0.01	144
Missing	0.00	64
Employment		
Employed for an Employer	0.53	10853
Self-Employed	0.09	1748
Retired	0.12	2535
Student	0.02	491
Homemaker	0.06	1276
Unemployed and Looking for a Job	0.03	622
None of These/Other	0.15	2983
Missing	0.00	36
Education		
Up to 8 Years	0.03	567
9-15 Years	0.72	14893
16+ Years	0.25	5083
Missing	.	.
Service Attendance		
>1/Week	0.02	316
1/Week	0.02	348
1-3/Month	0.04	862
A Few Times a Year	0.15	3112
Never	0.77	15788
Missing	0.01	117
Immigration Status		
Born in This Country	0.95	19548
Born in Another Country	0.01	158
Missing	0.04	837

Religion		
Christianity	0.02	381
Islam	0.00	10
Hinduism	0.00	5
Buddhism	0.33	6709
Judaism	0.00	10
Sikhism	0.00	6
Baha'i	0.00	2
Jainism	0.00	11
Shinto	0.02	469
Taoism	0.00	7
Confucianism	0.00	17
Primal, Animist, or Folk Religion	0.00	19
Spiritism	.	.
African-Derived	.	.
Chinese	.	.
Some Other Religion	0.00	46
No Religion/Atheist/Agnostic	0.61	12497
Missing	0.02	355
Race/Ethnicity		
No Data	.	.

Table 10b: Variations Across Demographic Characteristics (Japan)

Variable	Mean/Proportion	SE	LCI	UCI	Global p-value
Age					
18-24	0.05	0.01	0.04	0.07	0.00
25-29	0.05	0.01	0.03	0.07	.
30-39	0.05	0.01	0.04	0.06	.
40-49	0.05	0.00	0.04	0.06	.
50-59	0.06	0.00	0.06	0.07	.
60-69	0.09	0.01	0.08	0.11	.
70-79	0.10	0.01	0.09	0.12	.
80 or Older	0.16	0.02	0.12	0.21	.
Gender					
Male	0.07	0.00	0.06	0.08	0.13
Female	0.08	0.00	0.07	0.09	.
Other	0.09	0.05	-0.01	0.19	.
Marital Status					
Single/Never Been Married	0.06	0.00	0.05	0.06	0.00
Married	0.08	0.00	0.07	0.08	.
Separated	0.06	0.02	0.02	0.11	.
Divorced	0.08	0.01	0.06	0.10	.
Widowed	0.14	0.01	0.11	0.17	.
Domestic Partner	0.04	0.02	0.00	0.08	.
Employment					
Employed for an Employer	0.06	0.00	0.06	0.07	0.00
Self-Employed	0.10	0.01	0.09	0.12	.
Retired	0.09	0.01	0.07	0.10	.
Student	0.03	0.01	0.02	0.05	.
Homemaker	0.10	0.01	0.08	0.11	.
Unemployed and Looking for a Job	0.06	0.01	0.04	0.07	.
None of These/Other	0.09	0.01	0.08	0.11	.
Education					
Up to 8 Years	0.07	0.01	0.04	0.10	0.02
9-15 Years	0.07	0.00	0.07	0.08	.
16+ Years	0.09	0.00	0.08	0.10	.
Service Attendance					
>1/Week	0.60	0.03	0.54	0.67	0.00
1/Week	0.41	0.03	0.35	0.48	.
1-3/Month	0.21	0.02	0.18	0.25	.
A Few Times a Year	0.11	0.01	0.10	0.12	.
Never	0.04	0.00	0.04	0.05	.
Immigration Status					
Born in This Country	0.07	0.00	0.07	0.08	0.47
Born in Another Country	0.10	0.03	0.03	0.17	.
Religion					
Christianity	0.60	0.03	0.54	0.66	0.00
Islam	0.37	0.22	-0.17	0.90	.
Hinduism	0.34	0.28	-0.61	1.28	.
Buddhism	0.13	0.00	0.12	0.14	.
Judaism	0.05	0.06	-0.15	0.26	.

Sikhism	0.00	.	.	.	.
Baha'i	0.00	.	.	.	.
Jainism	0.00	.	.	.	.
Shinto	0.26	0.02	0.22	0.31	.
Taoism	0.04	0.04	-0.06	0.14	.
Confucianism	0.13	0.09	-0.06	0.32	.
Primal, Animist, or Folk Religion	0.22	0.12	-0.03	0.47	.
Spiritism	.	.	.	.	.
African-Derived	.	.	.	.	.
Chinese	.	.	.	.	.
Some Other Religion	0.32	0.08	0.16	0.48	.
No Religion/Atheist/Agnostic	0.02	0.00	0.02	0.03	.
Race/Ethnicity					
No Data	.	.	.	.	.

Table 11a: Nationally-Representative Descriptive Statistics of the Observed Sample (Kenya)

Variable	Proportion	Frequency
Age		
18-24	0.25	2868
25-29	0.18	2035
30-39	0.23	2564
40-49	0.15	1708
50-59	0.09	1072
60-69	0.06	710
70-79	0.03	360
80 or Older	0.01	67
Missing	0.00	5
Gender		
Male	0.49	5567
Female	0.51	5813
Other	0.00	2
Missing	0.00	7
Marital Status		
Single/Never Been Married	0.31	3531
Married	0.58	6626
Separated	0.04	467
Divorced	0.01	111
Widowed	0.04	464
Domestic Partner	0.01	146
Missing	0.00	43
Employment		
Employed for an Employer	0.13	1467
Self-Employed	0.32	3630
Retired	0.03	319
Student	0.10	1136
Homemaker	0.13	1537
Unemployed and Looking for a Job	0.28	3153
None of These/Other	0.01	138
Missing	0.00	9
Education		
Up to 8 Years	0.39	4485
9-15 Years	0.54	6115
16+ Years	0.07	783
Missing	0.00	6
Service Attendance		
>1/Week	0.24	2774
1/Week	0.53	6063
1-3/Month	0.11	1219
A Few Times a Year	0.08	855
Never	0.04	465
Missing	0.00	13
Immigration Status		
Born in This Country	0.99	11270
Born in Another Country	0.01	117
Missing	0.00	2

Religion		
Christianity	0.91	10334
Islam	0.08	918
Hinduism	.	.
Buddhism	0.00	1
Judaism	0.00	3
Sikhism	.	.
Baha'i	0.00	1
Jainism	0.00	1
Shinto	.	.
Taoism	.	.
Confucianism	0.00	3
Primal, Animist, or Folk Religion	0.00	7
Spiritism	.	.
African-Derived	.	.
Chinese	.	.
Some Other Religion	0.00	5
No Religion/Atheist/Agnostic	0.01	108
Missing	0.00	9
Race/Ethnicity		
Luhya	0.17	1943
Luo	0.10	1120
Kalenjin	0.12	1377
Kamba	0.11	1299
Kikuyu	0.19	2118
Kisii	0.07	789
Maasai	0.02	237
Meru	0.06	630
Kenan Somali/Somali	0.03	396
Miji Kenda Tribes	0.06	708
Embu	0.02	197
Other	0.05	548
Missing	0.00	27

Table 11b: Variations Across Demographic Characteristics (Kenya)

Variable	Mean/Proportion	SE	LCI	UCI	Global p-value
Age					
18-24	0.75	0.01	0.73	0.77	0.00
25-29	0.78	0.01	0.76	0.81	.
30-39	0.83	0.01	0.81	0.85	.
40-49	0.85	0.01	0.82	0.87	.
50-59	0.87	0.02	0.84	0.90	.
60-69	0.87	0.02	0.83	0.91	.
70-79	0.90	0.03	0.85	0.96	.
80 or Older	0.85	0.08	0.69	1.02	.
Gender					
Male	0.80	0.01	0.78	0.81	0.00
Female	0.83	0.01	0.81	0.84	.
Other	1.00	.	.	.	.
Marital Status					
Single/Never Been Married	0.77	0.01	0.75	0.79	0.00
Married	0.84	0.01	0.82	0.85	.
Separated	0.80	0.03	0.75	0.86	.
Divorced	0.71	0.06	0.58	0.83	.
Widowed	0.87	0.03	0.82	0.92	.
Domestic Partner	0.72	0.05	0.63	0.81	.
Employment					
Employed for an Employer	0.80	0.01	0.77	0.83	0.00
Self-Employed	0.84	0.01	0.82	0.85	.
Retired	0.86	0.03	0.80	0.92	.
Student	0.76	0.01	0.73	0.79	.
Homemaker	0.86	0.01	0.83	0.89	.
Unemployed and Looking for a Job	0.79	0.01	0.77	0.81	.
None of These/Other	0.76	0.04	0.68	0.84	.
Education					
Up to 8 Years	0.85	0.01	0.83	0.87	0.00
9-15 Years	0.79	0.01	0.78	0.81	.
16+ Years	0.77	0.02	0.73	0.82	.
Service Attendance					
>1/Week	0.89	0.01	0.88	0.91	0.00
1/Week	0.83	0.01	0.82	0.85	.
1-3/Month	0.75	0.01	0.72	0.78	.
A Few Times a Year	0.64	0.02	0.60	0.68	.
Never	0.54	0.03	0.48	0.61	.
Immigration Status					
Born in This Country	0.81	0.01	0.80	0.83	0.48
Born in Another Country	0.84	0.03	0.77	0.90	.
Religion					
Christianity	0.81	0.01	0.80	0.82	0.00
Islam	0.85	0.02	0.82	0.89	.
Hinduism	.	.	.	.	.
Buddhism	1.00	.	.	.	.
Judaism	1.00	.	.	.	.

Sikhism	.	.	.	.	.
Baha'i	1.00	.	.	.	.
Jainism	1.00	.	.	.	.
Shinto	.	.	.	.	.
Taoism	.	.	.	.	.
Confucianism	0.00	.	.	.	.
Primal, Animist, or Folk Religion	0.64	.	.	.	.
Spiritism	.	.	.	.	.
African-Derived	.	.	.	.	.
Chinese	.	.	.	.	.
Some Other Religion	0.57	0.14	-0.63	1.77	.
No Religion/Atheist/Agnostic	0.61	0.06	0.50	0.72	.
Race/Ethnicity					
Luhya	0.85	0.01	0.82	0.87	0.00
Luo	0.78	0.02	0.75	0.82	.
Kalenjin	0.81	0.02	0.78	0.85	.
Kamba	0.81	0.01	0.78	0.84	.
Kikuyu	0.77	0.02	0.74	0.80	.
Kisii	0.80	0.03	0.74	0.85	.
Maasai	0.86	0.03	0.80	0.91	.
Meru	0.79	0.03	0.74	0.85	.
Kenan Somali/Somali	0.81	0.04	0.74	0.89	.
Miji Kenda Tribes	0.87	0.02	0.82	0.91	.
Embu	0.85	0.02	0.80	0.90	.
Other	0.86	0.03	0.80	0.92	.

Table 12a: Nationally-Representative Descriptive Statistics of the Observed Sample (Mexico)

Variable	Proportion	Frequency
Age		
18-24	0.17	986
25-29	0.11	623
30-39	0.23	1312
40-49	0.18	1027
50-59	0.15	873
60-69	0.11	611
70-79	0.05	277
80 or Older	0.01	68
Missing	.	.
Gender		
Male	0.48	2755
Female	0.52	2997
Other	0.00	3
Missing	0.00	21
Marital Status		
Single/Never Been Married	0.25	1432
Married	0.36	2089
Separated	0.07	403
Divorced	0.04	230
Widowed	0.06	347
Domestic Partner	0.19	1109
Missing	0.03	166
Employment		
Employed for an Employer	0.33	1921
Self-Employed	0.19	1091
Retired	0.07	386
Student	0.04	247
Homemaker	0.22	1257
Unemployed and Looking for a Job	0.10	564
None of These/Other	0.03	169
Missing	0.02	141
Education		
Up to 8 Years	0.22	1291
9-15 Years	0.55	3180
16+ Years	0.23	1304
Missing	0.00	1
Service Attendance		
>1/Week	0.11	609
1/Week	0.22	1260
1-3/Month	0.12	676
A Few Times a Year	0.36	2054
Never	0.20	1134
Missing	0.01	43
Immigration Status		
Born in This Country	0.96	5517
Born in Another Country	0.02	108
Missing	0.03	151

Religion		
Christianity	0.84	4844
Islam	0.00	2
Hinduism	0.00	3
Buddhism	0.00	6
Judaism	0.00	7
Sikhism	.	.
Baha'i	0.00	1
Jainism	0.00	1
Shinto	0.00	2
Taoism	0.00	4
Confucianism	0.00	0
Primal, Animist, or Folk Religion	0.00	20
Spiritism	.	.
African-Derived	.	.
Chinese	.	.
Some Other Religion	0.01	41
No Religion/Atheist/Agnostic	0.13	770
Missing	0.01	75
Race/Ethnicity		
White	0.19	1116
Mestizo	0.48	2762
Indigenous	0.10	594
Black	0.02	108
Mulatto	0.01	63
Other	0.06	339
Missing	0.14	794

Table 12b: Variations Across Demographic Characteristics (Mexico)

Variable	Mean/Proportion	SE	LCI	UCI	Global p-value
Age					
18-24	0.39	0.02	0.35	0.42	0.00
25-29	0.46	0.02	0.41	0.51	.
30-39	0.49	0.02	0.46	0.53	.
40-49	0.59	0.02	0.55	0.63	.
50-59	0.66	0.02	0.62	0.70	.
60-69	0.68	0.03	0.62	0.73	.
70-79	0.70	0.04	0.62	0.78	.
80 or Older	0.80	0.08	0.64	0.97	.
Gender					
Male	0.53	0.01	0.50	0.56	0.14
Female	0.56	0.01	0.54	0.58	.
Other	0.39	0.16	-0.05	0.82	.
Marital Status					
Single/Never Been Married	0.45	0.02	0.41	0.48	0.00
Married	0.63	0.01	0.60	0.66	.
Separated	0.55	0.03	0.49	0.61	.
Divorced	0.58	0.04	0.50	0.66	.
Widowed	0.72	0.03	0.65	0.79	.
Domestic Partner	0.45	0.02	0.42	0.49	.
Employment					
Employed for an Employer	0.51	0.01	0.48	0.54	0.00
Self-Employed	0.58	0.02	0.54	0.62	.
Retired	0.69	0.03	0.63	0.76	.
Student	0.27	0.04	0.19	0.34	.
Homemaker	0.61	0.02	0.58	0.64	.
Unemployed and Looking for a Job	0.51	0.02	0.46	0.56	.
None of These/Other	0.52	0.05	0.42	0.61	.
Education					
Up to 8 Years	0.67	0.02	0.63	0.70	0.00
9-15 Years	0.52	0.01	0.50	0.55	.
16+ Years	0.48	0.02	0.45	0.52	.
Service Attendance					
>1/Week	0.84	0.02	0.80	0.87	0.00
1/Week	0.76	0.02	0.73	0.79	.
1-3/Month	0.65	0.02	0.60	0.69	.
A Few Times a Year	0.43	0.01	0.40	0.46	.
Never	0.31	0.02	0.27	0.34	.
Immigration Status					
Born in This Country	0.55	0.01	0.53	0.57	0.00
Born in Another Country	0.37	0.06	0.26	0.49	.
Religion					
Christianity	0.59	0.01	0.58	0.61	0.00
Islam	0.29	.	.	.	.
Hinduism	0.55	0.29	-0.71	1.80	.
Buddhism	0.36	0.23	-0.30	1.03	.

Judaism	0.76	0.17	0.31	1.21	.
Sikhism	.	.	.	.	.
Baha'i	.	.	.	.	.
Jainism	0.00	.	.	.	.
Shinto	0.00	.	.	.	.
Taoism	0.94	0.12	-18.25	20.13	.
Confucianism	0.00	.	.	.	.
Primal, Animist, or Folk Religion	0.57	0.13	0.27	0.86	.
Spiritism	.	.	.	.	.
African-Derived	.	.	.	.	.
Chinese	.	.	.	.	.
Some Other Religion	0.54	0.09	0.35	0.72	.
No Religion/Atheist/Agnostic	0.25	0.02	0.21	0.29	.
Race/Ethnicity					
White	0.58	0.02	0.54	0.61	0.16
Mestizo	0.53	0.01	0.50	0.55	.
Indigenous	0.56	0.03	0.51	0.61	.
Black	0.55	0.06	0.44	0.66	.
Mulatto	0.52	0.07	0.36	0.67	.
Other	0.60	0.03	0.53	0.66	.

Table 13a: Nationally-Representative Descriptive Statistics of the Observed Sample (Nigeria)

Variable	Proportion	Frequency
Age		
18-24	0.22	1533
25-29	0.17	1193
30-39	0.28	1943
40-49	0.16	1059
50-59	0.09	619
60-69	0.04	296
70-79	0.02	133
80 or Older	0.01	50
Missing	.	.
Gender		
Male	0.49	3371
Female	0.51	3456
Other	0.00	0
Missing	.	.
Marital Status		
Single/Never Been Married	0.34	2289
Married	0.60	4065
Separated	0.02	117
Divorced	0.01	71
Widowed	0.03	231
Domestic Partner	0.00	12
Missing	0.01	42
Employment		
Employed for an Employer	0.10	699
Self-Employed	0.57	3898
Retired	0.03	178
Student	0.10	650
Homemaker	0.07	499
Unemployed and Looking for a Job	0.10	684
None of These/Other	0.03	211
Missing	0.00	8
Education		
Up to 8 Years	0.38	2575
9-15 Years	0.60	4120
16+ Years	0.02	130
Missing	0.00	2
Service Attendance		
>1/Week	0.59	4049
1/Week	0.28	1895
1-3/Month	0.08	531
A Few Times a Year	0.04	254
Never	0.01	77
Missing	0.00	20
Immigration Status		
Born in This Country	0.99	6779
Born in Another Country	0.01	47
Missing	0.00	1

Religion		
Christianity	0.51	3476
Islam	0.48	3302
Hinduism	.	.
Buddhism	.	.
Judaism	.	.
Sikhism	.	.
Baha'i	.	.
Jainism	.	.
Shinto	0.00	0
Taoism	.	.
Confucianism	0.00	0
Primal, Animist, or Folk Religion	0.00	24
Spiritism	.	.
African-Derived	.	.
Chinese	.	.
Some Other Religion	0.00	1
No Religion/Atheist/Agnostic	0.00	15
Missing	0.00	9
Race/Ethnicity		
Hausa	0.34	2342
Yoruba	0.18	1230
Igbo (Ibo)	0.16	1112
Edo	0.02	116
Urhobo	0.01	38
Fulani	0.04	266
Kanuri	0.00	31
Tiv	0.03	198
Efik	0.01	48
Ijaw	0.02	110
Igala	0.01	77
Ibibio	0.03	180
Idoma	0.01	61
Other	0.15	1014
Missing	0.00	4

Table 13b: Variations Across Demographic Characteristics (Nigeria)

Variable	Mean/Proportion	SE	LCI	UCI	Global p-value
Age					
18-24	0.89	0.01	0.87	0.91	0.17
25-29	0.89	0.01	0.86	0.91	.
30-39	0.89	0.01	0.87	0.91	.
40-49	0.90	0.01	0.87	0.92	.
50-59	0.92	0.02	0.88	0.96	.
60-69	0.91	0.03	0.86	0.97	.
70-79	0.83	0.04	0.74	0.91	.
80 or Older	0.59	0.10	0.25	0.93	.
Gender					
Male	0.88	0.01	0.87	0.90	0.00
Female	0.90	0.01	0.88	0.91	.
Other	1.00	.	.	.	.
Marital Status					
Single/Never Been Married	0.88	0.01	0.86	0.90	0.00
Married	0.90	0.01	0.89	0.92	.
Separated	0.74	0.06	0.62	0.85	.
Divorced	0.90	0.03	0.83	0.97	.
Widowed	0.88	0.04	0.80	0.96	.
Domestic Partner	0.42	0.04	0.09	0.76	.
Employment					
Employed for an Employer	0.86	0.01	0.83	0.89	0.05
Self-Employed	0.90	0.01	0.89	0.91	.
Retired	0.86	0.05	0.76	0.96	.
Student	0.91	0.01	0.89	0.93	.
Homemaker	0.90	0.02	0.85	0.95	.
Unemployed and Looking for a Job	0.86	0.02	0.82	0.90	.
None of These/Other	0.87	0.05	0.77	0.98	.
Education					
Up to 8 Years	0.90	0.01	0.88	0.93	0.10
9-15 Years	0.88	0.01	0.87	0.89	.
16+ Years	0.85	0.02	0.81	0.90	.
Service Attendance					
>1/Week	0.93	0.01	0.92	0.94	0.00
1/Week	0.87	0.01	0.85	0.89	.
1-3/Month	0.78	0.02	0.73	0.82	.
A Few Times a Year	0.77	0.04	0.70	0.84	.
Never	0.71	0.08	0.55	0.87	.
Immigration Status					
Born in This Country	0.89	0.01	0.88	0.90	0.69
Born in Another Country	0.86	0.07	0.71	1.01	.
Religion					
Christianity	0.86	0.01	0.84	0.87	0.00
Islam	0.93	0.01	0.92	0.94	.
Hinduism	.	.	.	.	.
Buddhism	.	.	.	.	.
Judaism	.	.	.	.	.

Sikhism	.	.	.	.	.
Baha'i	.	.	.	.	.
Jainism	.	.	.	.	.
Shinto	1.00	.	.	.	.
Taoism	.	.	.	.	.
Confucianism	1.00	.	.	.	.
Primal, Animist, or Folk Religion	0.88	0.11	0.58	1.18	.
Spiritism	.	.	.	.	.
African-Derived	.	.	.	.	.
Chinese	.	.	.	.	.
Some Other Religion	1.00	.	.	.	.
No Religion/Atheist/Agnostic	0.72	0.14	-0.46	1.89	.
Race/Ethnicity					
Hausa	0.94	0.01	0.92	0.95	0.00
Yoruba	0.85	0.02	0.82	0.89	.
Igbo (Ibo)	0.85	0.01	0.82	0.88	.
Edo	0.85	0.03	0.78	0.92	.
Urhobo	0.93	0.02	0.88	0.98	.
Fulani	0.92	0.03	0.86	0.98	.
Kanuri	0.98	0.01	0.95	1.00	.
Tiv	0.88	0.05	0.79	0.98	.
Efik	0.88	0.03	0.82	0.93	.
Ijaw	0.77	0.06	0.63	0.92	.
Igala	0.89	0.03	0.83	0.95	.
Ibibio	0.88	0.03	0.81	0.96	.
Idoma	0.78	0.07	0.64	0.93	.
Other	0.89	0.01	0.86	0.91	.

Table 14a: Nationally-Representative Descriptive Statistics of the Observed Sample (Philippines)

Variable	Proportion	Frequency
Age		
18-24	0.20	1073
25-29	0.13	695
30-39	0.22	1160
40-49	0.18	972
50-59	0.14	732
60-69	0.09	495
70-79	0.03	143
80 or Older	0.00	23
Missing	.	.
Gender		
Male	0.50	2625
Female	0.50	2643
Other	0.00	13
Missing	0.00	11
Marital Status		
Single/Never Been Married	0.23	1206
Married	0.45	2385
Separated	0.05	249
Divorced	0.00	9
Widowed	0.05	274
Domestic Partner	0.22	1152
Missing	0.00	16
Employment		
Employed for an Employer	0.26	1350
Self-Employed	0.26	1379
Retired	0.03	158
Student	0.11	585
Homemaker	0.20	1049
Unemployed and Looking for a Job	0.12	658
None of These/Other	0.02	113
Missing	.	.
Education		
Up to 8 Years	0.22	1188
9-15 Years	0.70	3722
16+ Years	0.07	381
Missing	0.00	1
Service Attendance		
>1/Week	0.16	844
1/Week	0.36	1929
1-3/Month	0.26	1374
A Few Times a Year	0.18	929
Never	0.04	210
Missing	0.00	6
Immigration Status		
Born in This Country	1.00	5284
Born in Another Country	0.00	8
Missing	.	.

Religion		
Christianity	0.93	4914
Islam	0.06	297
Hinduism	.	.
Buddhism	0.00	4
Judaism	0.00	4
Sikhism	.	.
Baha'i	0.00	1
Jainism	.	.
Shinto	.	.
Taoism	.	.
Confucianism	.	.
Primal, Animist, or Folk Religion	0.00	5
Spiritism	.	.
African-Derived	.	.
Chinese	.	.
Some Other Religion	0.01	35
No Religion/Atheist/Agnostic	0.00	23
Missing	0.00	9
Race/Ethnicity		
Tagalog	0.32	1691
Cebuana	0.12	656
Ilocano/Ilokano	0.08	429
Visayan/Bisaya	0.14	739
Ilonggo/Hiligaynon	0.08	428
Bicolano/Bikolano	0.06	300
Waray	0.04	216
Tausug	0.02	94
Maranao	0.01	39
Maguindanaoan	0.02	84
Chinese-Filipino	0.00	3
Kapampangan	0.02	107
Pangasinese	0.02	107
Zamboangueno	0.01	51
Malay	.	.
Masbateno	0.01	54
Aeta	0.00	1
Igorot	0.01	42
Mangyan	0.00	2
Badjao	0.00	2
Other	0.05	244
Missing	0.00	3

Table 14b: Variations Across Demographic Characteristics (Philippines)

Variable	Mean/Proportion	SE	LCI	UCI	Global p-value
Age					
18-24	0.67	0.02	0.63	0.71	0.00
25-29	0.77	0.02	0.72	0.81	.
30-39	0.77	0.01	0.74	0.80	.
40-49	0.77	0.01	0.74	0.80	.
50-59	0.73	0.02	0.69	0.77	.
60-69	0.74	0.03	0.68	0.79	.
70-79	0.74	0.05	0.65	0.83	.
80 or Older	0.89	0.03	0.79	1.00	.
Gender					
Male	0.72	0.01	0.70	0.75	0.07
Female	0.76	0.01	0.74	0.77	.
Other	0.81	0.06	0.66	0.95	.
Marital Status					
Single/Never Been Married	0.68	0.02	0.65	0.72	0.00
Married	0.77	0.01	0.75	0.79	.
Separated	0.65	0.04	0.58	0.72	.
Divorced	0.49	0.12	-0.52	1.51	.
Widowed	0.75	0.03	0.69	0.81	.
Domestic Partner	0.75	0.01	0.72	0.78	.
Employment					
Employed for an Employer	0.76	0.02	0.73	0.80	0.05
Self-Employed	0.75	0.01	0.72	0.78	.
Retired	0.71	0.05	0.61	0.81	.
Student	0.67	0.03	0.62	0.72	.
Homemaker	0.74	0.01	0.72	0.77	.
Unemployed and Looking for a Job	0.73	0.02	0.68	0.77	.
None of These/Other	0.77	0.05	0.68	0.86	.
Education					
Up to 8 Years	0.71	0.02	0.67	0.74	0.00
9-15 Years	0.74	0.01	0.72	0.76	.
16+ Years	0.86	0.02	0.82	0.90	.
Service Attendance					
>1/Week	0.82	0.02	0.78	0.86	0.00
1/Week	0.78	0.01	0.76	0.81	.
1-3/Month	0.71	0.02	0.68	0.74	.
A Few Times a Year	0.66	0.02	0.62	0.70	.
Never	0.57	0.04	0.48	0.65	.
Immigration Status					
Born in This Country	0.74	0.01	0.72	0.76	0.49
Born in Another Country	0.61	0.21	-0.29	1.51	.
Religion					
Christianity	0.74	0.01	0.72	0.75	0.00
Islam	0.79	0.04	0.71	0.87	.
Hinduism	.	.	.	.	.
Buddhism	0.80	0.02	-2.15	3.76	.

Judaism	1.00	.	.	.	.
Sikhism	.	.	.	.	.
Baha'i	1.00	.	.	.	.
Jainism	.	.	.	.	.
Shinto	.	.	.	.	.
Taoism	.	.	.	.	.
Confucianism	.	.	.	.	.
Primal, Animist, or Folk Religion	0.23	0.16	-1.16	1.62	.
Spiritism	.	.	.	.	.
African-Derived	.	.	.	.	.
Chinese	.	.	.	.	.
Some Other Religion	0.74	0.06	0.63	0.86	.
No Religion/Atheist/Agnostic	0.50	0.09	0.29	0.72	.
Race/Ethnicity					
Tagalog	0.72	0.01	0.69	0.75	0.00
Cebuana	0.75	0.02	0.70	0.80	.
Ilocano/Ilokano	0.75	0.03	0.69	0.80	.
Visayan/Bisaya	0.73	0.02	0.69	0.77	.
Ilonggo/Hiligaynon	0.80	0.02	0.75	0.84	.
Bicolano/Bikolano	0.69	0.04	0.62	0.76	.
Waray	0.73	0.04	0.65	0.81	.
Tausug	0.88	0.02	0.84	0.93	.
Maranao	0.85	0.00	0.55	1.14	.
Maguindanaoan	0.68	0.06	0.54	0.82	.
Chinese-Filipino	0.87	0.14	-21.56	23.31	.
Kapampangan	0.70	0.09	0.51	0.88	.
Pangasinese	0.78	0.04	0.69	0.87	.
Zamboangueno	0.68	0.07	0.49	0.87	.
Malay	.	.	.	.	.
Masbateno	0.82	0.08	0.59	1.05	.
Aeta	1.00	.	.	.	.
Igorot	0.86	0.03	0.77	0.96	.
Mangyan	0.32	.	.	.	.
Badjao	0.85	0.01	-0.48	2.18	.
Other	0.77	0.03	0.70	0.84	.

Table 15a: Nationally-Representative Descriptive Statistics of the Observed Sample (Poland)

Variable	Proportion	Frequency
Age		
18-24	0.09	955
25-29	0.07	761
30-39	0.21	2159
40-49	0.19	1956
50-59	0.16	1670
60-69	0.18	1909
70-79	0.08	833
80 or Older	0.01	145
Missing	0.00	1
Gender		
Male	0.48	4974
Female	0.52	5387
Other	0.00	3
Missing	0.00	26
Marital Status		
Single/Never Been Married	0.17	1811
Married	0.58	6065
Separated	0.01	111
Divorced	0.05	529
Widowed	0.10	990
Domestic Partner	0.05	504
Missing	0.04	379
Employment		
Employed for an Employer	0.56	5837
Self-Employed	0.07	686
Retired	0.23	2434
Student	0.05	515
Homemaker	0.03	338
Unemployed and Looking for a Job	0.03	284
None of These/Other	0.02	169
Missing	0.01	126
Education		
Up to 8 Years	0.12	1238
9-15 Years	0.59	6130
16+ Years	0.29	3020
Missing	0.00	1
Service Attendance		
>1/Week	0.03	305
1/Week	0.31	3263
1-3/Month	0.20	2081
A Few Times a Year	0.29	3064
Never	0.15	1597
Missing	0.01	78
Immigration Status		
Born in This Country	0.99	10258
Born in Another Country	0.01	108
Missing	0.00	23

Religion		
Christianity	0.90	9378
Islam	0.00	2
Hinduism	.	.
Buddhism	0.00	2
Judaism	.	.
Sikhism	0.00	1
Baha'i	.	.
Jainism	0.00	3
Shinto	0.00	1
Taoism	.	.
Confucianism	.	.
Primal, Animist, or Folk Religion	0.00	11
Spiritism	.	.
African-Derived	.	.
Chinese	.	.
Some Other Religion	.	.
No Religion/Atheist/Agnostic	0.09	942
Missing	0.00	50
Race/Ethnicity		
Polish	0.99	10309
German	0.00	4
Belarussian	0.00	2
Ukranian	0.00	38
Roma	.	.
Russian	.	.
Ethnic Jewish	.	.
Lemko	.	.
Silesia	0.00	14
Kashubians	0.00	3
Other	0.00	4
Missing	0.00	14

Table 15b: Variations Across Demographic Characteristics (Poland)

Variable	Mean/Proportion	SE	LCI	UCI	Global p-value
Age					
18-24	0.23	0.02	0.18	0.28	0.00
25-29	0.28	0.02	0.24	0.32	.
30-39	0.36	0.02	0.32	0.39	.
40-49	0.40	0.02	0.37	0.44	.
50-59	0.47	0.02	0.43	0.51	.
60-69	0.59	0.02	0.54	0.64	.
70-79	0.62	0.03	0.55	0.69	.
80 or Older	0.76	0.05	0.65	0.87	.
Gender					
Male	0.37	0.02	0.34	0.40	0.00
Female	0.50	0.01	0.47	0.53	.
Other	0.13	.	.	.	.
Marital Status					
Single/Never Been Married	0.27	0.02	0.24	0.31	0.00
Married	0.48	0.02	0.45	0.51	.
Separated	0.41	0.07	0.28	0.55	.
Divorced	0.38	0.03	0.32	0.45	.
Widowed	0.62	0.03	0.56	0.67	.
Domestic Partner	0.22	0.02	0.18	0.27	.
Employment					
Employed for an Employer	0.39	0.01	0.36	0.42	0.00
Self-Employed	0.33	0.03	0.28	0.38	.
Retired	0.64	0.02	0.60	0.68	.
Student	0.22	0.03	0.16	0.28	.
Homemaker	0.44	0.04	0.35	0.53	.
Unemployed and Looking for a Job	0.31	0.05	0.21	0.42	.
None of These/Other	0.40	0.05	0.29	0.51	.
Education					
Up to 8 Years	0.40	0.04	0.33	0.48	0.00
9-15 Years	0.46	0.01	0.43	0.49	.
16+ Years	0.40	0.02	0.37	0.43	.
Service Attendance					
>1/Week	0.90	0.02	0.85	0.95	0.00
1/Week	0.77	0.02	0.74	0.80	.
1-3/Month	0.42	0.02	0.38	0.47	.
A Few Times a Year	0.22	0.02	0.19	0.26	.
Never	0.10	0.01	0.07	0.12	.
Immigration Status					
Born in This Country	0.44	0.01	0.41	0.46	0.09
Born in Another Country	0.31	0.06	0.19	0.44	.
Religion					
Christianity	0.46	0.01	0.44	0.49	0.00
Islam	0.34	.	.	.	.
Hinduism	.	.	.	.	.
Buddhism	0.00	.	.	.	.

Judaism	.	.	.	.	.
Sikhism	0.00	.	.	.	.
Baha'i	.	.	.	.	.
Jainism	0.00	.	.	.	.
Shinto	1.00	.	.	.	.
Taoism	.	.	.	.	.
Confucianism	.	.	.	.	.
Primal, Animist, or Folk Religion	0.25	0.10	-15.65	16.15	.
Spiritism	.	.	.	.	.
African-Derived	.	.	.	.	.
Chinese	.	.	.	.	.
Some Other Religion	.	.	.	.	.
No Religion/Atheist/Agnostic	0.17	0.02	0.12	0.21	.
Race/Ethnicity					
Polish	0.44	0.01	0.41	0.46	0.00
German	0.18	.	.	.	.
Belarussian	.	.	.	.	.
Ukranian	0.20	0.06	0.03	0.36	.
Roma	.	.	.	.	.
Russian	.	.	.	.	.
Ethnic Jewish	.	.	.	.	.
Lemko	.	.	.	.	.
Silesia	0.56	0.10	0.11	1.01	.
Kashubians	0.00	.	.	.	.
Other	0.00	.	.	.	.

Table 16a: Nationally-Representative Descriptive Statistics of the Observed Sample (South Africa)

Variable	Proportion	Frequency
Age		
18-24	0.17	461
25-29	0.14	364
30-39	0.25	655
40-49	0.20	522
50-59	0.12	309
60-69	0.07	195
70-79	0.05	120
80 or Older	0.01	17
Missing	0.00	9
Gender		
Male	0.49	1288
Female	0.51	1356
Other	0.00	2
Missing	0.00	4
Marital Status		
Single/Never Been Married	0.59	1561
Married	0.20	539
Separated	0.03	76
Divorced	0.02	51
Widowed	0.05	133
Domestic Partner	0.10	264
Missing	0.01	28
Employment		
Employed for an Employer	0.21	569
Self-Employed	0.16	412
Retired	0.09	243
Student	0.08	204
Homemaker	0.05	137
Unemployed and Looking for a Job	0.38	1008
None of These/Other	0.03	74
Missing	0.00	3
Education		
Up to 8 Years	0.25	668
9-15 Years	0.68	1796
16+ Years	0.07	183
Missing	0.00	4
Service Attendance		
>1/Week	0.16	414
1/Week	0.34	891
1-3/Month	0.22	574
A Few Times a Year	0.16	431
Never	0.13	334
Missing	0.00	7
Immigration Status		
Born in This Country	0.95	2511
Born in Another Country	0.05	139
Missing	0.00	1

Religion		
Christianity	0.82	2163
Islam	0.02	62
Hinduism	0.00	1
Buddhism	0.00	12
Judaism	.	.
Sikhism	.	.
Baha'i	.	.
Jainism	0.00	2
Shinto	0.00	2
Taoism	0.00	1
Confucianism	.	.
Primal, Animist, or Folk Religion	0.05	127
Spiritism	.	.
African-Derived	.	.
Chinese	.	.
Some Other Religion	0.00	5
No Religion/Atheist/Agnostic	0.10	253
Missing	0.01	23
Race/Ethnicity		
Black	0.90	2381
Asian/Indian	0.00	6
Colored	0.10	252
White	0.00	8
Other	0.00	0
Missing	0.00	3

Table 16b: Variations Across Demographic Characteristics (South Africa)

Variable	Mean/Proportion	SE	LCI	UCI	Global p-value
Age					
18-24	0.72	0.02	0.67	0.77	0.00
25-29	0.75	0.03	0.70	0.80	.
30-39	0.75	0.02	0.71	0.79	.
40-49	0.77	0.02	0.73	0.82	.
50-59	0.89	0.03	0.83	0.94	.
60-69	0.89	0.03	0.82	0.96	.
70-79	0.91	0.06	0.79	1.03	.
80 or Older	0.71	0.23	-1.27	2.69	.
Gender					
Male	0.75	0.02	0.72	0.78	0.00
Female	0.81	0.01	0.79	0.84	.
Other	1.00	.	.	.	.
Marital Status					
Single/Never Been Married	0.76	0.01	0.73	0.78	0.00
Married	0.86	0.02	0.82	0.90	.
Separated	0.67	0.08	0.51	0.84	.
Divorced	0.73	0.10	0.52	0.93	.
Widowed	0.90	0.05	0.81	1.00	.
Domestic Partner	0.76	0.03	0.70	0.82	.
Employment					
Employed for an Employer	0.75	0.02	0.70	0.80	0.00
Self-Employed	0.75	0.03	0.70	0.80	.
Retired	0.90	0.03	0.84	0.97	.
Student	0.70	0.04	0.61	0.78	.
Homemaker	0.91	0.03	0.84	0.97	.
Unemployed and Looking for a Job	0.79	0.02	0.76	0.82	.
None of These/Other	0.80	0.08	0.64	0.95	.
Education					
Up to 8 Years	0.86	0.02	0.81	0.90	0.00
9-15 Years	0.76	0.01	0.74	0.79	.
16+ Years	0.71	0.04	0.63	0.78	.
Service Attendance					
>1/Week	0.86	0.02	0.81	0.90	0.00
1/Week	0.86	0.01	0.83	0.88	.
1-3/Month	0.79	0.02	0.74	0.84	.
A Few Times a Year	0.68	0.03	0.63	0.74	.
Never	0.61	0.03	0.55	0.68	.
Immigration Status					
Born in This Country	0.78	0.01	0.76	0.80	0.92
Born in Another Country	0.79	0.06	0.68	0.90	.
Religion					
Christianity	0.79	0.01	0.77	0.82	0.00
Islam	0.83	0.08	0.66	0.99	.
Hinduism	1.00	.	.	.	.
Buddhism	0.60	0.16	0.16	1.04	.

Judaism	.	.	.	.	.
Sikhism	.	.	.	.	.
Baha'i	.	.	.	.	.
Jainism	1.00	.	.	.	.
Shinto	1.00	.	.	.	.
Taoism	1.00	.	.	.	.
Confucianism	.	.	.	.	.
Primal, Animist, or Folk Religion	0.75	0.05	0.65	0.86	.
Spiritism	.	.	.	.	.
African-Derived	.	.	.	.	.
Chinese	.	.	.	.	.
Some Other Religion	0.49	0.08	-2.10e+34	2.10e+34	.
No Religion/Atheist/Agnostic	0.71	0.03	0.65	0.77	.
Race/Ethnicity					
Black	0.78	0.01	0.76	0.80	0.00
Asian/Indian	1.00	.	.	.	.
Colored	0.82	0.03	0.75	0.89	.
White	0.81	0.10	-0.06	1.69	.
Other	1.00	.	.	.	.

Table 17a: Nationally-Representative Descriptive Statistics of the Observed Sample (Spain)

Variable	Proportion	Frequency
Age		
18-24	0.09	594
25-29	0.07	450
30-39	0.18	1111
40-49	0.22	1396
50-59	0.20	1252
60-69	0.16	977
70-79	0.07	467
80 or Older	0.01	43
Missing	.	.
Gender		
Male	0.50	3142
Female	0.50	3119
Other	0.00	6
Missing	0.00	22
Marital Status		
Single/Never Been Married	0.28	1742
Married	0.47	2947
Separated	0.04	237
Divorced	0.08	518
Widowed	0.03	189
Domestic Partner	0.09	589
Missing	0.01	67
Employment		
Employed for an Employer	0.45	2862
Self-Employed	0.09	576
Retired	0.20	1278
Student	0.07	448
Homemaker	0.05	345
Unemployed and Looking for a Job	0.10	646
None of These/Other	0.02	123
Missing	0.00	11
Education		
Up to 8 Years	0.13	802
9-15 Years	0.66	4145
16+ Years	0.21	1341
Missing	0.00	2
Service Attendance		
>1/Week	0.05	317
1/Week	0.11	662
1-3/Month	0.07	437
A Few Times a Year	0.31	1972
Never	0.46	2875
Missing	0.00	27
Immigration Status		
Born in This Country	0.87	5479
Born in Another Country	0.13	788
Missing	0.00	23

Religion		
Christianity	0.65	4074
Islam	0.02	135
Hinduism	0.00	7
Buddhism	0.01	36
Judaism	0.00	4
Sikhism	0.00	3
Baha'i	0.00	2
Jainism	0.00	1
Shinto	.	.
Taoism	0.00	5
Confucianism	0.00	3
Primal, Animist, or Folk Religion	0.00	7
Spiritism	.	.
African-Derived	.	.
Chinese	.	.
Some Other Religion	0.00	27
No Religion/Atheist/Agnostic	0.31	1932
Missing	0.01	55
Race/Ethnicity		
No Data	.	.

Table 17b: Variations Across Demographic Characteristics (Spain)

Variable	Mean/Proportion	SE	LCI	UCI	Global p-value
Age					
18-24	0.23	0.02	0.19	0.28	0.00
25-29	0.25	0.02	0.21	0.30	.
30-39	0.23	0.01	0.21	0.26	.
40-49	0.24	0.01	0.21	0.27	.
50-59	0.28	0.02	0.24	0.31	.
60-69	0.29	0.03	0.24	0.34	.
70-79	0.39	0.04	0.30	0.47	.
80 or Older	0.59	0.12	0.34	0.84	.
Gender					
Male	0.28	0.01	0.26	0.30	0.11
Female	0.26	0.01	0.23	0.28	.
Other	0.55	0.15	0.19	0.91	.
Marital Status					
Single/Never Been Married	0.24	0.01	0.21	0.26	0.00
Married	0.29	0.01	0.27	0.31	.
Separated	0.25	0.03	0.18	0.31	.
Divorced	0.23	0.03	0.18	0.28	.
Widowed	0.42	0.06	0.30	0.53	.
Domestic Partner	0.24	0.02	0.19	0.28	.
Employment					
Employed for an Employer	0.23	0.01	0.21	0.25	0.00
Self-Employed	0.29	0.02	0.25	0.33	.
Retired	0.33	0.02	0.29	0.38	.
Student	0.23	0.03	0.18	0.28	.
Homemaker	0.37	0.04	0.29	0.45	.
Unemployed and Looking for a Job	0.27	0.02	0.23	0.30	.
None of These/Other	0.24	0.04	0.15	0.32	.
Education					
Up to 8 Years	0.33	0.03	0.27	0.39	0.02
9-15 Years	0.26	0.01	0.25	0.28	.
16+ Years	0.24	0.01	0.21	0.27	.
Service Attendance					
>1/Week	0.67	0.03	0.61	0.74	0.00
1/Week	0.61	0.03	0.56	0.66	.
1-3/Month	0.42	0.03	0.36	0.48	.
A Few Times a Year	0.27	0.01	0.25	0.30	.
Never	0.11	0.01	0.10	0.13	.
Immigration Status					
Born in This Country	0.25	0.01	0.24	0.27	0.00
Born in Another Country	0.36	0.02	0.33	0.40	.
Religion					
Christianity	0.35	0.01	0.33	0.37	0.00
Islam	0.45	0.05	0.35	0.56	.
Hinduism	0.54	0.14	-0.05	1.12	.
Buddhism	0.36	0.10	0.15	0.57	.
Judaism	0.11	0.13	-0.43	0.65	.

Sikhism	0.35	0.05	-7.07	7.78	.
Baha'i	0.40	.	.	.	.
Jainism	0.00	.	.	.	.
Shinto	.	.	.	.	.
Taoism	0.00	.	.	.	.
Confucianism	0.49	0.38	-2.76	3.74	.
Primal, Animist, or Folk Religion	0.44	0.15	0.11	0.77	.
Spiritism	.	.	.	.	.
African-Derived	.	.	.	.	.
Chinese	.	.	.	.	.
Some Other Religion	0.24	0.08	0.08	0.40	.
No Religion/Atheist/Agnostic	0.08	0.01	0.06	0.10	.
Race/Ethnicity					
No Data	.	.	.	.	.

Table 18a: Nationally-Representative Descriptive Statistics of the Observed Sample (Sweden)

Variable	Proportion	Frequency
Age		
18-24	0.10	1515
25-29	0.09	1399
30-39	0.16	2398
40-49	0.15	2221
50-59	0.17	2493
60-69	0.14	2168
70-79	0.15	2253
80 or Older	0.04	621
Missing	.	.
Gender		
Male	0.50	7536
Female	0.50	7493
Other	0.00	27
Missing	0.00	12
Marital Status		
Single/Never Been Married	0.26	3854
Married	0.43	6408
Separated	0.03	426
Divorced	0.05	801
Widowed	0.03	433
Domestic Partner	0.20	3073
Missing	0.00	72
Employment		
Employed for an Employer	0.52	7907
Self-Employed	0.08	1243
Retired	0.25	3832
Student	0.09	1332
Homemaker	0.00	75
Unemployed and Looking for a Job	0.02	324
None of These/Other	0.02	337
Missing	0.00	18
Education		
Up to 8 Years	0.02	252
9-15 Years	0.72	10790
16+ Years	0.27	4026
Missing	.	.
Service Attendance		
>1/Week	0.02	236
1/Week	0.03	434
1-3/Month	0.03	486
A Few Times a Year	0.26	3950
Never	0.66	9918
Missing	0.00	45
Immigration Status		
Born in This Country	0.92	13922
Born in Another Country	0.07	1052
Missing	0.01	94

Religion		
Christianity	0.55	8346
Islam	0.03	470
Hinduism	0.00	22
Buddhism	0.01	110
Judaism	0.00	54
Sikhism	0.00	4
Baha'i	0.00	6
Jainism	.	.
Shinto	0.00	0
Taoism	0.00	4
Confucianism	.	.
Primal, Animist, or Folk Religion	0.01	83
Spiritism	.	.
African-Derived	.	.
Chinese	.	.
Some Other Religion	0.01	198
No Religion/Atheist/Agnostic	0.38	5697
Missing	0.00	74
Race/Ethnicity		
No Data	.	.

Table 18b: Variations Across Demographic Characteristics (Sweden)

Variable	Mean/Proportion	SE	LCI	UCI	Global p-value
Age					
18-24	0.15	0.01	0.13	0.18	0.00
25-29	0.12	0.01	0.10	0.14	.
30-39	0.10	0.01	0.08	0.11	.
40-49	0.12	0.01	0.10	0.14	.
50-59	0.12	0.01	0.10	0.14	.
60-69	0.13	0.01	0.11	0.15	.
70-79	0.14	0.01	0.12	0.16	.
80 or Older	0.19	0.02	0.15	0.23	.
Gender					
Male	0.11	0.00	0.10	0.12	0.00
Female	0.14	0.01	0.13	0.15	.
Other	0.12	0.09	-0.06	0.31	.
Marital Status					
Single/Never Been Married	0.13	0.01	0.11	0.14	0.00
Married	0.15	0.01	0.14	0.16	.
Separated	0.13	0.02	0.08	0.17	.
Divorced	0.14	0.01	0.11	0.17	.
Widowed	0.16	0.02	0.12	0.20	.
Domestic Partner	0.07	0.01	0.06	0.08	.
Employment					
Employed for an Employer	0.11	0.00	0.10	0.12	0.00
Self-Employed	0.14	0.02	0.11	0.17	.
Retired	0.15	0.01	0.14	0.16	.
Student	0.16	0.01	0.13	0.19	.
Homemaker	0.22	0.06	0.10	0.34	.
Unemployed and Looking for a Job	0.11	0.02	0.07	0.15	.
None of These/Other	0.13	0.02	0.08	0.17	.
Education					
Up to 8 Years	0.16	0.03	0.10	0.22	0.03
9-15 Years	0.12	0.00	0.11	0.13	.
16+ Years	0.14	0.01	0.13	0.15	.
Service Attendance					
>1/Week	0.89	0.03	0.83	0.94	0.00
1/Week	0.76	0.03	0.71	0.81	.
1-3/Month	0.56	0.03	0.51	0.62	.
A Few Times a Year	0.16	0.01	0.14	0.17	.
Never	0.05	0.00	0.04	0.05	.
Immigration Status					
Born in This Country	0.12	0.00	0.11	0.12	0.00
Born in Another Country	0.26	0.02	0.22	0.29	.
Religion					
Christianity	0.18	0.01	0.17	0.19	0.00
Islam	0.48	0.03	0.41	0.55	.
Hinduism	0.36	0.17	-0.04	0.76	.
Buddhism	0.24	0.06	0.11	0.37	.

Judaism	0.12	0.07	-0.03	0.28	.
Sikhism	0.55	0.49	-80.77	81.88	.
Baha'i	0.44	0.37	-2.77	3.65	.
Jainism	.	.	.	.	.
Shinto	0.00	.	.	.	.
Taoism	0.70	0.42	-68.10	69.50	.
Confucianism	.	.	.	.	.
Primal, Animist, or Folk Religion	0.15	0.06	0.03	0.28	.
Spiritism	.	.	.	.	.
African-Derived	.	.	.	.	.
Chinese	.	.	.	.	.
Some Other Religion	0.20	0.04	0.11	0.28	.
No Religion/Atheist/Agnostic	0.02	0.00	0.01	0.02	.
Race/Ethnicity					
No Data	.	.	.	.	.

Table 19a: Nationally-Representative Descriptive Statistics of the Observed Sample (Tanzania)

Variable	Proportion	Frequency
Age		
18-24	0.25	2284
25-29	0.15	1349
30-39	0.23	2060
40-49	0.17	1503
50-59	0.10	912
60-69	0.06	575
70-79	0.03	297
80 or Older	0.01	93
Missing	0.00	2
Gender		
Male	0.47	4299
Female	0.53	4776
Other	.	.
Missing	.	.
Marital Status		
Single/Never Been Married	0.25	2260
Married	0.61	5577
Separated	0.04	404
Divorced	0.01	103
Widowed	0.05	450
Domestic Partner	0.03	275
Missing	0.00	7
Employment		
Employed for an Employer	0.06	513
Self-Employed	0.51	4625
Retired	0.02	139
Student	0.04	319
Homemaker	0.20	1796
Unemployed and Looking for a Job	0.16	1491
None of These/Other	0.02	186
Missing	0.00	6
Education		
Up to 8 Years	0.74	6699
9-15 Years	0.25	2252
16+ Years	0.01	122
Missing	0.00	2
Service Attendance		
>1/Week	0.29	2622
1/Week	0.47	4268
1-3/Month	0.12	1082
A Few Times a Year	0.09	814
Never	0.03	288
Missing	0.00	1
Immigration Status		
Born in This Country	1.00	9048
Born in Another Country	0.00	25
Missing	0.00	1

Religion		
Christianity	0.62	5647
Islam	0.35	3189
Hinduism	.	.
Buddhism	.	.
Judaism	.	.
Sikhism	.	.
Baha'i	.	.
Jainism	.	.
Shinto	.	.
Taoism	0.00	1
Confucianism	.	.
Primal, Animist, or Folk Religion	0.00	12
Spiritism	.	.
African-Derived	.	.
Chinese	.	.
Some Other Religion	.	.
No Religion/Atheist/Agnostic	0.02	216
Missing	0.00	10
Race/Ethnicity		
African	1.00	9060
Indian	0.00	3
Arab	0.00	11
Other	.	.
Missing	0.00	2

Table 19b: Variations Across Demographic Characteristics (Tanzania)

Variable	Mean/Proportion	SE	LCI	UCI	Global p-value
Age					
18-24	0.90	0.01	0.88	0.92	0.00
25-29	0.89	0.01	0.87	0.91	.
30-39	0.89	0.01	0.87	0.91	.
40-49	0.90	0.01	0.88	0.93	.
50-59	0.94	0.01	0.92	0.95	.
60-69	0.94	0.01	0.91	0.96	.
70-79	0.97	0.02	0.93	1.00	.
80 or Older	0.91	0.06	0.78	1.04	.
Gender					
Male	0.89	0.01	0.87	0.90	0.00
Female	0.92	0.01	0.91	0.93	.
Other	.	.	.	.	.
Marital Status					
Single/Never Been Married	0.89	0.01	0.87	0.91	0.00
Married	0.91	0.01	0.90	0.93	.
Separated	0.87	0.02	0.83	0.90	.
Divorced	0.87	0.04	0.78	0.96	.
Widowed	0.96	0.01	0.94	0.99	.
Domestic Partner	0.86	0.02	0.82	0.91	.
Employment					
Employed for an Employer	0.91	0.01	0.88	0.94	0.09
Self-Employed	0.90	0.01	0.89	0.92	.
Retired	0.92	0.02	0.88	0.97	.
Student	0.93	0.02	0.90	0.97	.
Homemaker	0.92	0.01	0.91	0.94	.
Unemployed and Looking for a Job	0.89	0.01	0.86	0.91	.
None of These/Other	0.91	0.02	0.86	0.96	.
Education					
Up to 8 Years	0.91	0.01	0.89	0.92	0.90
9-15 Years	0.90	0.01	0.89	0.92	.
16+ Years	0.89	0.02	0.85	0.94	.
Service Attendance					
>1/Week	0.94	0.01	0.93	0.96	0.00
1/Week	0.92	0.01	0.90	0.93	.
1-3/Month	0.87	0.01	0.85	0.90	.
A Few Times a Year	0.82	0.02	0.78	0.85	.
Never	0.74	0.03	0.67	0.80	.
Immigration Status					
Born in This Country	0.91	0.01	0.90	0.92	0.17
Born in Another Country	0.73	0.11	0.47	0.99	.
Religion					
Christianity	0.91	0.01	0.90	0.93	0.00
Islam	0.89	0.01	0.87	0.91	.
Hinduism	.	.	.	.	.
Buddhism	.	.	.	.	.
Judaism	.	.	.	.	.

Sikhism	.	.	.	.	.
Baha'i	.	.	.	.	.
Jainism	.	.	.	.	.
Shinto	.	.	.	.	.
Taoism	0.00	.	.	.	.
Confucianism	.	.	.	.	.
Primal, Animist, or Folk Religion	0.86	0.01	0.83	0.90	.
Spiritism	.	.	.	.	.
African-Derived	.	.	.	.	.
Chinese	.	.	.	.	.
Some Other Religion	.	.	.	.	.
No Religion/Atheist/Agnostic	0.86	0.04	0.78	0.94	.
Race/Ethnicity					
African	0.91	0.01	0.89	0.92	0.00
Indian	1.00	.	.	.	.
Arab	1.00	.	.	.	.
Other	.	.	.	.	.

Table 20a: Nationally-Representative Descriptive Statistics of the Observed Sample (Turkey)

Variable	Proportion	Frequency
Age		
18-24	0.15	222
25-29	0.10	152
30-39	0.21	315
40-49	0.21	312
50-59	0.15	225
60-69	0.11	164
70-79	0.04	65
80 or Older	0.01	18
Missing	.	.
Gender		
Male	0.51	754
Female	0.49	719
Other	.	.
Missing	.	.
Marital Status		
Single/Never Been Married	0.26	379
Married	0.64	936
Separated	0.01	13
Divorced	0.04	64
Widowed	0.04	64
Domestic Partner	.	.
Missing	0.01	17
Employment		
Employed for an Employer	0.28	413
Self-Employed	0.17	255
Retired	0.14	205
Student	0.07	107
Homemaker	0.24	347
Unemployed and Looking for a Job	0.06	87
None of These/Other	0.04	59
Missing	.	.
Education		
Up to 8 Years	0.30	436
9-15 Years	0.48	711
16+ Years	0.22	326
Missing	.	.
Service Attendance		
>1/Week	0.33	493
1/Week	0.18	271
1-3/Month	0.12	174
A Few Times a Year	0.17	255
Never	0.19	274
Missing	0.00	6
Immigration Status		
Born in This Country	0.96	1415
Born in Another Country	0.04	58
Missing	.	.

Religion		
Christianity	0.00	2
Islam	0.94	1381
Hinduism	.	.
Buddhism	0.00	0
Judaism	0.00	1
Sikhism	0.00	1
Baha'i	.	.
Jainism	.	.
Shinto	.	.
Taoism	.	.
Confucianism	.	.
Primal, Animist, or Folk Religion	0.00	1
Spiritism	.	.
African-Derived	.	.
Chinese	.	.
Some Other Religion	0.00	1
No Religion/Atheist/Agnostic	0.04	66
Missing	0.01	19
Race/Ethnicity		
Turkish	0.70	1030
Kurdish/Zaza	0.17	252
Arab	0.03	51
Laz	0.02	25
Circassian	0.01	19
Bosnian	0.00	5
Armenian	0.00	1
Georgian	0.00	4
Uyghur	0.00	1
Jewish	.	.
Albanian	0.01	8
Greek	0.00	1
Azeri	0.01	9
Other	0.04	58
Missing	0.01	9

Table 20b: Variations Across Demographic Characteristics (Turkey)

Variable	Mean/Proportion	SE	LCI	UCI	Global p-value
Age					
18-24	0.60	0.03	0.54	0.67	0.00
25-29	0.72	0.04	0.65	0.80	.
30-39	0.73	0.03	0.68	0.78	.
40-49	0.72	0.03	0.67	0.77	.
50-59	0.75	0.03	0.69	0.82	.
60-69	0.74	0.05	0.64	0.84	.
70-79	0.66	0.09	0.47	0.86	.
80 or Older	0.95	0.01	0.00	1.90	.
Gender					
Male	0.69	0.02	0.65	0.72	0.07
Female	0.74	0.02	0.70	0.78	.
Other	.	.	.	.	.
Marital Status					
Single/Never Been Married	0.61	0.02	0.56	0.66	0.00
Married	0.75	0.02	0.72	0.78	.
Separated	0.74	0.10	0.45	1.04	.
Divorced	0.62	0.06	0.51	0.74	.
Widowed	0.88	0.04	0.80	0.97	.
Domestic Partner	.	.	.	.	.
Employment					
Employed for an Employer	0.68	0.02	0.63	0.72	0.00
Self-Employed	0.69	0.03	0.63	0.75	.
Retired	0.73	0.04	0.66	0.81	.
Student	0.58	0.04	0.49	0.66	.
Homemaker	0.81	0.03	0.76	0.87	.
Unemployed and Looking for a Job	0.66	0.05	0.56	0.76	.
None of These/Other	0.73	0.07	0.58	0.87	.
Education					
Up to 8 Years	0.85	0.03	0.80	0.90	0.00
9-15 Years	0.72	0.02	0.69	0.76	.
16+ Years	0.51	0.02	0.46	0.55	.
Service Attendance					
>1/Week	0.91	0.02	0.88	0.95	0.00
1/Week	0.82	0.03	0.76	0.87	.
1-3/Month	0.73	0.04	0.66	0.81	.
A Few Times a Year	0.53	0.03	0.46	0.60	.
Never	0.41	0.04	0.34	0.48	.
Immigration Status					
Born in This Country	0.71	0.01	0.69	0.74	0.78
Born in Another Country	0.73	0.07	0.59	0.87	.
Religion					
Christianity	0.25	0.01	-1.88	2.38	0.00
Islam	0.75	0.01	0.72	0.77	.
Hinduism	.	.	.	.	.
Buddhism	0.00	.	.	.	.

Judaism	1.00	.	.	.	.
Sikhism	0.00	.	.	.	.
Baha'i	.	.	.	.	.
Jainism	.	.	.	.	.
Shinto	.	.	.	.	.
Taoism	.	.	.	.	.
Confucianism	.	.	.	.	.
Primal, Animist, or Folk Religion	0.00	.	.	.	.
Spiritism	.	.	.	.	.
African-Derived	.	.	.	.	.
Chinese	.	.	.	.	.
Some Other Religion	0.00	.	.	.	.
No Religion/Atheist/Agnostic	0.09	0.04	0.02	0.16	.
Race/Ethnicity					
Turkish	0.69	0.02	0.66	0.72	0.00
Kurdish/Zaza	0.78	0.03	0.72	0.84	.
Arab	0.91	0.05	0.82	1.01	.
Laz	0.65	0.12	0.40	0.90	.
Circassian	0.70	0.08	0.50	0.89	.
Bosnian	0.56	0.30	-2.07	3.19	.
Armenian	1.00	.	.	.	.
Georgian	0.85	0.15	-113.52	115.22	.
Uyghur	1.00	.	.	.	.
Jewish	.	.	.	.	.
Albanian	0.44	0.11	-17.42	18.29	.
Greek	0.00	.	.	.	.
Azeri	0.72	0.12	0.33	1.11	.
Other	0.68	0.07	0.53	0.82	.

Table 21a: Nationally-Representative Descriptive Statistics of the Observed Sample (United Kingdom)

Variable	Proportion	Frequency
Age		
18-24	0.09	490
25-29	0.07	391
30-39	0.18	946
40-49	0.15	827
50-59	0.18	949
60-69	0.17	889
70-79	0.13	711
80 or Older	0.03	163
Missing	0.00	1
Gender		
Male	0.48	2557
Female	0.52	2789
Other	0.00	14
Missing	0.00	9
Marital Status		
Single/Never Been Married	0.27	1456
Married	0.47	2510
Separated	0.02	114
Divorced	0.08	435
Widowed	0.05	294
Domestic Partner	0.10	512
Missing	0.01	48
Employment		
Employed for an Employer	0.52	2798
Self-Employed	0.09	469
Retired	0.24	1262
Student	0.04	229
Homemaker	0.03	184
Unemployed and Looking for a Job	0.04	215
None of These/Other	0.04	201
Missing	0.00	11
Education		
Up to 8 Years	0.24	1314
9-15 Years	0.39	2072
16+ Years	0.37	1974
Missing	0.00	8
Service Attendance		
>1/Week	0.05	291
1/Week	0.09	499
1-3/Month	0.05	293
A Few Times a Year	0.22	1165
Never	0.58	3110
Missing	0.00	10
Immigration Status		
Born in This Country	0.87	4659
Born in Another Country	0.13	682

Missing	0.00	27
Religion		
Christianity	0.51	2750
Islam	0.04	218
Hinduism	0.01	61
Buddhism	0.01	30
Judaism	0.01	44
Sikhism	0.01	29
Baha'i	0.00	6
Jainism	0.00	4
Shinto	.	.
Taoism	0.00	4
Confucianism	0.00	2
Primal, Animist, or Folk Religion	0.01	36
Spiritism	.	.
African-Derived	.	.
Chinese	.	.
Some Other Religion	0.01	61
No Religion/Atheist/Agnostic	0.39	2099
Missing	0.00	25
Race/Ethnicity		
Asian	0.08	426
Black	0.03	152
White	0.87	4647
Other	0.02	96
Missing	0.01	47

Table 21b: Variations Across Demographic Characteristics (United Kingdom)

Variable	Mean/Proportion	SE	LCI	UCI	Global p-value
Age					
18-24	0.29	0.03	0.22	0.36	0.00
25-29	0.35	0.03	0.29	0.42	.
30-39	0.33	0.02	0.29	0.37	.
40-49	0.29	0.02	0.24	0.33	.
50-59	0.24	0.02	0.20	0.27	.
60-69	0.23	0.02	0.19	0.26	.
70-79	0.28	0.02	0.24	0.33	.
80 or Older	0.30	0.05	0.21	0.39	.
Gender					
Male	0.30	0.01	0.27	0.32	0.00
Female	0.27	0.01	0.24	0.29	.
Other	0.05	0.04	-0.03	0.14	.
Marital Status					
Single/Never Been Married	0.24	0.02	0.21	0.28	0.00
Married	0.35	0.01	0.32	0.38	.
Separated	0.25	0.05	0.15	0.35	.
Divorced	0.20	0.03	0.15	0.25	.
Widowed	0.30	0.04	0.23	0.37	.
Domestic Partner	0.10	0.02	0.06	0.14	.
Employment					
Employed for an Employer	0.30	0.01	0.28	0.32	0.00
Self-Employed	0.27	0.03	0.21	0.33	.
Retired	0.26	0.02	0.23	0.30	.
Student	0.30	0.05	0.21	0.39	.
Homemaker	0.31	0.05	0.21	0.41	.
Unemployed and Looking for a Job	0.23	0.05	0.14	0.32	.
None of These/Other	0.14	0.03	0.09	0.20	.
Education					
Up to 8 Years	0.26	0.02	0.22	0.31	0.00
9-15 Years	0.23	0.01	0.21	0.25	.
16+ Years	0.34	0.01	0.32	0.37	.
Service Attendance					
>1/Week	0.89	0.03	0.84	0.94	0.00
1/Week	0.75	0.03	0.70	0.80	.
1-3/Month	0.62	0.04	0.54	0.70	.
A Few Times a Year	0.34	0.02	0.30	0.38	.
Never	0.09	0.01	0.08	0.11	.
Immigration Status					
Born in This Country	0.26	0.01	0.24	0.28	0.00
Born in Another Country	0.41	0.03	0.36	0.47	.
Religion					
Christianity	0.39	0.01	0.36	0.42	0.00
Islam	0.83	0.04	0.75	0.91	.
Hinduism	0.45	0.09	0.28	0.63	.
Buddhism	0.58	0.10	0.36	0.79	.

Judaism	0.28	0.10	0.08	0.49	.
Sikhism	0.51	0.14	0.20	0.82	.
Baha'i	0.14	0.17	-27.78	28.06	.
Jainism	0.00	.	.	.	.
Shinto	.	.	.	.	.
Taoism	0.26	0.21	-35.02	35.54	.
Confucianism	0.00	.	.	.	.
Primal, Animist, or Folk Religion	0.21	0.09	0.02	0.41	.
Spiritism	.	.	.	.	.
African-Derived	.	.	.	.	.
Chinese	.	.	.	.	.
Some Other Religion	0.41	0.07	0.26	0.56	.
No Religion/Atheist/Agnostic	0.06	0.01	0.05	0.08	.
Race/Ethnicity					
Asian	0.59	0.03	0.52	0.66	0.00
Black	0.67	0.05	0.57	0.77	.
White	0.24	0.01	0.22	0.26	.
Other	0.29	0.06	0.16	0.42	.

Table 22a: Nationally-Representative Descriptive Statistics of the Observed Sample (United States)

Variable	Proportion	Frequency
Age		
18-24	0.07	2682
25-29	0.09	3540
30-39	0.19	7284
40-49	0.15	5649
50-59	0.18	6745
60-69	0.18	6832
70-79	0.11	4054
80 or Older	0.04	1525
Missing	.	.
Gender		
Male	0.48	18222
Female	0.51	19562
Other	0.01	392
Missing	0.00	136
Marital Status		
Single/Never Been Married	0.25	9431
Married	0.53	20360
Separated	0.02	727
Divorced	0.09	3636
Widowed	0.05	1978
Domestic Partner	0.05	1971
Missing	0.01	207
Employment		
Employed for an Employer	0.51	19502
Self-Employed	0.09	3445
Retired	0.24	9016
Student	0.03	1144
Homemaker	0.05	2049
Unemployed and Looking for a Job	0.05	1777
None of These/Other	0.03	1292
Missing	0.00	87
Education		
Up to 8 Years	0.01	210
9-15 Years	0.66	25322
16+ Years	0.33	12705
Missing	0.00	75
Service Attendance		
>1/Week	0.07	2633
1/Week	0.15	5887
1-3/Month	0.07	2819
A Few Times a Year	0.23	8870
Never	0.47	17975
Missing	0.00	128
Immigration Status		
Born in This Country	0.91	34865
Born in Another Country	0.08	3020
Missing	0.01	427

Religion		
Christianity	0.60	22954
Islam	0.01	205
Hinduism	0.00	167
Buddhism	0.01	336
Judaism	0.02	638
Sikhism	0.00	24
Baha'i	0.00	13
Jainism	0.00	18
Shinto	0.00	12
Taoism	0.00	93
Confucianism	0.00	8
Primal, Animist, or Folk Religion	0.01	240
Spiritism	.	.
African-Derived	.	.
Chinese	.	.
Some Other Religion	0.03	1267
No Religion/Atheist/Agnostic	0.31	11870
Missing	0.01	467
Race/Ethnicity		
White	0.62	23605
Other	0.03	997
Black	0.12	4501
Asian	0.06	2466
Hispanic	0.18	6724
Other	.	.
Missing	0.00	20

Table 22b: Variations Across Demographic Characteristics (United States)

Variable	Mean/Proportion	SE	LCI	UCI	Global p-value
Age					
18-24	0.29	0.04	0.22	0.37	0.00
25-29	0.32	0.03	0.26	0.37	.
30-39	0.33	0.01	0.31	0.36	.
40-49	0.42	0.01	0.39	0.45	.
50-59	0.51	0.01	0.48	0.53	.
60-69	0.54	0.01	0.53	0.56	.
70-79	0.53	0.01	0.51	0.55	.
80 or Older	0.59	0.02	0.54	0.63	.
Gender					
Male	0.40	0.01	0.39	0.42	0.00
Female	0.48	0.01	0.47	0.50	.
Other	0.21	0.08	0.04	0.37	.
Marital Status					
Single/Never Been Married	0.32	0.02	0.29	0.35	0.00
Married	0.49	0.01	0.48	0.50	.
Separated	0.54	0.06	0.42	0.66	.
Divorced	0.48	0.01	0.45	0.51	.
Widowed	0.57	0.02	0.54	0.61	.
Domestic Partner	0.26	0.03	0.21	0.31	.
Employment					
Employed for an Employer	0.41	0.01	0.40	0.43	0.00
Self-Employed	0.45	0.02	0.41	0.50	.
Retired	0.54	0.01	0.53	0.56	.
Student	0.21	0.04	0.14	0.29	.
Homemaker	0.52	0.02	0.47	0.56	.
Unemployed and Looking for a Job	0.31	0.05	0.22	0.41	.
None of These/Other	0.38	0.04	0.31	0.46	.
Education					
Up to 8 Years	0.38	0.14	0.10	0.66	0.00
9-15 Years	0.45	0.01	0.44	0.47	.
16+ Years	0.42	0.00	0.41	0.43	.
Service Attendance					
>1/Week	0.96	0.01	0.94	0.97	0.00
1/Week	0.86	0.01	0.84	0.88	.
1-3/Month	0.67	0.02	0.63	0.72	.
A Few Times a Year	0.46	0.01	0.43	0.48	.
Never	0.18	0.01	0.17	0.20	.
Immigration Status					
Born in This Country	0.44	0.01	0.43	0.45	0.23
Born in Another Country	0.41	0.03	0.36	0.46	.
Religion					
Christianity	0.64	0.01	0.63	0.65	0.00
Islam	0.52	0.09	0.34	0.69	.
Hinduism	0.43	0.07	0.29	0.57	.
Buddhism	0.48	0.06	0.37	0.60	.
Judaism	0.31	0.03	0.25	0.37	.

Sikhism	0.39	0.16	0.05	0.73	.
Baha'i	0.59	0.16	0.25	0.94	.
Jainism	0.82	0.15	0.46	1.17	.
Shinto	0.56	0.23	0.04	1.08	.
Taoism	0.16	0.08	0.00	0.32	.
Confucianism	0.48	0.28	-0.31	1.27	.
Primal, Animist, or Folk Religion	0.57	0.12	0.34	0.81	.
Spiritism	.	.	.	.	.
African-Derived	.	.	.	.	.
Chinese	.	.	.	.	.
Some Other Religion	0.40	0.04	0.33	0.47	.
No Religion/Atheist/Agnostic	0.06	0.01	0.05	0.07	.
Race/Ethnicity					
White	0.44	0.01	0.43	0.45	0.00
Other	0.55	0.03	0.49	0.60	.
Black	0.54	0.02	0.50	0.58	.
Asian	0.32	0.02	0.28	0.37	.
Hispanic	0.40	0.02	0.36	0.45	.
Other	.	.	.	.	.

Table S23. Population weighted meta-analysis of results demographic group means.

Variable	Category	Proportion	95% CI of Proportion	SE Analogue (CI Width/4)
Age group				
	18-24	0.44	(0.41,0.48)	0.02
	25-29	0.47	(0.43,0.50)	0.02
	30-39	0.49	(0.45,0.52)	0.02
	40-49	0.52	(0.48,0.56)	0.02
	50-59	0.58	(0.54,0.61)	0.02
	60-69	0.58	(0.54,0.62)	0.02
	70-79	0.60	(0.56,0.64)	0.02
	80 or older	0.89	(0.84,0.92)	0.02
Gender				
	Male	0.50	(0.47,0.54)	0.02
	Female	0.54	(0.50,0.58)	0.02
	Other	0.17	(0.10,0.28)	0.04
Marital status				
	Married	0.59	(0.55,0.62)	0.02
	Separated	0.40	(0.36,0.44)	0.02
	Divorced	0.48	(0.44,0.51)	0.02
	Widowed	0.48	(0.43,0.53)	0.02
	Domestic partner	0.30	(0.27,0.34)	0.02
	Single, never married	0.44	(0.41,0.48)	0.02
Employment status				
	Employed for an employer	0.50	(0.47,0.54)	0.02
	Self-employed	0.55	(0.51,0.58)	0.02
	Retired	0.59	(0.55,0.63)	0.02
	Student	0.39	(0.36,0.43)	0.02
	Homemaker	0.57	(0.53,0.61)	0.02
	Unemployed and looking for a job	0.47	(0.43,0.51)	0.02
	None of these/other	0.45	(0.41,0.48)	0.02
Education				
	Up to 8 years	0.56	(0.52,0.59)	0.02
	9-15 years	0.49	(0.45,0.52)	0.02
	16+ years	0.50	(0.46,0.53)	0.02
Religious service attendance				
	>1/week	0.91	(0.90,0.93)	0.01

Variable	Category	Proportion	95% CI of Proportion	SE Analogue (CI Width/4)
	1/week	0.80	(0.78,0.83)	0.01
	1-3/month	0.66	(0.63,0.69)	0.02
	A few times a year	0.50	(0.46,0.53)	0.02
	Never	0.23	(0.20,0.26)	0.02
Immigration status				
	Born in this country	0.51	(0.48,0.55)	0.02
	Born in another country	0.56	(0.52,0.59)	0.02

Forest Plots

Figure S1. Forest plot for `Age group`-`18-24`

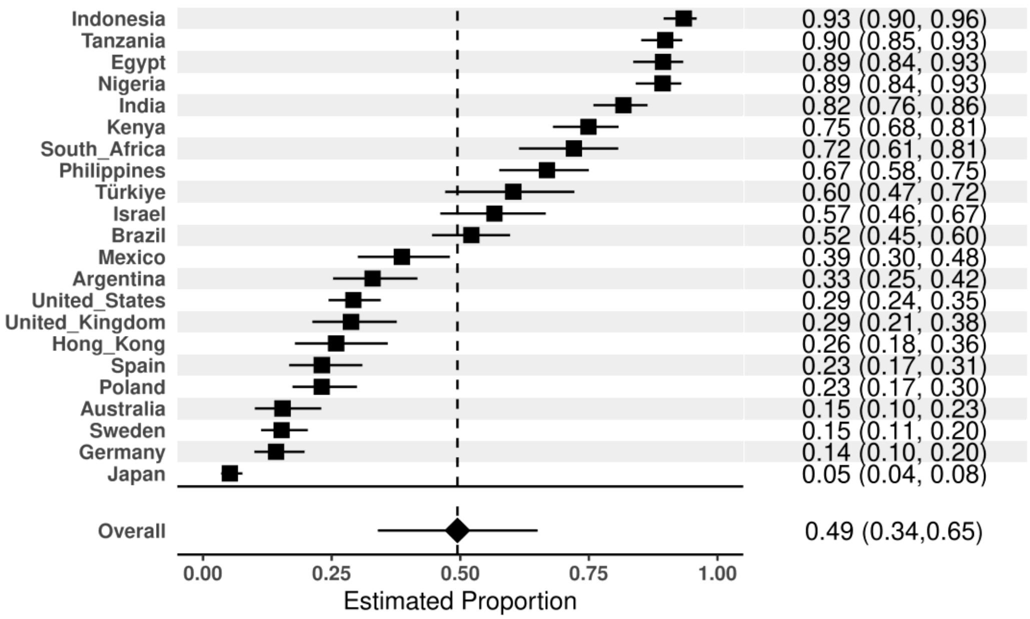

Probability-scale: τ=0.382;
Logit-scale: τ=1.528; Q-profile 95% CI [1.132, 2.080]; I^2=98.80;
Plot is based on back transformed bounds after using approximate logit SE that aren't guarenteed to match the robust SE of a proportion.
Excluded countries: Hong Kong, South Africa, United Kingdom, United States

Figure S2. Forest plot for `Age group`-`25-29`

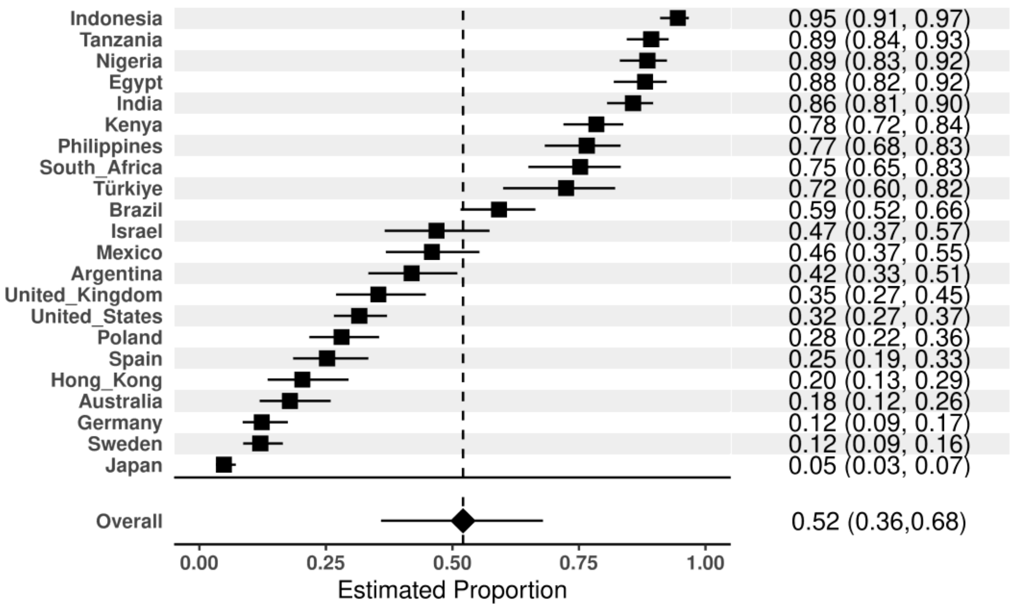

Probability-scale: τ=0.394;
Logit-scale: τ=1.581; Q-profile 95% CI [1.171, 2.151]; I^2=98.86;
Plot is based on back transformed bounds after using approximate logit SE that aren't guarenteed to match the robust SE of a proportion.
Excluded countries: Hong Kong, South Africa, United Kingdom, United States

Figure S3. Forest plot for `Age group`-`30-39`

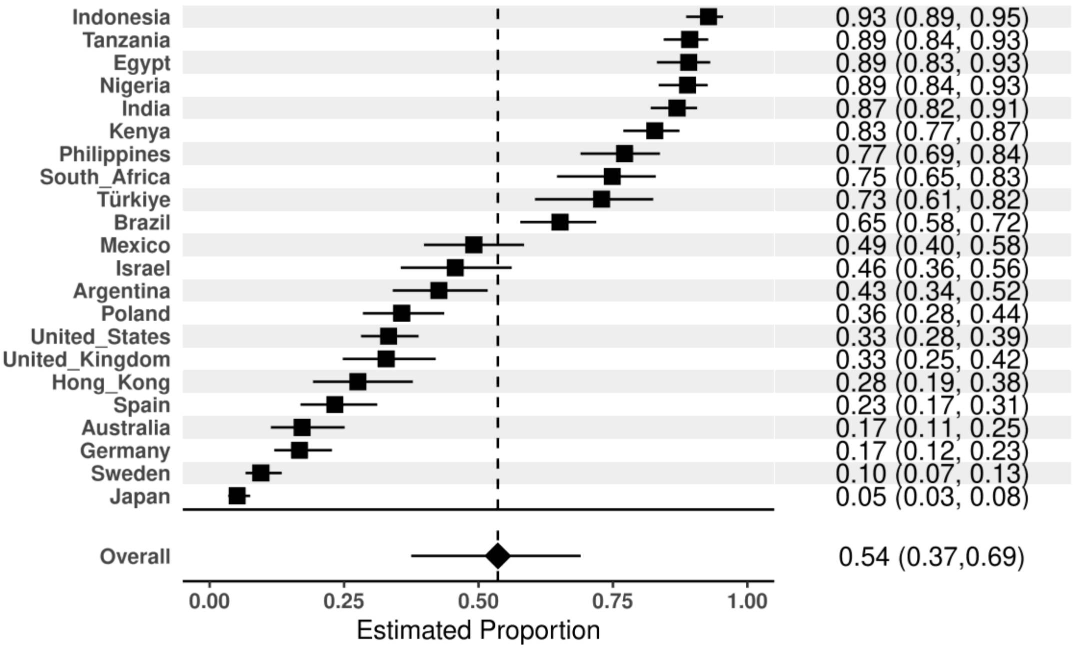

Probability-scale: τ=0.388;
Logit-scale: τ=1.560; Q-profile 95% CI [1.156, 2.123]; I^2=98.83;
Plot is based on back transformed bounds after using approximate logit SE that aren't guarenteed to match the robust SE of a proportion.
Excluded countries: Hong Kong, South Africa, United Kingdom, United States

Figure S4. Forest plot for `Age group`-`40-49`

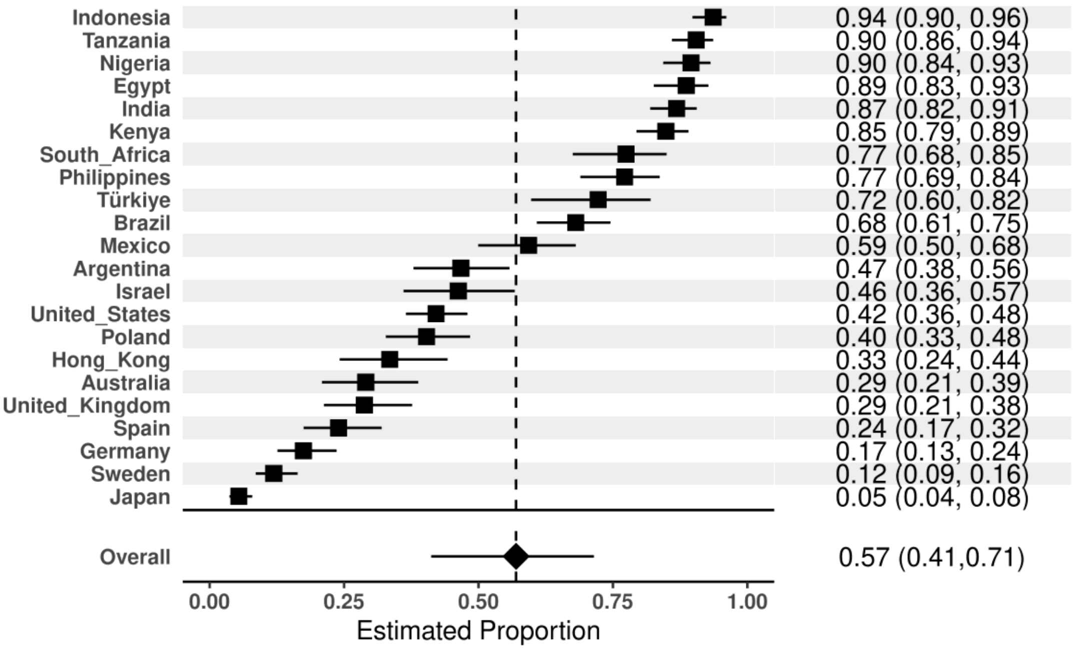

Probability-scale: τ=0.371;
Logit-scale: τ=1.513; Q-profile 95% CI [1.122, 2.060]; I^2=98.77;
Plot is based on back transformed bounds after using approximate logit SE that aren't guarenteed to match the robust SE of a proportion.
Excluded countries: Hong Kong, South Africa, United Kingdom, United States

Figure S5. Forest plot for `Age group`-`50-59`

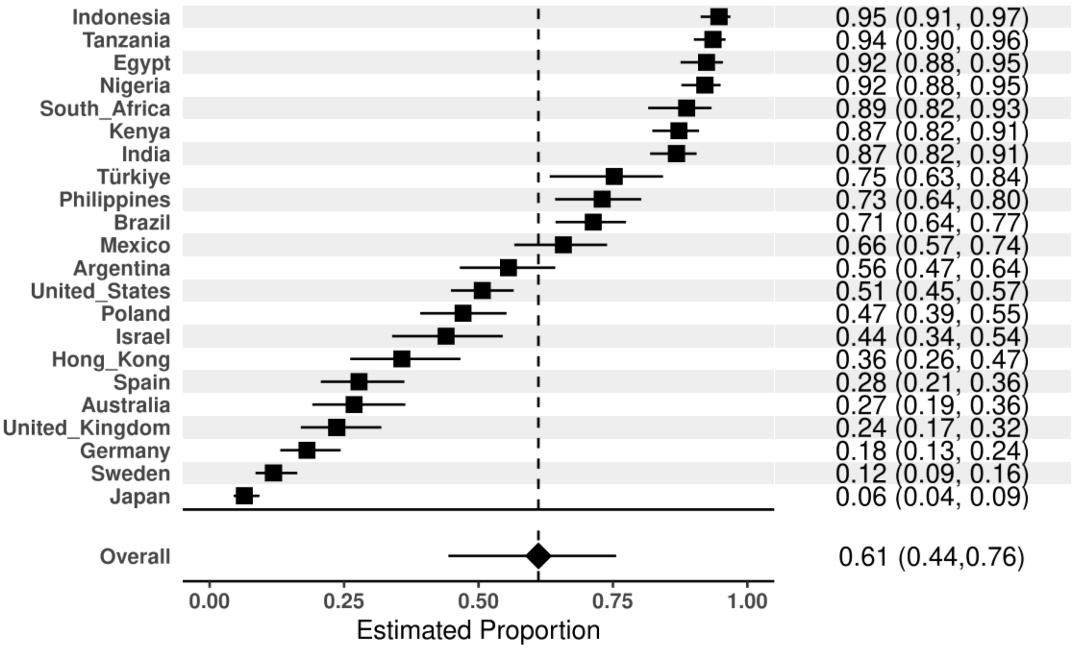

Probability-scale: τ=0.383;
Logit-scale: τ=1.613; Q-profile 95% CI [1.196, 2.195]; I^2=98.89;
Plot is based on back transformed bounds after using approximate logit SE that aren't guarenteed to match the robust SE of a proportion.
Excluded countries: Hong Kong, South Africa, United Kingdom, United States

Figure S6. Forest plot for `Age group`-`60-69`

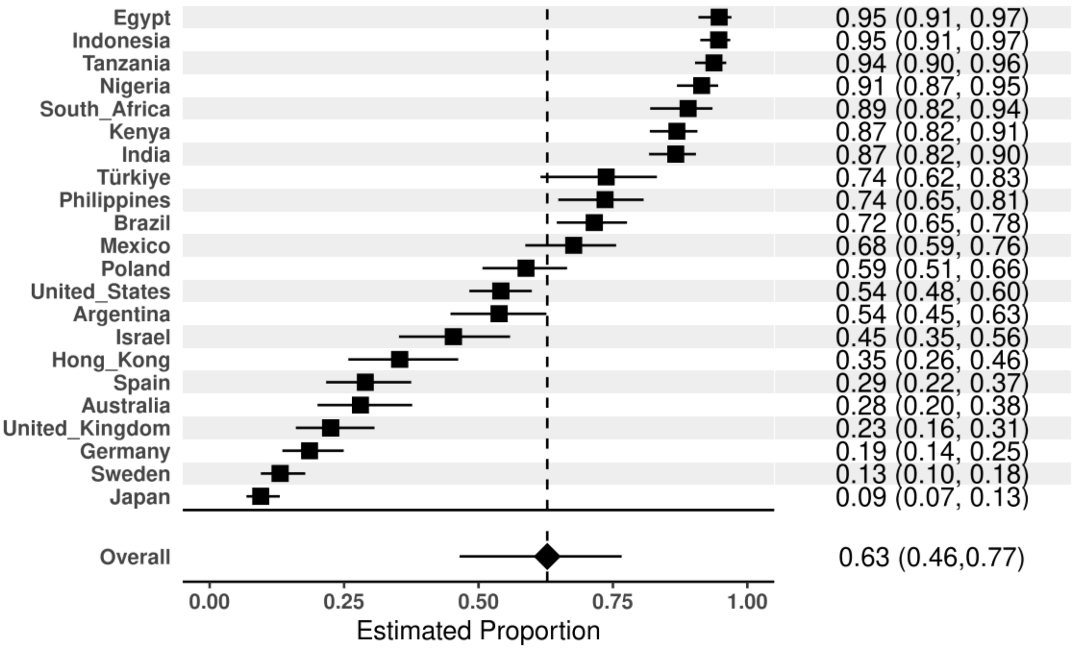

Probability-scale: τ=0.369;
Logit-scale: τ=1.579; Q-profile 95% CI [1.170, 2.149]; I^2=98.85;
Plot is based on back transformed bounds after using approximate logit SE that aren't guarenteed to match the robust SE of a proportion.
Excluded countries: Hong Kong, South Africa, United Kingdom, United States

Figure S7. Forest plot for `Age group`-`70-79`

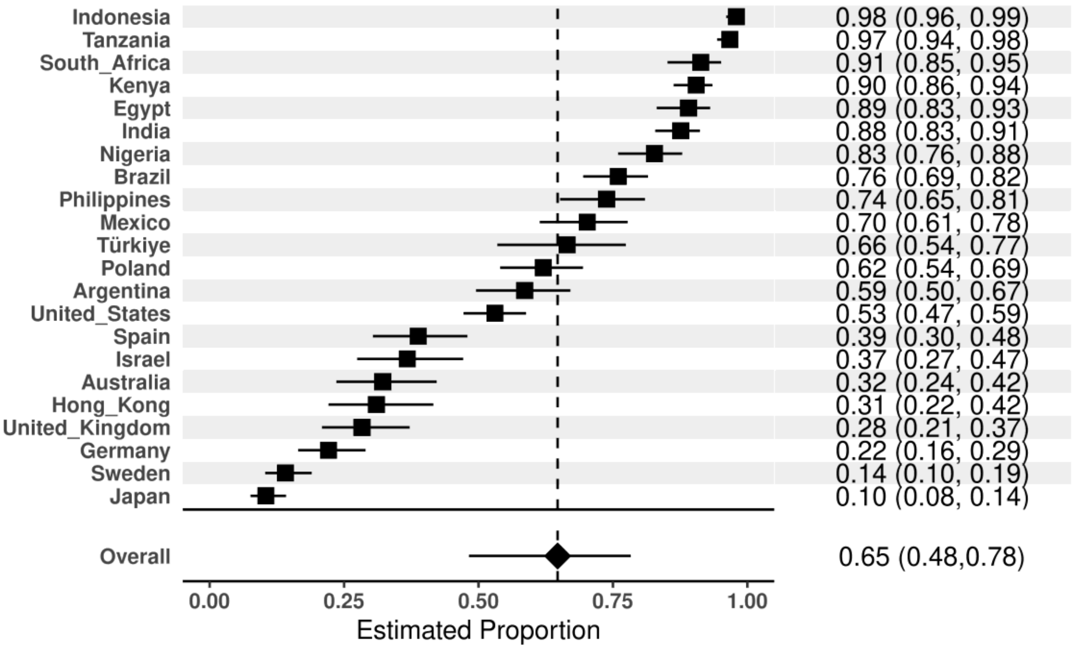

Probability-scale: τ=0.368;
Logit-scale: τ=1.611; Q-profile 95% CI [1.191, 2.192]; I^2=98.90;
Plot is based on back transformed bounds after using approximate logit SE that aren't guarenteed to match the robust SE of a proportion.
Excluded countries: Hong Kong, South Africa, United Kingdom, United States

Figure S8. Forest plot for `Age group`-`80 or older`

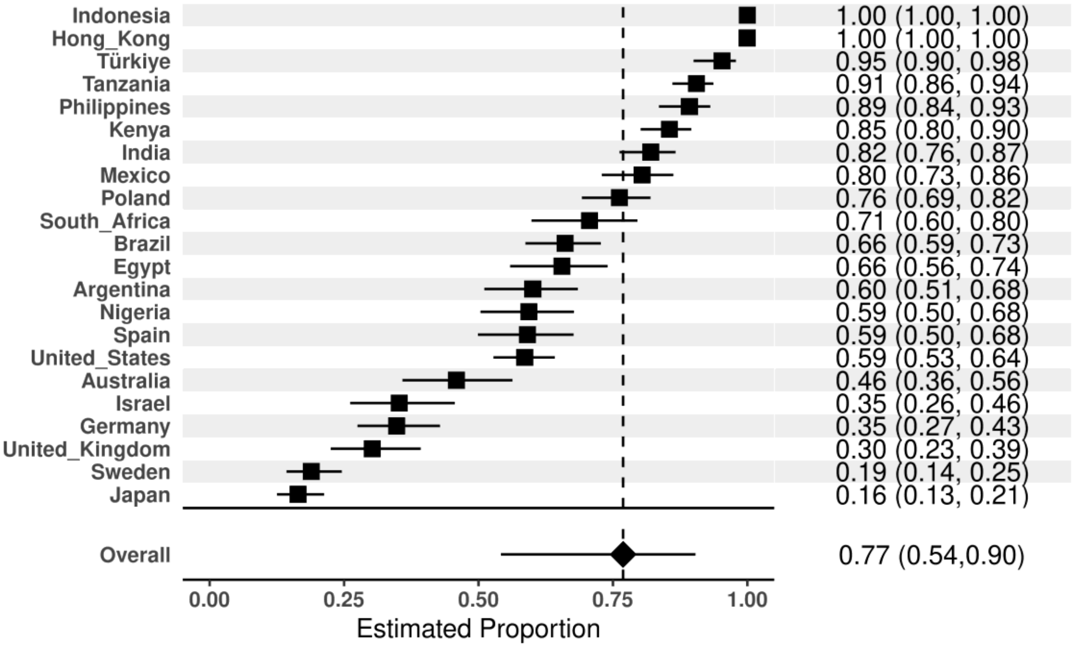

Probability-scale: τ=0.436;
Logit-scale: τ=2.455; Q-profile 95% CI [1.624, 3.271]; I^2=99.53;
Plot is based on back transformed bounds after using approximate logit SE that aren't guarenteed to match the robust SE of a proportion.
Excluded countries: Hong Kong, South Africa, United Kingdom, United States

Figure S9. Forest plot for `Gender`-`Male`

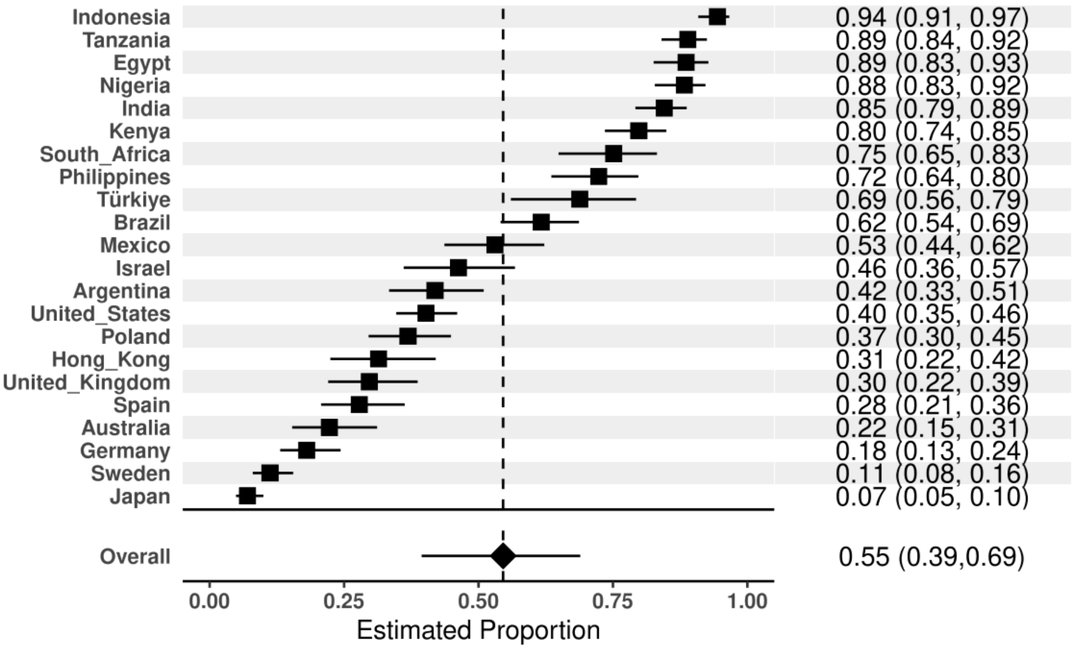

Probability-scale: τ=0.361;
Logit-scale: τ=1.457; Q-profile 95% CI [1.079, 1.983]; I^2=98.70;
Plot is based on back transformed bounds after using approximate logit SE that aren't guarenteed to match the robust SE of a proportion.
Excluded countries: Hong Kong, South Africa, United Kingdom, United States

Figure S10. Forest plot for `Gender`-`Female`

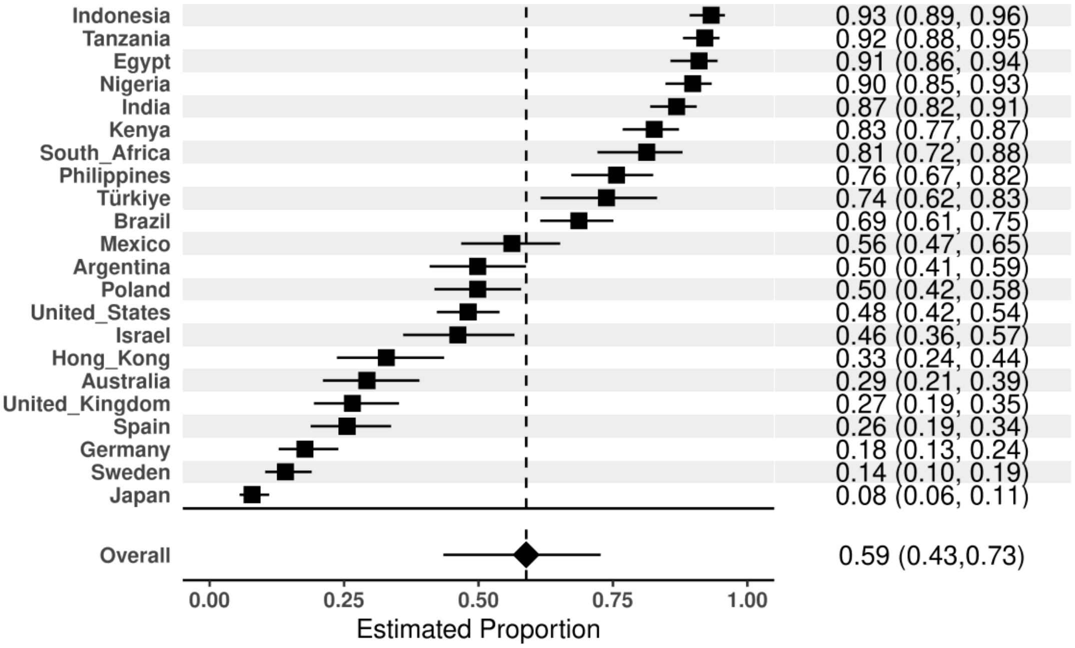

Probability-scale: τ=0.357;
Logit-scale: τ=1.476; Q-profile 95% CI [1.094, 2.010]; I^2=98.72;
Plot is based on back transformed bounds after using approximate logit SE that aren't guarenteed to match the robust SE of a proportion.
Excluded countries: Hong Kong, South Africa, United Kingdom, United States

Figure S11. Forest plot for `Gender`-`Other`

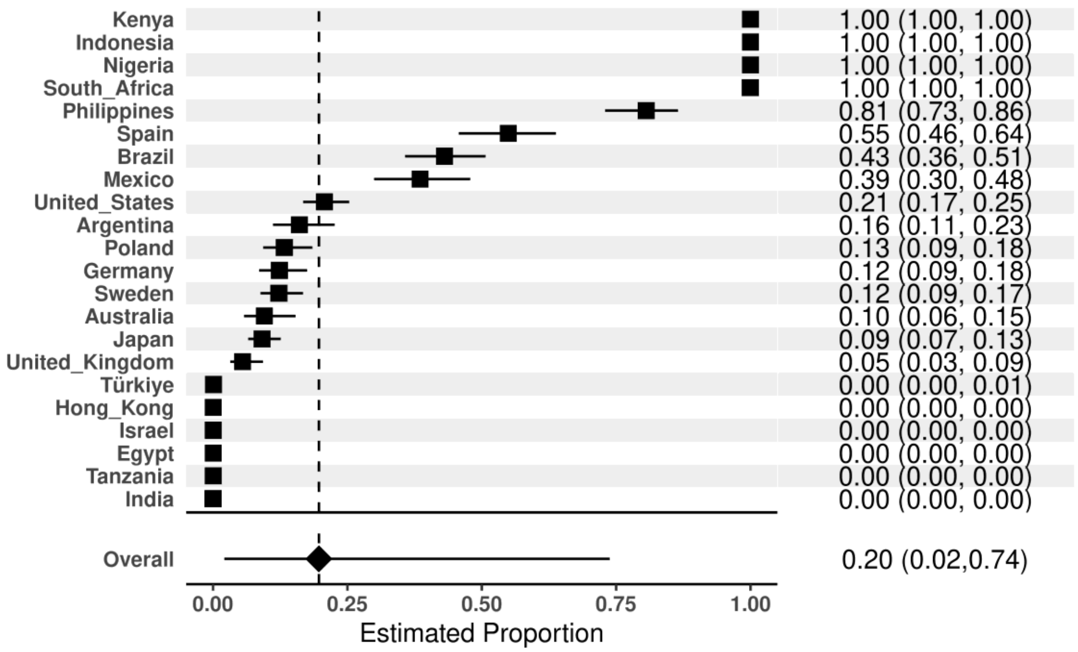

Probability-scale: τ=0.918;
Logit-scale: τ=5.802; Q-profile 95% CI [4.239, 7.871]; I^2=99.85;
Plot is based on back transformed bounds after using approximate logit SE that aren't guarenteed to match the robust SE of a proportion.
Excluded countries: Hong Kong, South Africa, United Kingdom, United States

Figure S12. Forest plot for `Marital status`-`Single, never married`

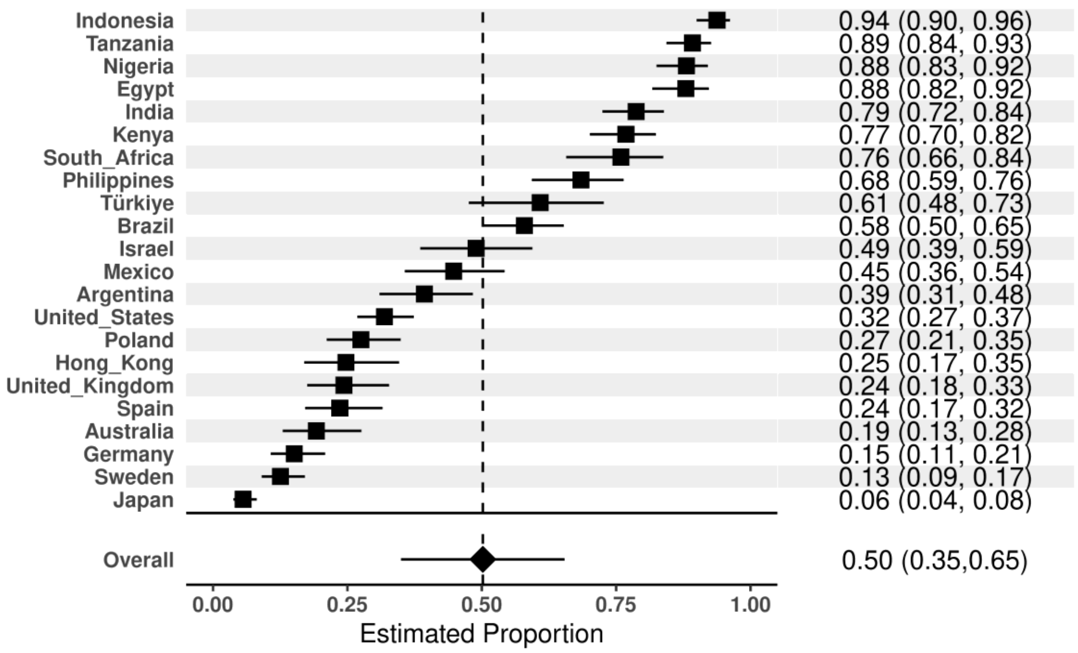

Probability-scale: τ=0.373;
Logit-scale: τ=1.494; Q-profile 95% CI [1.106, 2.033]; I^2=98.75;
Plot is based on back transformed bounds after using approximate logit SE that aren't guarenteed to match the robust SE of a proportion.
Excluded countries: Hong Kong, South Africa, United Kingdom, United States

Figure S13. Forest plot for `Marital status`-`Married`

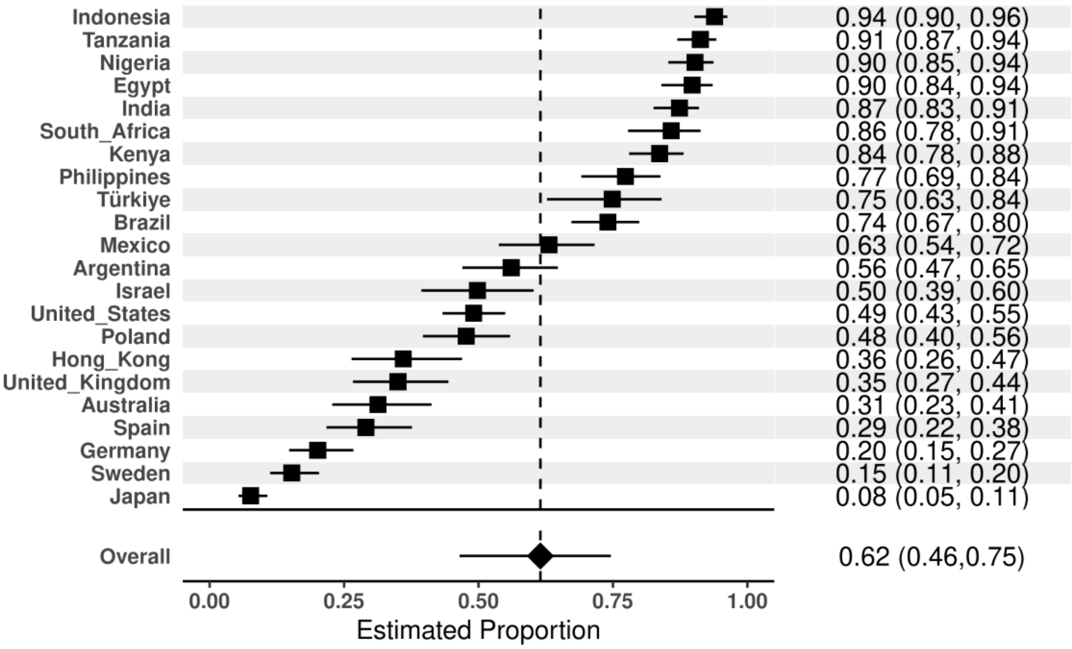

Probability-scale: τ=0.343;
Logit-scale: τ=1.448; Q-profile 95% CI [1.073, 1.972]; I^2=98.67;
Plot is based on back transformed bounds after using approximate logit SE that aren't guarenteed to match the robust SE of a proportion.
Excluded countries: Hong Kong, South Africa, United Kingdom, United States

Figure S14. Forest plot for `Marital status`-`Separated`

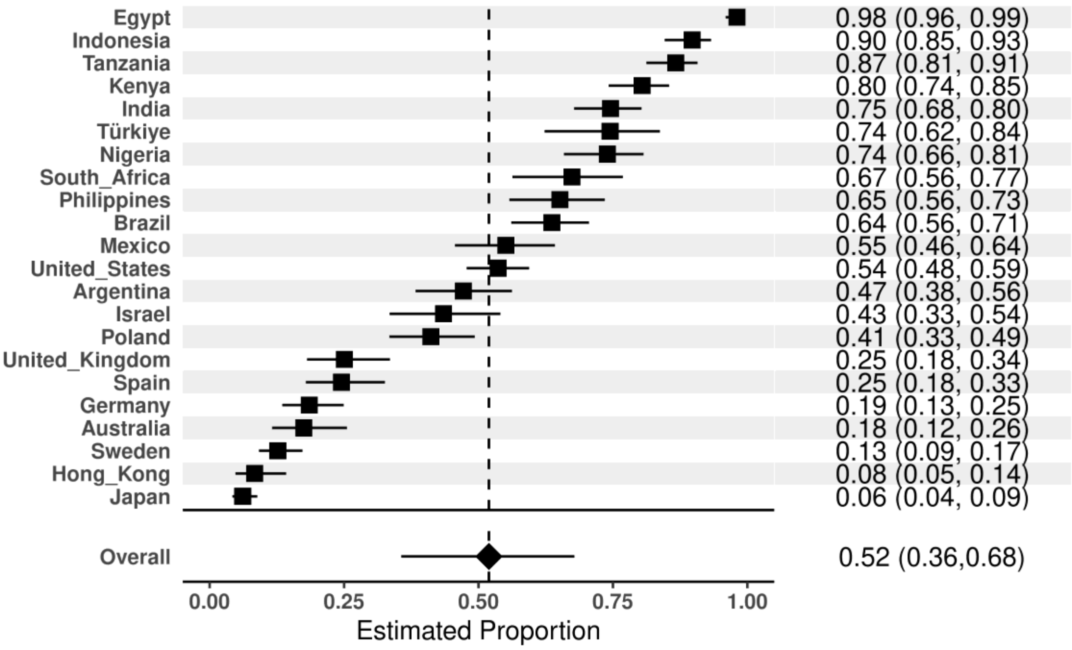

Probability-scale: τ=0.397;
Logit-scale: τ=1.590; Q-profile 95% CI [1.171, 2.161]; I^2=98.91;
Plot is based on back transformed bounds after using approximate logit SE that aren't guarenteed to match the robust SE of a proportion.
Excluded countries: Hong Kong, South Africa, United Kingdom, United States

Figure S15. Forest plot for `Marital status`-`Divorced`

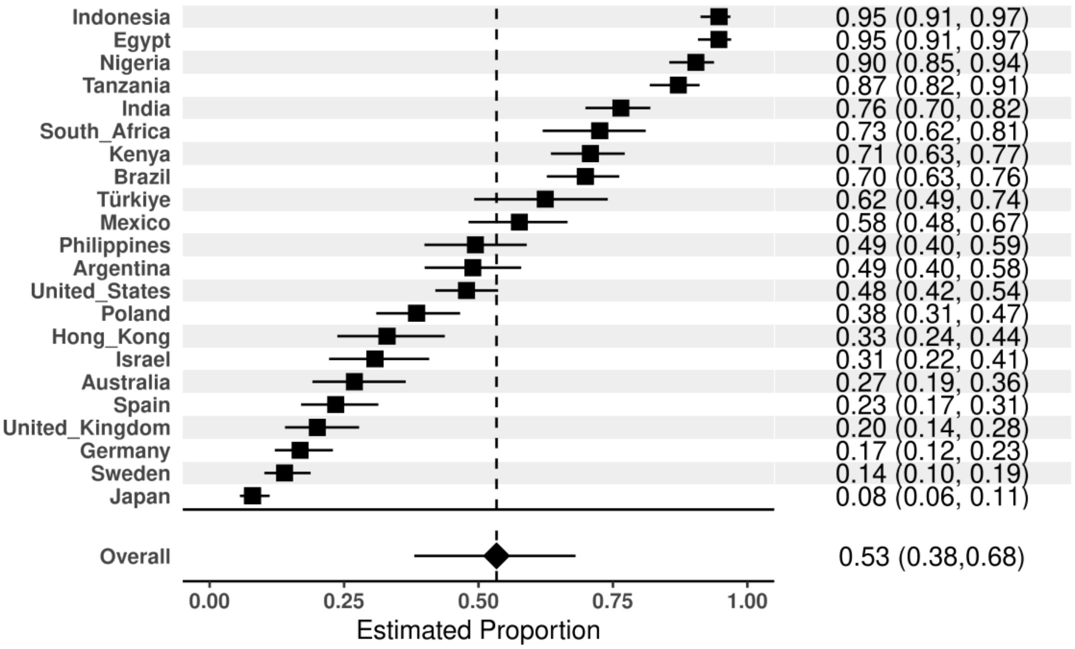

Probability-scale: τ=0.368;
Logit-scale: τ=1.477; Q-profile 95% CI [1.092, 2.010]; I^2=98.74;
Plot is based on back transformed bounds after using approximate logit SE that aren't guarenteed to match the robust SE of a proportion.
Excluded countries: Hong Kong, South Africa, United Kingdom, United States

Figure S16. Forest plot for `Marital status`-`Widowed`

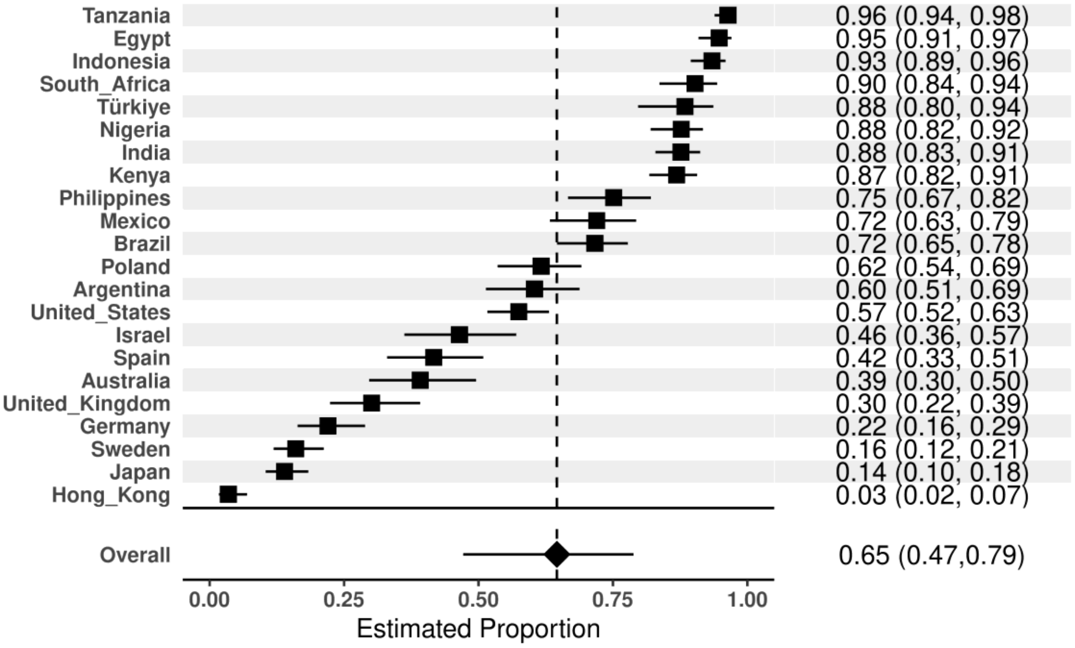

Probability-scale: τ=0.389;
Logit-scale: τ=1.699; Q-profile 95% CI [1.253, 2.309]; I^2=98.99;
Plot is based on back transformed bounds after using approximate logit SE that aren't guarenteed to match the robust SE of a proportion.
Excluded countries: Hong Kong, South Africa, United Kingdom, United States

Figure S17. Forest plot for `Marital status`-`Domestic partner`

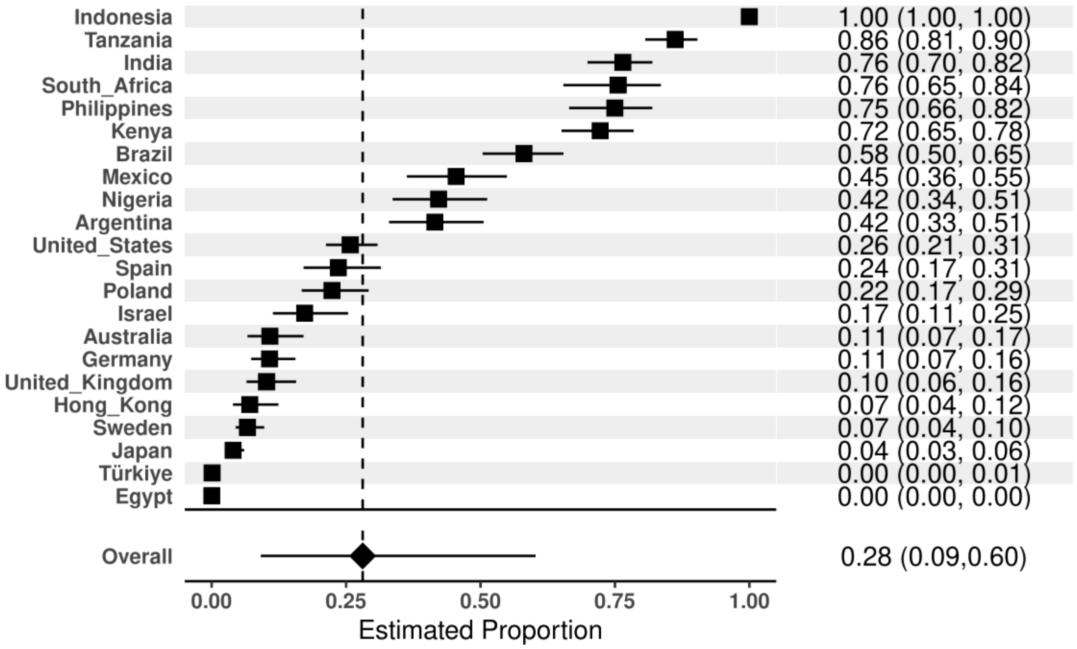

Probability-scale: τ=0.651;
Logit-scale: τ=3.222; Q-profile 95% CI [2.232, 4.323]; I^2=99.69;
Plot is based on back transformed bounds after using approximate logit SE that aren't guarenteed to match the robust SE of a proportion.
Excluded countries: Hong Kong, South Africa, United Kingdom, United States

Figure S18. Forest plot for `Employment status`-`Employed for an employer`

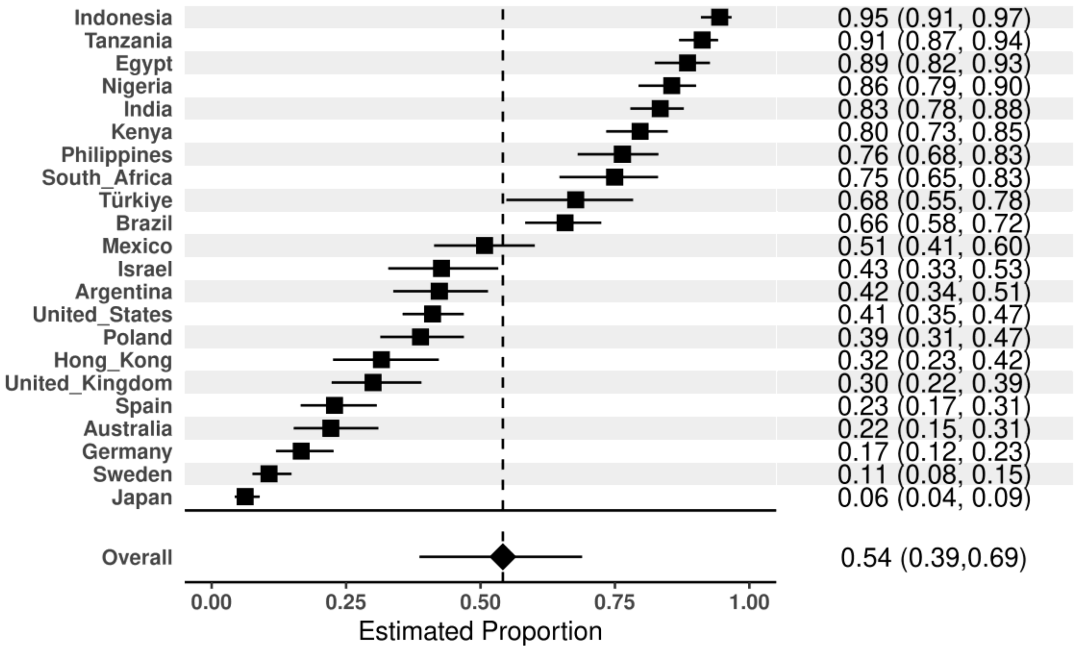

Probability-scale: τ=0.371;
Logit-scale: τ=1.495; Q-profile 95% CI [1.107, 2.035]; I^2=98.75;
Plot is based on back transformed bounds after using approximate logit SE that aren't guarenteed to match the robust SE of a proportion.
Excluded countries: Hong Kong, South Africa, United Kingdom, United States

Figure S19. Forest plot for `Employment status`-`Self-employed`

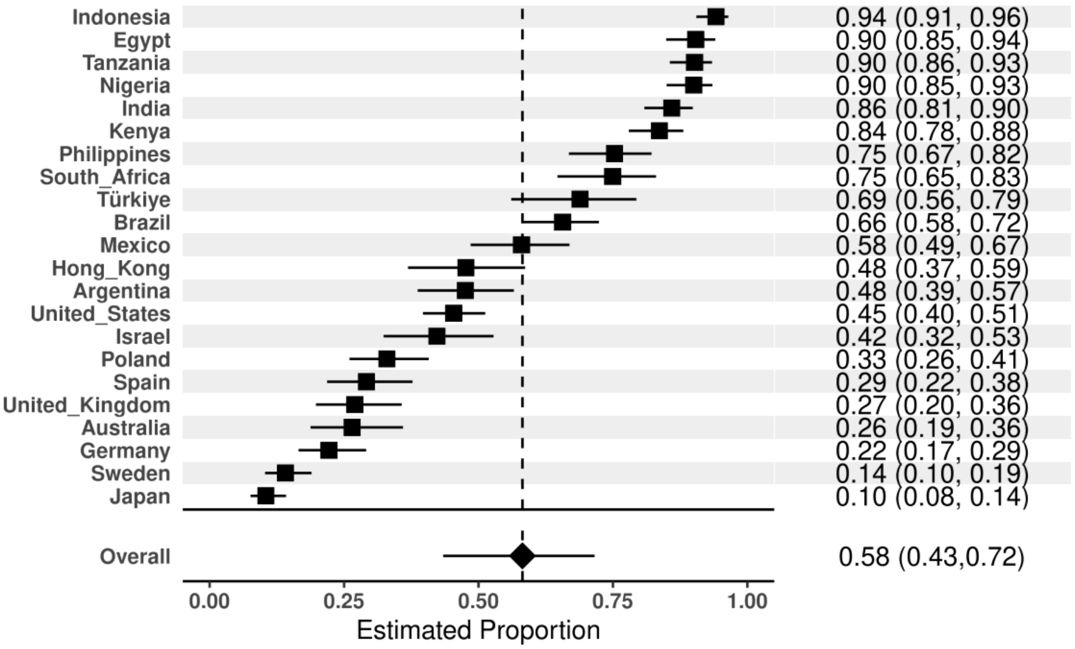

Probability-scale: τ=0.343;
Logit-scale: τ=1.411; Q-profile 95% CI [1.045, 1.922]; I^2=98.63;
Plot is based on back transformed bounds after using approximate logit SE that aren't guarenteed to match the robust SE of a proportion.
Excluded countries: Hong Kong, South Africa, United Kingdom, United States

Figure S20. Forest plot for `Employment status`-`Retired`

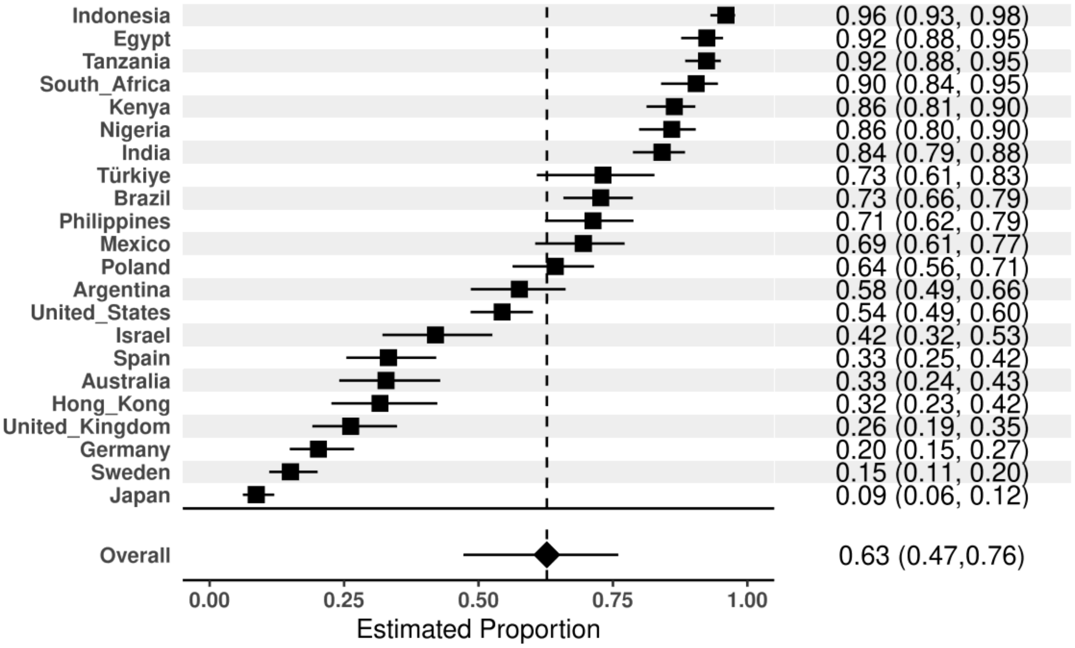

Probability-scale: τ=0.352;
Logit-scale: τ=1.503; Q-profile 95% CI [1.113, 2.047]; I^2=98.75;
Plot is based on back transformed bounds after using approximate logit SE that aren't guarenteed to match the robust SE of a proportion.
Excluded countries: Hong Kong, South Africa, United Kingdom, United States

Figure S21. Forest plot for `Employment status`-`Student`

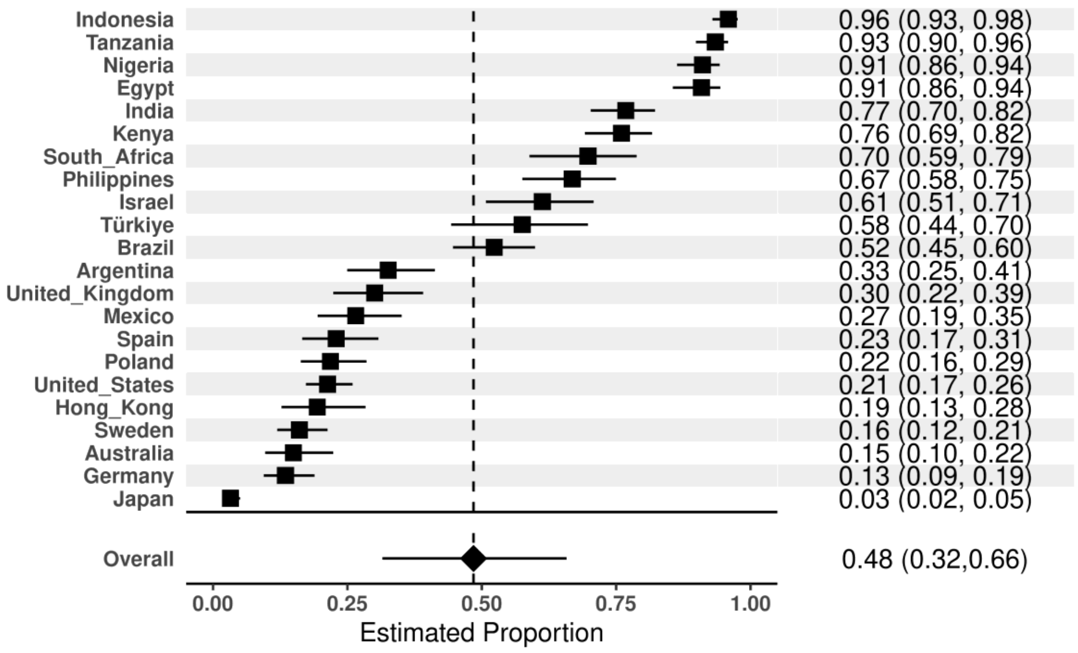

Probability-scale: τ=0.425;
Logit-scale: τ=1.701; Q-profile 95% CI [1.260, 2.314]; I^2=98.98;
Plot is based on back transformed bounds after using approximate logit SE that aren't guarenteed to match the robust SE of a proportion.
Excluded countries: Hong Kong, South Africa, United Kingdom, United States

Figure S22. Forest plot for `Employment status`-`Homemaker`

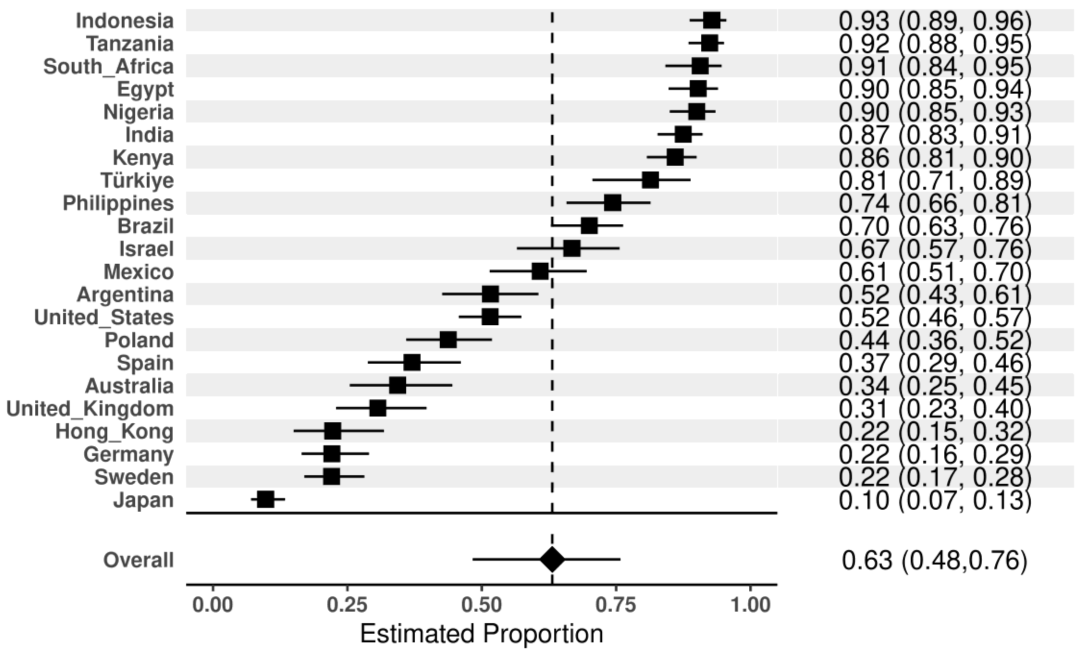

Probability-scale: τ=0.335;
Logit-scale: τ=1.438; Q-profile 95% CI [1.064, 1.958]; I^2=98.66;
Plot is based on back transformed bounds after using approximate logit SE that aren't guarenteed to match the robust SE of a proportion.
Excluded countries: Hong Kong, South Africa, United Kingdom, United States

Figure S23. Forest plot for `Employment status`-`Unemployed and looking for a job`

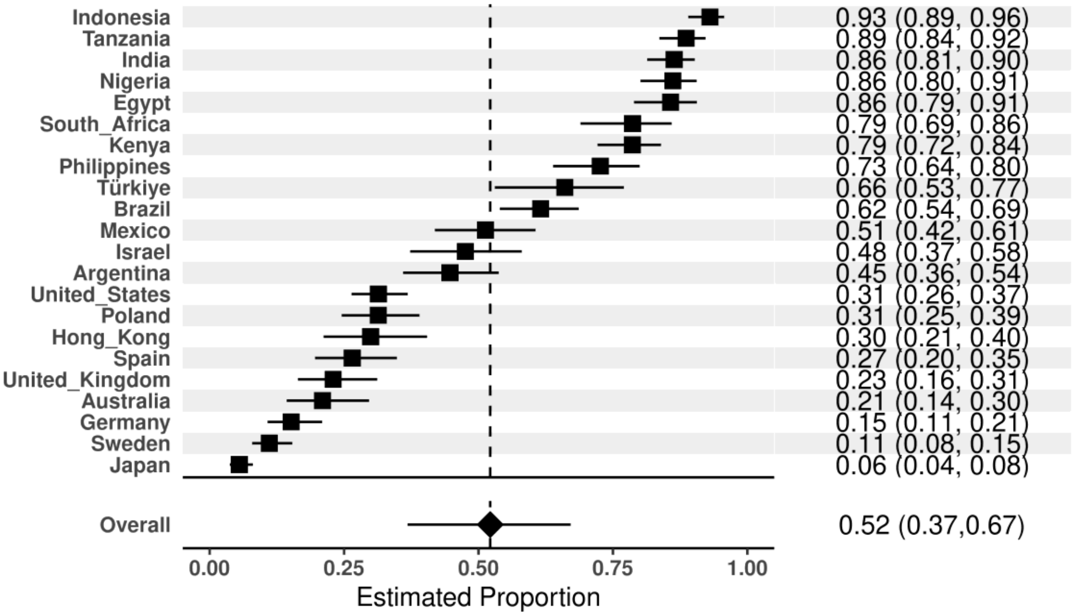

Probability-scale: τ=0.372;
Logit-scale: τ=1.491; Q-profile 95% CI [1.105, 2.030]; I^2=98.74;
Plot is based on back transformed bounds after using approximate logit SE that aren't guarenteed to match the robust SE of a proportion.
Excluded countries: Hong Kong, South Africa, United Kingdom, United States

Figure S24. Forest plot for `Employment status`-`None of these/other`

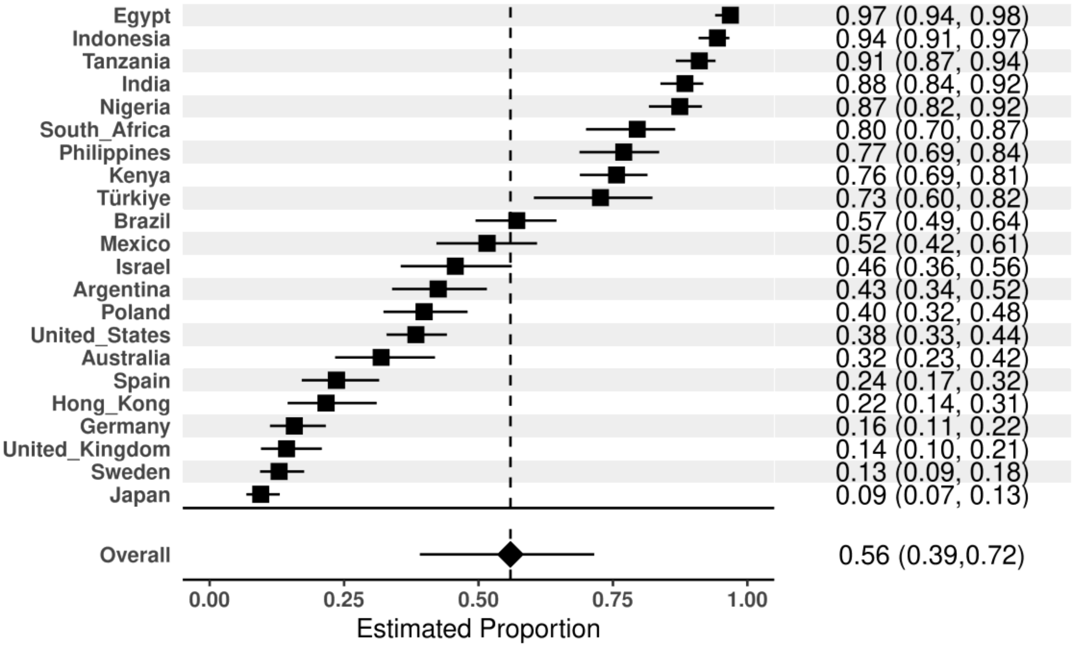

Probability-scale: τ=0.400;
Logit-scale: τ=1.623; Q-profile 95% CI [1.200, 2.207]; I^2=98.92;
Plot is based on back transformed bounds after using approximate logit SE that aren't guarenteed to match the robust SE of a proportion.
Excluded countries: Hong Kong, South Africa, United Kingdom, United States

Figure S25. Forest plot for `Education`-`Up to 8 years`

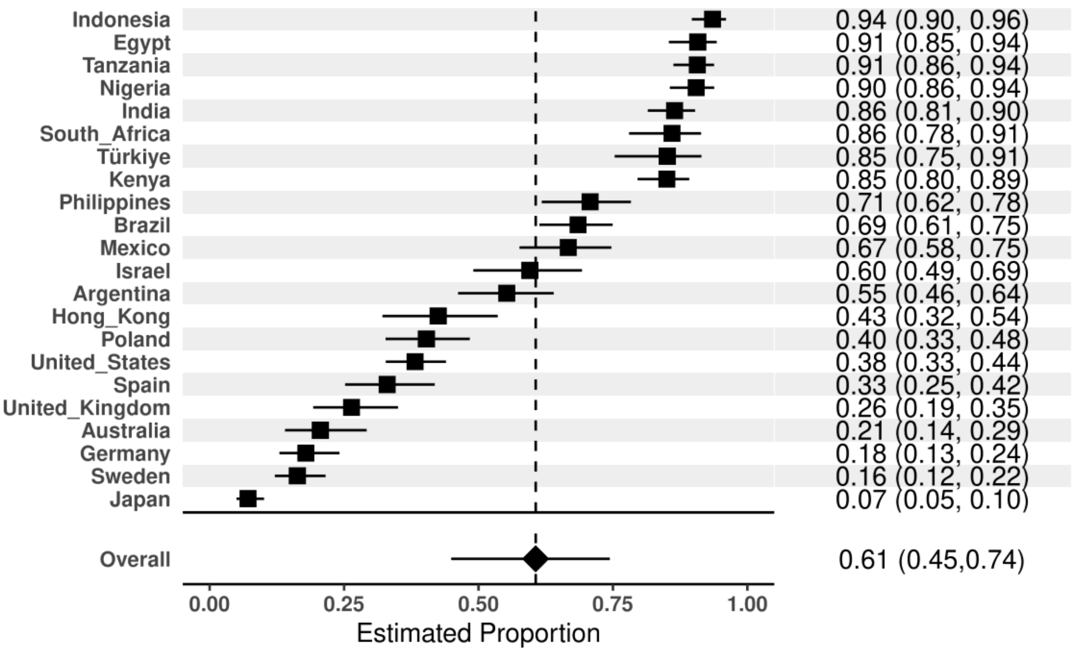

Probability-scale: τ=0.361;
Logit-scale: τ=1.511; Q-profile 95% CI [1.120, 2.058]; I^2=98.77;
Plot is based on back transformed bounds after using approximate logit SE that aren't guarenteed to match the robust SE of a proportion.
Excluded countries: Hong Kong, South Africa, United Kingdom, United States

Figure S26. Forest plot for `Education`-`9-15 years`

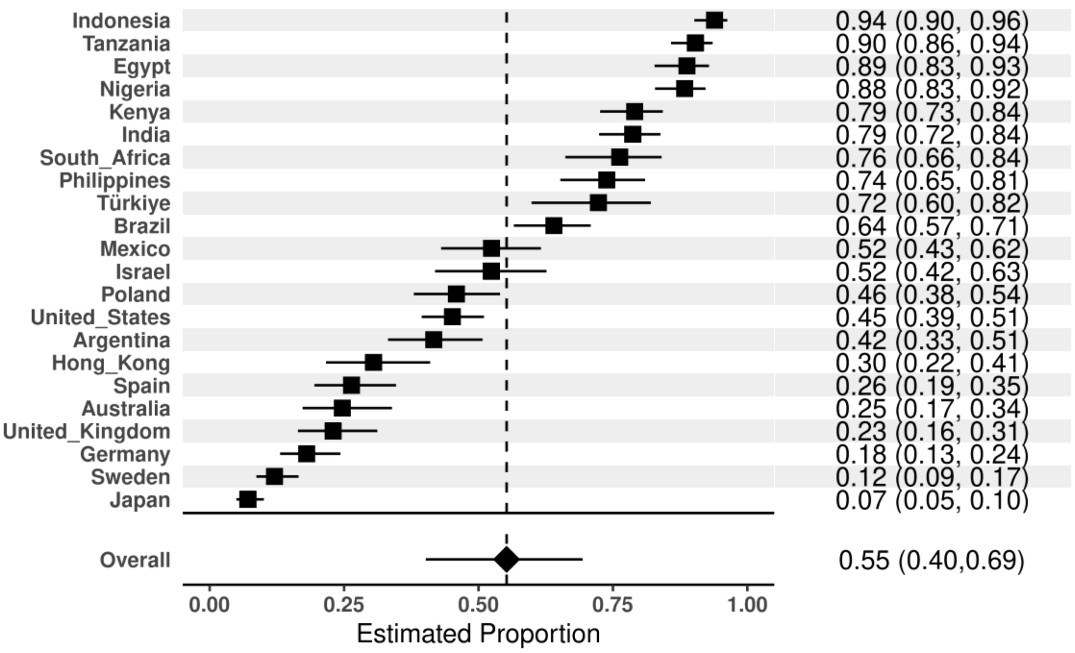

Probability-scale: τ=0.357;
Logit-scale: τ=1.443; Q-profile 95% CI [1.069, 1.965]; I^2=98.69;
Plot is based on back transformed bounds after using approximate logit SE that aren't guarenteed to match the robust SE of a proportion.
Excluded countries: Hong Kong, South Africa, United Kingdom, United States

Figure S27. Forest plot for `Education`-`16+ years`

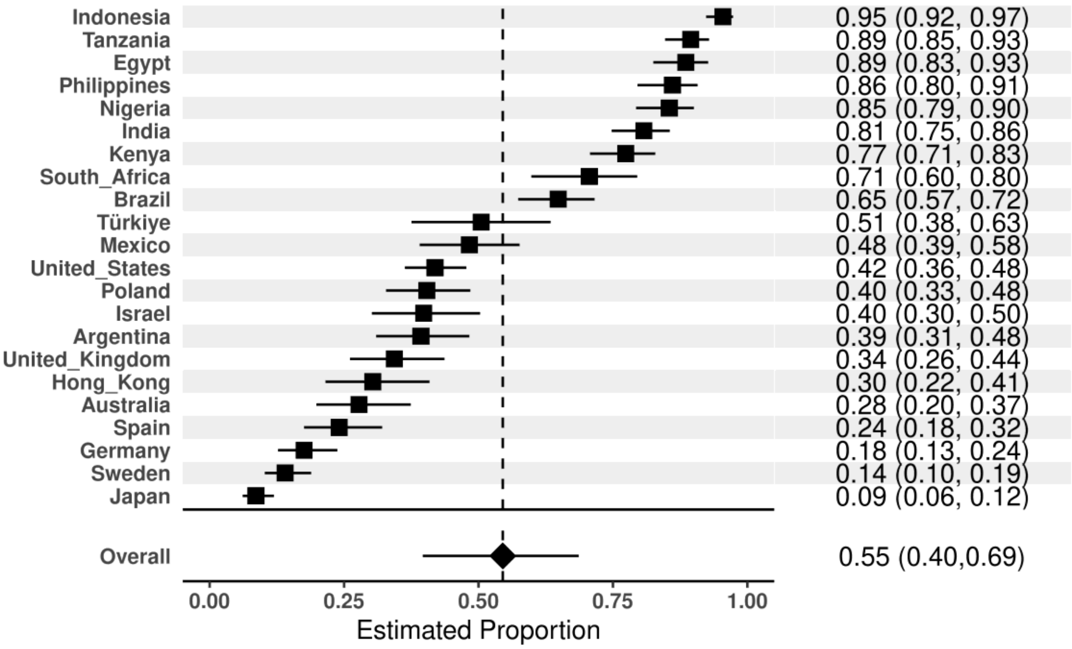

Probability-scale: τ=0.355;
Logit-scale: τ=1.430; Q-profile 95% CI [1.058, 1.947]; I^2=98.67;
Plot is based on back transformed bounds after using approximate logit SE that aren't guarenteed to match the robust SE of a proportion.
Excluded countries: Hong Kong, South Africa, United Kingdom, United States

Figure S28. Forest plot for `Religious service attendance`-`>1/week`

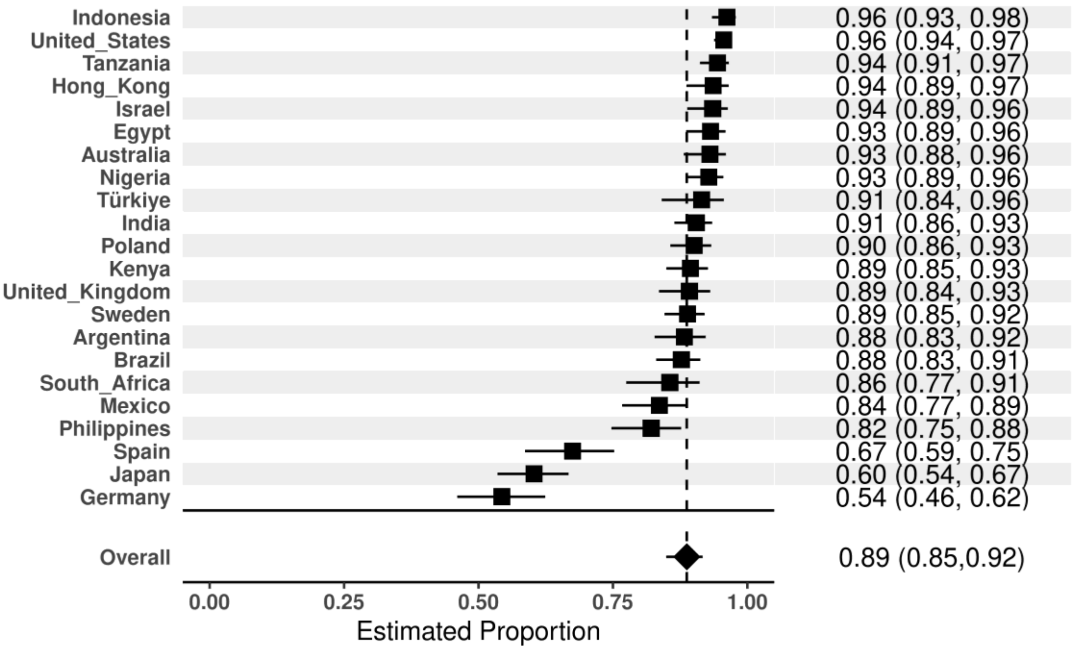

Probability-scale: τ=0.078;
Logit-scale: τ=0.778; Q-profile 95% CI [0.574, 1.078]; I^2=94.42;
Plot is based on back transformed bounds after using approximate logit SE that aren't guarenteed to match the robust SE of a proportion.
Excluded countries: Hong Kong, South Africa, United Kingdom, United States

Figure S29. Forest plot for `Religious service attendance`-`1/week`

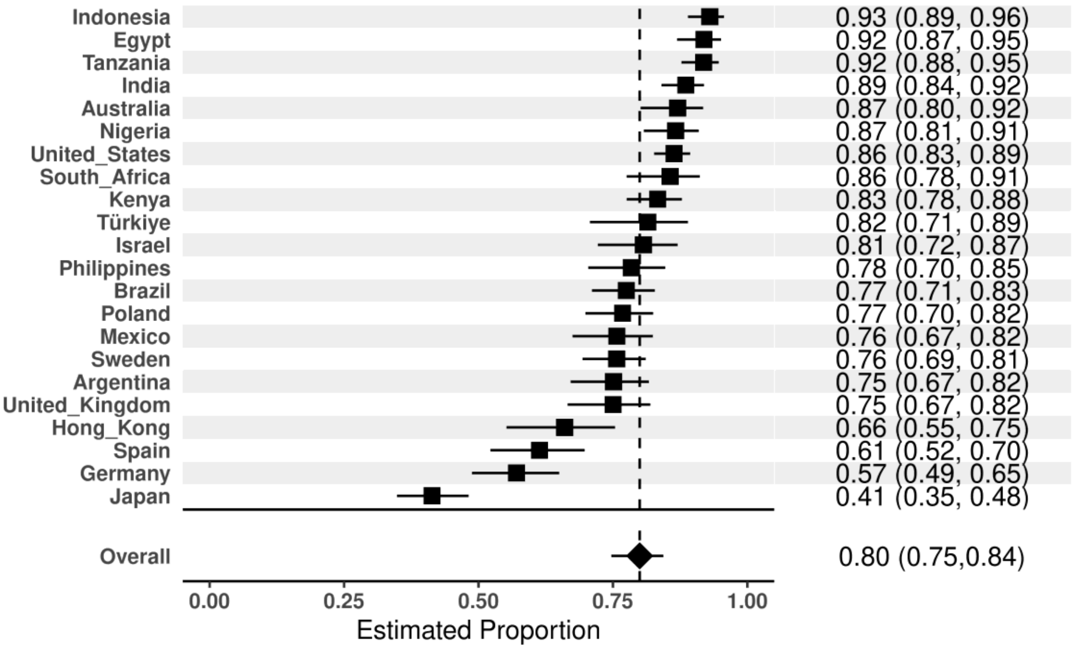

Probability-scale: τ=0.112;
Logit-scale: τ=0.700; Q-profile 95% CI [0.512, 0.967]; I^2=94.45;
Plot is based on back transformed bounds after using approximate logit SE that aren't guarenteed to match the robust SE of a proportion.
Excluded countries: Hong Kong, South Africa, United Kingdom, United States

Figure S30. Forest plot for `Religious service attendance`-`1-3/month`

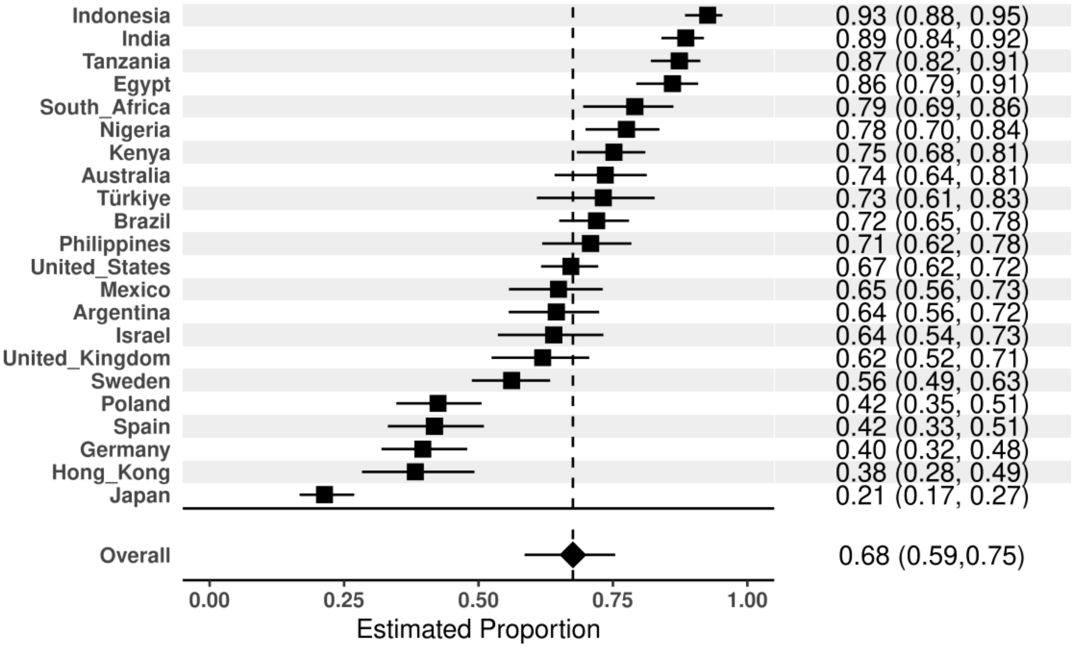

Probability-scale: τ=0.200;
Logit-scale: τ=0.911; Q-profile 95% CI [0.670, 1.247]; I^2=96.98;
Plot is based on back transformed bounds after using approximate logit SE that aren't guarenteed to match the robust SE of a proportion.
Excluded countries: Hong Kong, South Africa, United Kingdom, United States

Figure S31. Forest plot for `Religious service attendance`-`A few times a year`

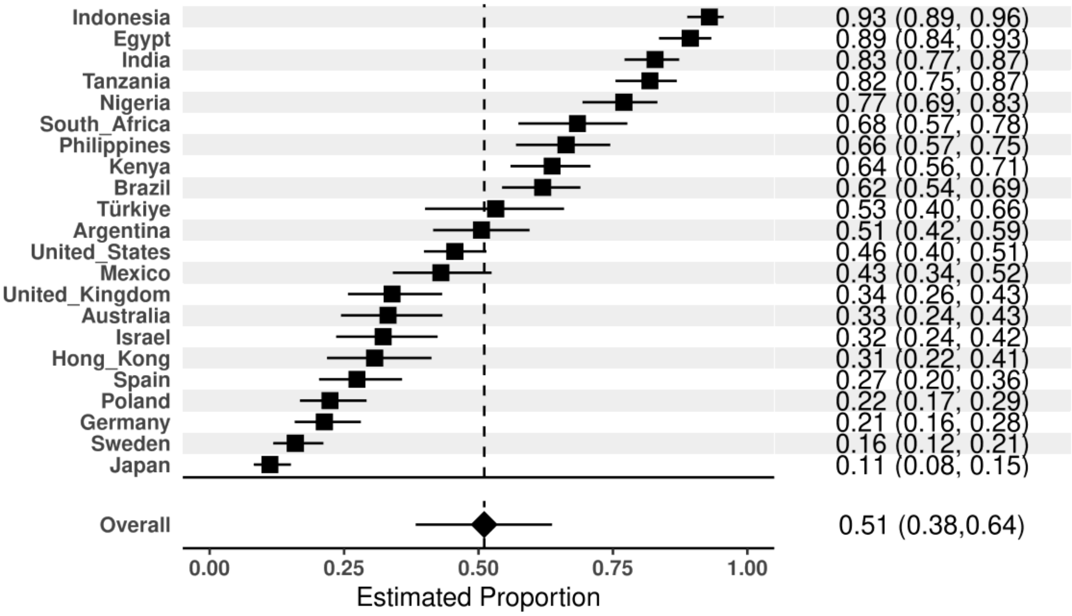

Probability-scale: τ=0.307;
Logit-scale: τ=1.230; Q-profile 95% CI [0.909, 1.676]; I^2=98.29;
Plot is based on back transformed bounds after using approximate logit SE that aren't guarenteed to match the robust SE of a proportion.
Excluded countries: Hong Kong, South Africa, United Kingdom, United States

Figure S32. Forest plot for `Religious service attendance`-`Never`

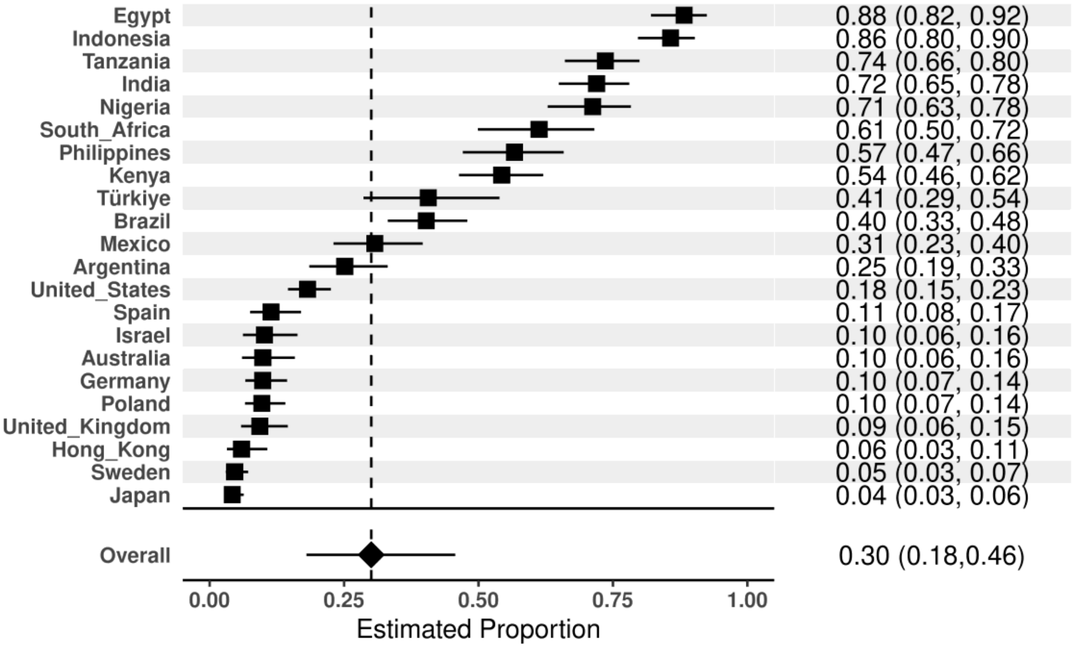

Probability-scale: τ=0.336;
Logit-scale: τ=1.596; Q-profile 95% CI [1.183, 2.173]; I^2=98.81;
Plot is based on back transformed bounds after using approximate logit SE that aren't guarenteed to match the robust SE of a proportion.
Excluded countries: Hong Kong, South Africa, United Kingdom, United States

Figure S33. Forest plot for `Immigration status`-`Born in this country`

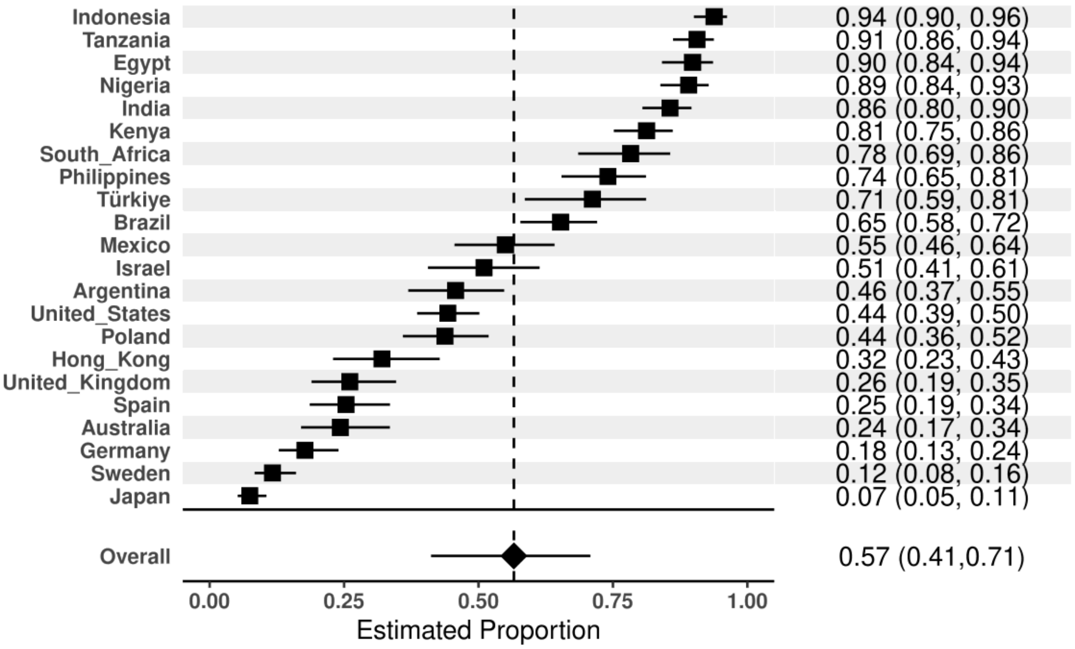

Probability-scale: τ=0.363;
Logit-scale: τ=1.477; Q-profile 95% CI [1.094, 2.011]; I^2=98.72;
Plot is based on back transformed bounds after using approximate logit SE that aren't guarenteed to match the robust SE of a proportion.
Excluded countries: Hong Kong, South Africa, United Kingdom, United States

Figure S34. Forest plot for `Immigration status`-`Born in another country`

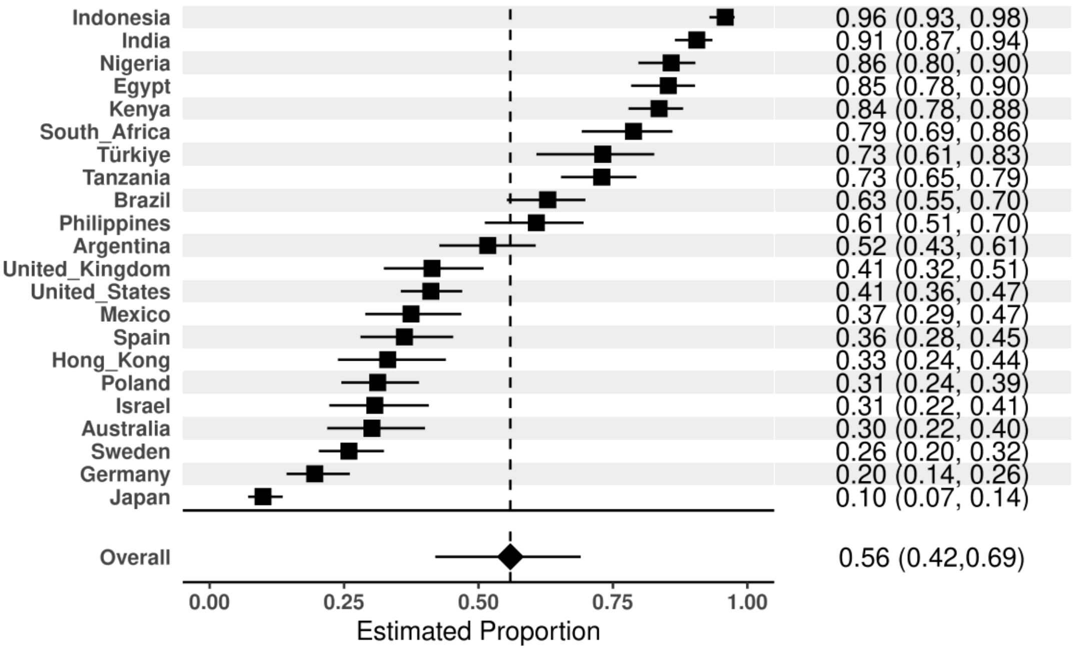

Probability-scale: τ=0.329;
Logit-scale: τ=1.335; Q-profile 95% CI [0.986, 1.818]; I^2=98.51;
Plot is based on back transformed bounds after using approximate logit SE that aren't guarenteed to match the robust SE of a proportion.
Excluded countries: Hong Kong, South Africa, United Kingdom, United States
